# Characteristics of children with severe preschool asthma prior to starting the TIPP study

**DOI:** 10.3389/fped.2025.1558256

**Published:** 2025-03-05

**Authors:** S. Zielen, J. Wosniok, N. Wollscheid, T. Nickolay, C. Grimmel, D. Scheele, F. Sattler, F. Prenzel, M. Lorenz, B. Schaub, C. Lex, M. Dahlheim, J. Trischler, H. Donath, S. Lau, E. Hamelmann, C. Vogelberg, M. Gerstlauer, M. Wetzke, R. Schubert, L. Schollenberger, M. Gappa

**Affiliations:** ^1^ Department for Children and Adolescents, Goethe-University Frankfurt, Frankfurt, Germany; ^2^ Interdisciplinary Center for Clinical Trials (IZKS), University Medical Center Mainz, Mainz, Germany; ^3^ Children’s Hospital, Evangelisches Krankenhaus Düsseldorf, Düsseldorf, Germany; ^4^ Pediatric Allergology, Department of Pediatrics, Dr. von Hauner Children’s University Hospital, LMU Munich-Member of the German Center for Lung Research-DZL-LMU Munich, Munich, Germany; ^5^ Department of Pediatrics, Medical Center, University of Leipzig, Leipzig, Germany; ^6^ Klinik für Kinder- und Jugendmedizin, Universität Jena, Jena, Germany; ^7^ Department for Pediatric Cardiology, Intensive Care and Neonatology, University Medicine, Göttingen, Germany; ^8^ Kinderlungen-Facharzt, Gemeinschaftspraxis in Mannheim, Mannheim, Germany; ^9^ Department of Respiratory Medicine, Immunology and Critical Care Medicine, Charité Universitätsmedizin Berlin, Berlin, Germany; ^10^ Department of Pediatrics, Children’s Center Bethel, University Medicine, Bielefeld, Germany; ^11^ Klinik und Poliklinik für Kinder- und Jugendmedizin, Universitätsklinikum Carl Gustav Carus an der Technischen Universität, Dresden, Germany; ^12^ Kinderklinik, Universitätsklinikum Augsburg, Augsburg, Germany; ^13^ Pediatric Pneumology, Allergology, Neonatology, Hannover Medical School, Hannover, Germany

**Keywords:** preschool asthma, uncontrolled asthma, PACD, hospitalization, asthma phenotypes, tiotropium bromide

## Abstract

**Objective:**

Children with preschool asthma suffer disproportionally more often from severe asthma exacerbations with emergency visits and hospital admissions than school children. However, there are only a few reports on characteristics, hospitalization, phenotypes and symptoms in this age cohort.

**Patients and methods:**

This analysis of an ongoing prospective trial of Tiotropium bromide in preventing severe asthma exacerbations (the TIPP study) assessed baseline characteristics, hospitalizations and symptoms in 100 children with severe preschool asthma. Children aged 1–5 years were analyzed at study enrollment and daily symptoms were recorded by an electronic diary [Pediatric Asthma Caregiver Diary (PACD)] for the following four weeks until randomization.

**Results:**

At enrollment, the total number of severe asthma exacerbations, defined as three days systemic steroid use or hospitalization in the last 24 months, was mean (±SD) 5.8 ± 5.7 and the test for respiratory and asthma control in kids (TRACK) was mean 46.9 ± 19.0. Daily recording of symptoms by the PACD revealed that only 7 patients were controlled at randomization, whereas 35 were partially and 58 were uncontrolled according to GINA.

**Conclusion:**

Despite protective therapy with inhaled corticosteroids (ICS), most children of this severe asthma cohort were only partially or uncontrolled according to GINA guidelines.

## Research in context

• Evidence before this study
  We reviewed literature on PubMed before writing this manuscript for recent publications on severe asthma in preschool children. Despite this high disease burden, limited detailed reports on characteristics, hospitalization, phenotypes and symptoms in this particularly severe asthma cohort are available. The preschool asthma cohort is often described as exacerbation prone with relatively limited impairment.
• Added value of this study
  This analysis provides additional evidence that hospitalizations are frequent, and the electronic diary record showed a high symptom burden despite regular ICS treatment.
• Implications of all the available evidence
  Despite protective therapy with ICS, most children of this severe preschool asthma cohort are only partial or uncontrolled according to GINA guidelines. Current care and treatment are not sufficient to adequately control severe asthma in preschool children.

## Introduction

Asthma is the most common chronic disease in children, imposing a high lifetime burden on individuals, their caregivers, and healthcare systems (1, 2). The prevalence of childhood asthma has increased over the last 20 years, most likely due to a greater awareness of this condition and changes in diagnostic practice (1, 3). The global prevalence of diagnosed current asthma in children 5 years and younger cannot be well estimated due to the lack of international consensus on diagnostic criteria (4). A recent ERS task force suggested that the criteria used to define wheezing disorders in preschool children should include age of diagnosis (0 to <6 years) and confirmation of wheezing on more than one occasion (5).

Of note is the diagnostic uncertainty among young children in whom wheezing is more likely to be associated with lower respiratory tract infection and often is transient (5–9). Although asthma prevalence is higher in school children than in toddlers, severe asthma exacerbations with emergency admissions and hospitalizations occur disproportionally more often in preschool asthma (10, 11). In addition, young children are more susceptible to adverse outcomes than older children, due to their small airways and possibly increased bronchial airway reactivity (12, 13). Respiratory distress in the setting of infection can rapidly become life-threatening. This explains the relatively high use of emergency visits and hospitalizations among preschool children.

Many children with preschool asthma are not well controlled by inhaled corticosteroids (ICS), which is a significant healthcare concern (14). Failure to control asthma has a negative impact on patients' quality of life, and increases the risk of future exacerbations, which in turn leads to an increased need for medical care and higher costs (15, 16). The management of children with preschool asthma is complicated by a paucity of high-quality clinical trials in this age group. The preschool asthma population is often described as being susceptible to exacerbations with relatively limited impairment (17). However, young children with frequent severe exacerbations are common and caregivers are complaining about frequent coughing and wheezing and missed days in daycare center, especially in winter (18, 19).

Although studies are limited, data suggest that an asthma-like inflammation (presence of eosinophils and allergic sensitization) may be present at a very early age in some children with recurrent wheeze (20, 21). However, approximately half of preschool asthma patients do not present with TH2- inflammation and may show a TH1-like neutrophilic airway inflammation (11). These children are often younger and suffer from severe RSV and Rhinovirus infections and may outgrow this condition at school-age (11, 22–24). Number of exacerbations in the last 24 months, sensitization and analysis of eosinophils are useful for predicting future exacerbations and can identify children who are most likely to respond to daily ICS treatment (11, 25, 26). Nevertheless, current treatment guidelines are not based on the underlying asthma phenotype (27). This might explain why ICS is effective in reducing severe exacerbations by only 36% in preschool asthma patients (11, 27). At present, there are insufficient data to recommend additional controller therapies in this age group, such as combinations of ICS with LABAs or LAMAs (28, 29). In the Respimat, Tiotropium bromide has been approved by the European Medicines Agency for use in adults, and adolescents (12 years and older), and in children (6–11 years), as add-on maintenance treatment for severe asthma, who experienced at least one asthma exacerbation in the preceding year. Adding Tiotropium to ICS might be a new promising treatment option for severe uncontrolled preschool asthma (30, 31). However, the number of patients included in both studies was small, and further research is required to confirm the beneficial effect on asthma phenotypes, symptom control and exacerbations in this age group.

Accordingly, we present the characteristics of a severe preschool asthma cohort, such as number of severe exacerbations, level of asthma control, daily asthma symptoms monitored with an electronic diary for four weeks, and TH2-phenotype at enrollment in the ongoing TIPP study (TIPP study: Tiotropium as add-on to ICS; EudraCT 2021-000190-81).

## Methods

The TIPP study is an ongoing, prospective multicenter placebo-controlled trial to evaluate the efficacy and safety of Tiotropium inhalation solution 2.5 µg daily in preventing severe asthma exacerbations in partial and uncontrolled preschool asthma. It is planned to enroll approximately 150 patients at 13 study sites in Germany. The present manuscript describes the baseline characteristics of the first 100 patients enrolled in the study. The study was registered at the “EU Clinical Trials Registry” (EudraCT 2021-000190-81, https://www.clinicaltrialsregister.eu/) and transitioned according to EU Clinical Trial Regulation 536/2014 (EU CT 2024-513916-84-00). The complete protocol of the study is provided in the Supplementary Material. Ethics approval was obtained from the leading Ethics Committee of the Goethe-University in Frankfurt (application number 2021-443-AMG) and all participating Ethics Committees. Written informed consent of both parents, or the custodial parent, was obtained before participation of the child.

All children were analyzed at study enrollment (visit 1) and daily symptoms were recorded with an electronic diary [Pediatric Asthma Caregiver Diary (PACD)] for the following four weeks until randomization (visit 2).

### Main diagnosis for study entry

Patients aged 1–5 years with a history of at least one severe asthma exacerbation requiring hospitalization in the last 24 months and/or treated with 2 courses of systemic steroids before visit 1. In addition, all patients must have been on maintenance treatment with an ICS at a stable dose for at least 4 weeks before visit 1. At visit 1, and during the following four weeks of observation until visit 2, patients had to be symptomatic (partly or uncontrolled)—despite their current maintenance treatment—to be subsequently randomized to either the control group (placebo) or the intervention group (Tiotropium bromide).

### Main inclusion criteria

Patients meeting the following criteria were recruited for the study:

1. All patients' parents (or legal guardians) must sign and date an informed consent, consistent with ICH-GCP guidelines and local legislation prior to participation in the trial.
2. Male or female patients with preschool asthma aged between 1 and 5 years (<6 at visit 1).
3. Physician diagnosed asthma with at least 6 months' history of asthma symptoms, including but not limited to wheezing, cough, and/or shortness of breath.
4. All patients must have been on maintenance treatment with an ICS at a stable dose, either as mono treatment or in combination with another controller medication, for at least 4 weeks before screening (visit 1) and randomization (visit 2).
5. Patient was hospitalized due to acute severe asthma and/or was treated with 2 courses of systemic steroids (three days of oral steroids or one day of rectal prednisolone 100 mg) in the last 24 months before visit 1.
6. Caregivers had to record daily symptoms by an electronic diary [Pediatric Asthma Caregiver Diary (PACD)] between visit 1 and visit 2 until randomization.
7. All patients must be symptomatic (partly or uncontrolled) as defined by the GINA guideline for children aged 5 years and in the week prior to randomization despite treatment with ICS between visit 1 and visit 2.
8. Ability of parents/legal guardians to understand nature, importance, and individual consequences of the trial.
9. Patients must be able to inhale from the Respimat® inhaler (with a spacer).

### Main exclusion criteria

Patients presenting with any of the following criteria will not be included in the trial:

1. Patients with a significant disease other than asthma such as, but not limited to, the following diagnoses: cystic fibrosis, bronchopulmonary dysplasia, primary immune-deficiency, congenital heart disease, parasitic disease, and foreign body aspiration.
2. Patients with clinically relevant abnormal screening hematology or blood chemistry will be excluded if the abnormality defines a significant disease as defined in the exclusion criterion.
3. Patients with any acute asthma exacerbation or respiratory tract infection in the four weeks before visit 1 (screening). If an acute exacerbation or respiratory tract infection occurs 4 weeks before visit 1, this visit can be postponed for 4 weeks.
4. Patients with a history of congenital or acquired heart disease, or patients who have been hospitalized for cardiac syncope or failure during the past year.
5. Medical or psychological conditions that would jeopardize an adequate and orderly completion of the trial.
6. Patients with known hypersensitivity to anticholinergic drugs, or any other components of the Tiotropium inhalation solution.

### Outcome parameters

Number of hospitalizations, number of systemic corticosteroids cycles (SCS) (defined as 3 days SCS use), number of exacerbations, night-time awakenings due to asthma symptoms as assessed by the patient`s electronical diary/PACD, percentage of days with asthma symptoms, percentage of days with use of salbutamol rescue medication, health utilization (missed days in daycare center, number of physician visits), were evaluated as outcome parameters.

To define the asthma phenotype, blood eosinophils, total Immunoglobulin E (IgE), and sIgE were measured. Asthma phenotypes were defined as recently described (26, 32):

1. **Sensitization in patients** (TH2-phenotype/possible allergy) was defined by measuring sIgE ≥ 0.75 K/UL to any of the tested allergens like birch, grass, mites, alternaria, cladosporium, cat, dog, milk, egg and peanut.
2. **Non-allergic asthma** was defined by absence of sIgE levels > 0.75 K/UL.
3. Patients without sIgE data (missing values) were classified as **not defined**.

#### Level of education of parents and/or caretakers

The information for parents and caregivers about the Tipp study, current asthma treatment in children 1–5 years and appropriate use of asthma medications like Salbutamol, Fluticasone and Fluticasone + LABA (Viani mite) were displayed during the recruitment period on two specialized websites. In addition, all parents and caregivers were educated about the need to inhale ICS on a regular basis. In addition, during every visit the inhalation technique was trained and the importance of adherence to treatment was taught by the study nurses and physicians. Children 1–<4 years inhaled ICS and tiotropium via an aerochamber with a face mask. In children >4 years, children and parents were trained to inhale via an areochamber using the mouthpiece.

### PACD

The PACD was used to evaluate daily asthma symptoms in children aged 1–5 years (33). The diary consists of three questions to be answered each morning when the child wakes up, and seven questions to be answered each evening, right after the child goes to bed. The combined daytime score is the average of scores from questions 4–7 in the diary which are questions regarding severity of cough, wheezing, trouble breathing, and interference with activities, scores for each question range from 0 (best) to 5 (worst). The PACD questions were collected electronically by a self-developed diary App (https://apps.apple.com/de/app/tipp-diary/id1598597989).

### TRACK test

The test for respiratory and asthma control in kids (TRACK) was used to evaluate the respiratory and asthma control at visit 1 (34).

### Laboratory parameters

The following parameters were analyzed at visit 1: Blood count with eosinophils, a safety lab (CRP, liver enzymes, creatinine), and total IgE and sIgE to 10 allergens [birch, grass, house dust mites (*Dermatophagoides pteronyssinus*), alternaria, cladosporium, cat, dog, milk, egg and peanut].

### Statistics

Basic descriptive statistics including absolute and relative frequency distributions are reported. Clinical characteristics were compared by exploratory *F*-tests, chi-squared tests or Fisher's exact tests, if appropriate. Statistical analysis was performed with SAS Version 9.4.

## Results

Between February 2022 and March 2024, 100 patients with severe asthma defined by hospitalization due to acute severe asthma and/or treatment with 2 courses of systemic steroids in the last 24 months were enrolled at 13 study centers. Of these patients, 58 (58%) were males. The male predominance was more pronounced in the sensitized patients, 18 (62%) vs. female, 11 (38%). In addition, the group of non-sensitized patients was significantly younger (*p* < 0.01). Accordingly, their size and weight were smaller (Table 1). All other parameters were equally distributed.

**Table 1:** Baseline characteristics.

Variable	Value	Sensitized (*N* = 29)	Non-sensitized (*N* = 58)	Not defined (*N* = 13)	Total (*N* = 100)	*p*-value
Age (years)	Mean ± SD	4.2 ± 0.9	2.9 ± 1.2	2.9 ± 1.5	3.3 ± 1.3	<.0001
Gender	Male	18 (62%)	32 (55%)	8 (62%)	58 (58%)	0.7968
	Female	11 (38%)	26 (45%)	5 (38%)	42 (42%)	
Race	White	23 (79%)	48 (83%)	12 (92%)	83 (83%)	0.8545
	Asian	1 (3%)	1 (2%)	0 (0%)	2 (2%)	
	Black	0 (0%)	0 (0%)	0 (0%)	0 (0%)	
	Other	5 (17%)	9 (16%)	1 (8%)	15 (15%)	
Height (cm)	Mean ± SD	105.8 ± 7.4	94.6 ± 10.6	96.9 ± 12.1	98.2 ± 11.0	<.0001
Weight (kg)	Mean ± SD	17.49 ± 3.10	14.91 ± 3.10	14.67 ± 3.26	15.64 ± 3.31	0.0012
Gestational age (weeks)	Mean ± SD	38.7 ± 2.6	38.6 ± 2.4	39.8 ± 0.6	38.8 ± 2.4	0.2725
Breastfeeding (n)	*N* (%)	26 (90%)	45 (82%)	7 (64%)	78 (82%)	0.1587
Co-Morbidities						
Allergic rhinitis (n)	*N* (%)	9 (31%)	7 (12%)	1 (8%)	17 (17%)	0.0758
Atopic dermatitis (n)	*N* (%)	3 (10%)	4 (7%)	0 (0%)	7 (7%)	0.6291
No Household pet	*N* (%)	20 (69%)	41 (71%)	9 (69%)	70 (70%)	0.9843
Dog	*N* (%)	3 (10%)	7 (12%)	3 (23%)	13 (13%)	0.4985
Cat	*N* (%)	6 (21%)	11 (19%)	1 (8%)	18 (18%)	0.5728
Guinea pig	*N* (%)	0 (0%)	1 (2%)	0 (0%)	1 (1%)	0.6937
Household/second hand smoke	*N* (%)	6 (21%)	15 (26%)	3 (23%)	24 (24%)	0.8648

The maintenance therapy showed that ICS mono treatment was given in 27 (27%) patients, an ICS + LABA combination in 53 (53%) patients and an additional controller treatment with LTRA was given in 20 (20%) patients only (Table 2).

**Table 2:** Baseline characteristics—vital signs, physical examination, GINA level and treatment.

Variable	Value	Sensitized (*N* = 29)	Non-sensitized (*N* = 58)	Not defined (*N* = 13)	Total (*N* = 100)	*p*-value
Result of physical examination	Abnormal findings, NOT clinically significant	7 (24%)	4 (7%)	3 (25%)	14 (14%)	0.0611
	Abnormal findings, clinically significant	4 (14%)	14 (25%)	0 (0%)	18 (18%)	
GINA: daytime symptoms	*N* (%)	21 (72%)	47 (81%)	10 (77%)	78 (78%)	0.6546
GINA: limitations of activities	*N* (%)	19 (66%)	33 (57%)	8 (62%)	60 (60%)	0.7359
GINA: nocturnal symptoms/awakening	*N* (%)	17 (59%)	45 (78%)	8 (62%)	70 (70%)	0.1480
GINA: need for reliever/rescue treatment	*N* (%)	14 (48%)	29 (50%)	8 (62%)	51 (51%)	0.7093
Level of asthma symptom control	Well controlled	3 (10%)	2 (3%)	2 (15%)	7 (7%)	0.2935
	Partly controlled	11 (38%)	22 (38%)	2 (15%)	35 (35%)	
	Uncontrolled	15 (52%)	34 (59%)	9 (69%)	58 (58%)	
Time since asthma diagnosis (years)	Mean ± SD	1.6 ± 1.2	1.2 ± 0.9	1.0 ± 0.9	1.3 ± 1.0	0.1522
ICS mono	*N* (%)	9 (31%)	15 (26%)	3 (23%)	27 (27%)	0.8273
ICS mono dose [µg/day]	Mean ± SD	–	–	–	203.0 ± 99.2	–
ICS + LTRA	*N* (%)	0 (0%)	3 (5%)	0 (0%)	3 (3%)	0.3263
ICS + LABA	*N* (%)	17 (59%)	29 (50%)	7 (54%)	53 (53%)	0.7479
ICS + LABA dose [ICS µg/day]	Mean ± SD	–	–	–	163.4 ± 95.6	–
ICS + LABA + LTRA	*N* (%)	3 (10%)	11 (19%)	3 (23%)	17 (17%)	0.4943

At enrollment, the number of asthma exacerbations and SCS cycles of the previous 12 and 24 months were recorded. As shown in Table 3, the total number of severe asthma exacerbations was mean (±SD) 5.8 ± 5.7. Accordingly, the TRACK test for respiratory and asthma control in kids was 46.9 ± 19.0, indicating severe uncontrolled preschool asthma. None of the children was fully controlled at enrollment.

**Table 3:** Baseline characteristics—asthma exacerbations and TRACK test before visit 1.

Variable	Value	Sensitized (*N* = 29)	Non-sensitized (*N* = 58)	Not defined (*N* = 13)	Total (*N* = 100)	*p*-value
Number of inpatient stays >12–24 months before V1	Mean ± SD	0.7 ± 1.1	0.8 ± 1.5	1.4 ± 3.1	0.9 ± 1.7	0.5001
No cycles of systemic steroids >12–24 months	Mean ± SD	1.3 ± 1.7	1.3 ± 2.3	0.7 ± 1.5	1.2 ± 2.0	0.6411
Number of inpatient stays <=12 months before V1	Mean ± SD	1.0 ± 1.1	1.5 ± 1.9	2.8 ± 2.9	1.5 ± 1.9	0.0172
*N* of cycles of systemic						
Steroids <=12 months	Mean ± SD	1.9 ± 1.7	2.8 ± 2.8	2.7 ± 2.1	2.5 ± 2.5	0.2959
Total number of severe asthma						
Exacerbations before (n)	Mean ± SD	4.5 ± 2.9	6.2 ± 5.9	7.2 ± 8.4	5.8 ± 5.7	0.2766
TRACK—Test Total Score	Mean ± SD	50.5 ± 18.5	46.1 ± 18.1	41.9 ± 23.9	46.9 ± 19.0	0.3670

### Asthma phenotype

The determination of the asthma TH-2 phenotype (complete dataset) was possible in 87% of the 100 enrolled patients. The phenotype distribution of patients based on sIgE is shown in Table 4: Sensitized patients had significantly higher levels of total IgE and higher eosinophils than patients with non-allergic asthma (IgE 260.95 ± 335.46 vs. 27.90 ± 37.59; *p* < 0.01; eosinophils 0.723 ± 1.275 vs. 0.377 ± 0.826, n.s.).

**Table 4:** Baseline characteristics—allergy diagnostic/total IgE, eosinophils and specific IgE.

Variable	Value	Sensitized (*N* = 29)	Non-sensitized (*N* = 58)	Not defined (*N* = 13)	Total (*N* = 100)	*p*-value
Total IgE kU/L	Median (Q1–Q3)	118.00 (62.00–459.00)	16.00 (16.00–16.00)	16.00 (16.00–16.00)	16.00 (16.000–100.00)	<.0001
Patients with IgE >=100 (kU/L)	*N* (%)	18 (64%)	4 (7%)	0 (0%)	22 (26%)	<.0001
Eosinophils 10^3^/µl	Median (Q1–Q3)	0.400 (0.310–0.515)	0.215 (0.12–0.33)	0.16 (0.16-.016)	0.300 (0.16–0.43)	0.4165
Patients with EOS >300 µl	*N* (%)	18 (75%)	11 (29%)	—	29 (46%)	—
Specific IgE results						
Birch sIgE (KU/L)	Median (Q1–Q3)	1.8 (0.13–28.80)	0.10 (0.10–0.10)	—	0.10 (0.10–0.35)	—
Patients with Birch sIgE >=0.75 kU/L	*N* (%)	17 (63%)	0 (0%)	—	17 (21%)	—
Grass sIgE (KU/L)	Median (Q1–Q3)	0.45 (0.12–1.73)	0.10 (0.10–0.10)	—	0.10 (0.10–0.35)	—
Patients with Grass sIgE >=0.75 kU/L	*N* (%)	9 (32%)	0 (0%)	—	9 (11%)	—
Dermatophagoides pteronyssinus sIgE (KU/L)	Median (Q1–Q3)	0.99 (0.15–16.10)	0.10 (0.10–0.10)	—	0.10 (0.10–0.35)	—
Patients with Dermatophagoides pteronyssinus sIgE >=0.75 kU/L	*N* (%)	14 (52%)	0 (0%)	—	14 (17%)	—
Cat sIgE (KU/L)	Median (Q1–Q3)	0.35 (0.10–10.40)	0.10 (0.10–0.10)	—	0.10 (0.10–0.35)	—
Patients with Cat sIgE >=0.75 kU/L	*N* (%)	11 (38%)	0 (0%)	—	11 (13%)	—
Dog sIgE (KU/L)	Median (Q1–Q3)	0.35 (0.19–0.69)	0.10 (0.10–0.10)	—	0.10 (0.10–0.35)	—
Patients with Dog sIgE >=0.75 kU/L	*N* (%)	6 (21%)	0 (0%)	—	6 (7%)	—
Cladosporium sIgE (KU/L)	Median (Q1–Q3)	0.10 (0.10–0.12)	0.10 (0.10–0.10)	—	0.10 (0.10–0.10)	—
Patients with Cladosporium sIgE >=0.75 kU/L	*N* (%)	0 (0%)	0 (0%)	—	0 (0%)	—
Alternaria sIgE (KU/L)	Median (Q1–Q3)	0.10 (0.10–0.35)	0.10 (0.10–0.10)	—	0.10 (0.10–0.14)	—
Patients with Alternaria sIgE >=0.75 kU/L	*N* (%)	3 (10%)	0 (0%)	—	3 (4%)	—
Cow`s milk sIgE (KU/L)	Median (Q1–Q3)	0.29 (0.12–0.53)	0.10 (0.10–0.16)	—	0.10 (0.10–0.35)	—
Patients with	*N* (%)	6 (21%)	0 (0%)	—	6 (7%)	—
Hen`s egg sIgE (KU/L)	Median (Q1–Q3)	0.31 (0.16–0.78)	0.10 (0.10–0.12)	—	0.10 (0.10–0.35)	—
Patients with	*N* (%)	7 (25%)	0 (0%)	—	7 (8%)	—
Peanut sIgE (KU/L)	Median (Q1–Q3)	0.35 (0.10–1.29)	0.10 (0.10–0.10)	—	0.10 (0.10–0.35)	—
Patients with Peanut sIgE >=0.75 kU/L	*N* (%)	9 (33%)	0 (0%)	—	9 (11%)	—

### Allergen sensitization

The distribution of sensitization to pollen, HDM, animal epithelial and food allergens is shown in Table 4. Birch pollen sensitization was found in 17 (63%) sensitized patients. HDM sensitization was present in 14 (52%) patients and cat sensitization in 11 (38%) patients. Peanut sensitization was found in 9 (33%) patients and egg sensitization in 7 (25%) patients.

### Symptoms and treatment between V1 and V2

The compliance of the TIPP App diary use was high with a median (Q1–Q3) of 96% (86%–100%) documented days. As shown in Table 5, patients had symptoms on mean (± SD) 15.7 ± 8.2 days and night-time awakenings in mean 4.9 ± 5.2 of 28 days of observation. Daily salbutamol use was recorded at mean 5.8 ± 5.4 days and median (Q1–Q3) number of missed days in daycare was 1.0 (0–5 days (Table 5). Although the patients in TIPP are being cared for, healthcare utilization was high. The number of pediatric family contacts were 38 (41%), other health contacts 9 (10%) and emergency visits 8 (9%) during the four weeks observation (Table 5).

**Table 5:** Asthma symptoms and health care utilization between visit 1 and visit 2.

Variable	Value	Sensitized (*N* = 29)	Non-Sensitized (*N* = 58)	Not defined (*N* = 13)	Total (*N* = 100)	*p*-value
Days with asthma	Mean ± SD	13.1 ± 7.9	17.9 ± 7.8	12.2 ± 8.4	15.7 ± 8.2	0.0162
Percentage of days without asthma (%)*	Mean ± SD	46.4 ± 32.8	30.7 ± 29.2	46.0 ± 35.6	37.6 ± 31.7	0.0805
Days with Salbutamol use	Mean ± SD	4.6 ± 4.3	7.0 ± 6.0	3.1 ± 2.7	5.8 ± 5.4	0.0543
Number of night-time awakenings due to asthma symptoms	Mean ± SD	4.1 ± 4.1	5.9 ± 5.8	2.6 ± 3.0	4.9 ± 5.2	0.1143
Number of missed days in daycare	Median (Q1–Q3)	0.5 (0–5)	2.0 (0–6)	1.5 (0–5)	1.0 (0–5)	0.3536
Healthcare utilization						
Appointment emergency doctor/emergency room?	*N* (%)	3 (12%)	5 (9%)	0 (0%)	8 (9%)	0.4894
Hospitalization	*N* (%)	1 (4%)	4 (7%)	0 (0%)	5 (5%)	0.5417
Pediatrician family doctor contact?	*N* (%)	9 (35%)	24 (44%)	5 (42%)	38 (41%)	0.7047
Other contact with the health service?	*N* (%)	4 (5%)	5 (9%)	0 (0%)	9 (10%)	0.3259

Relative to days assessed in PACD.

Interestingly, the non-sensitized patients had significantly more days with asthma symptoms than the sensitized group (17.9 ± 7.8). However, in all other indices the symptom level of non-sensitized patients were similar or slightly higher (Table 5).

**Figure 1:**
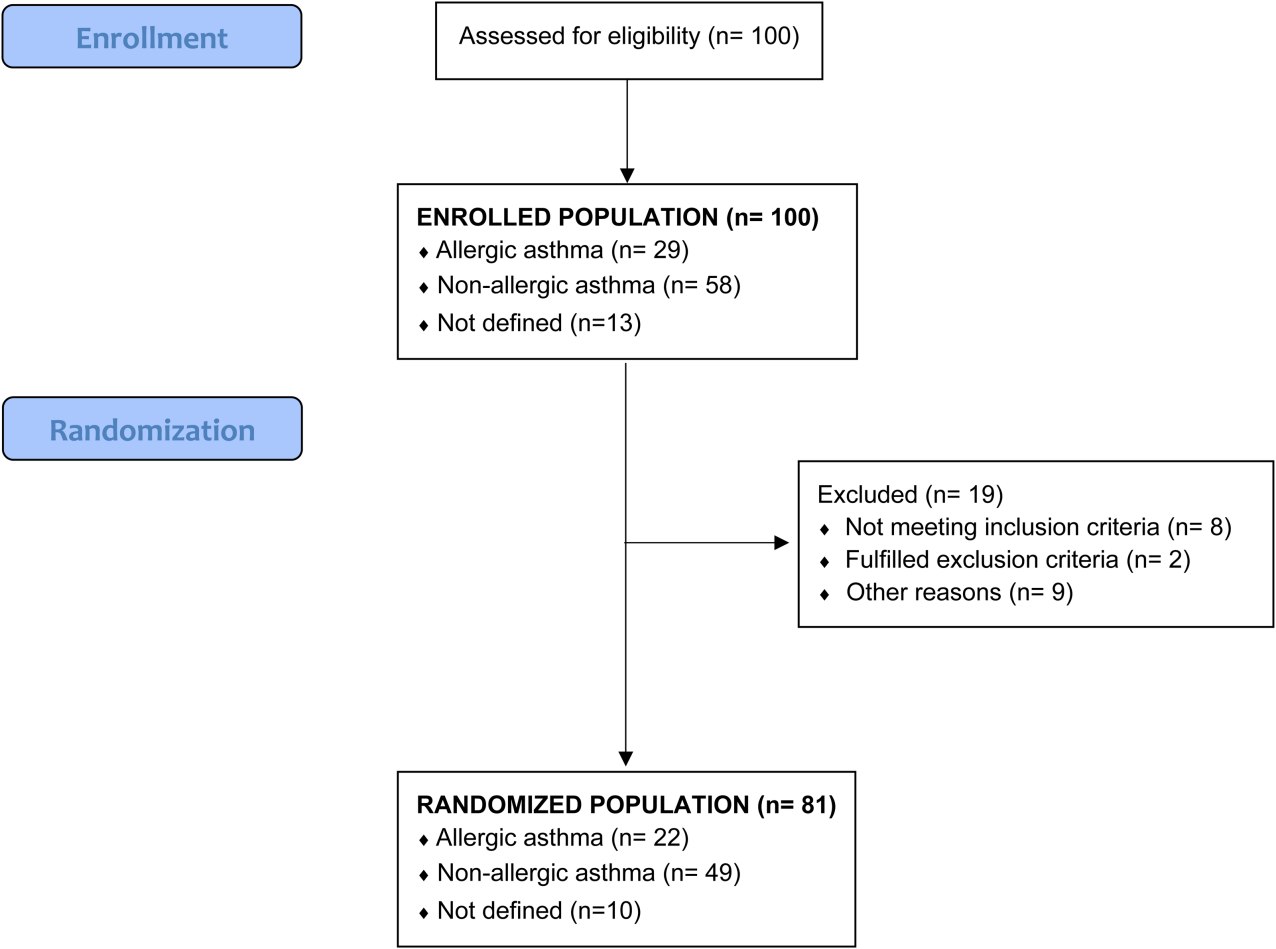
Flowchart of study patients.

Daily symptoms recording by the PACD revealed that only 7 patients were controlled at randomization, whereas 35 were partially and 58 uncontrolled according to GINA. The GINA level in relation to the month of recruitment is shown in the supplement (Supplementary Figure 1). In addition, 10 patients were not randomized due to different reasons (Figure 1. Flow chart).

## Discussion

Optimizing asthma control and management among preschool children is an unmet need since this age group experiences disproportional morbidity and health care utilization compared to school-age children with asthma (1, 11, 26, 28). Known risk factors for exacerbations include poor asthma control, previous hospitalization, viral infection, secondhand smoking and eosinophilic inflammation. So far, only a few studies have analyzed patients' characteristics and symptoms with acute severe asthma in preschoolers. This is the strength of our study, which analyzed such an age cohort at enrollment and monitored the symptom level for 4 weeks with a new App using the PACD questionnaire until randomization.

The age distribution of the present cohort showed that most children were around 3 years old, with a significant age difference between non-sensitized and sensitized children (2.9 vs. 4.2 years). Both phenotypes experienced a high number of severe asthma exacerbations defined by systemic steroid use in the last 24 months (with and without sensitization: 4.5 vs. 5.9)—although all patients were treated with ICS and often a second controller such as LTRA or LABA for more than 12 months. This is consistent with earlier reports showing that ICS and ICS + LABA combinations are less effective in this age group (27, 35). The reasons for the frequent hospitalizations and symptoms in preschool age, and especially in the non-sensitized younger patients, are manifold:

One of the main reasons are the “small airways”, or the narrowness of the still-growing bronchial system (12, 13, 36). Swelling of the mucous membranes by viral and bacterial pathogens can quickly lead to obstruction with increased airway resistance and oxygen requirements. In addition, uncontrolled asthma is associated with small airways dysfunction, bronchial hyperresponsiveness and inflammatory changes including increased airway smooth muscle mass, and eosinophilic inflammation (37–39). On the other hand, young children have to build up their immunity in infancy, when they come into contact with other children and multiple pathogens (40). Accordingly, immunizations to viral and bacterial antigens are important to prevent future exacerbations in children with asthma (41, 42).

The dogma that preschool asthma is often described as susceptible to exacerbations with relatively limited impairment is at least wrong for our cohort. The PACD dairy revealed that the percentage of days with asthma were 37,9%, and more than 38 patients (41%) had health care contacts with their local physicians in addition to the care they received in the TIPP study. The symptom burden and the healthcare utilization are much higher than in the famous study of Guilbert TW (43). In this study, the proportion of episode-free days at beginning of the ICS treatment was 72.6% only (43).

Interestingly, patients without sensitization had significantly more days with asthma, more days with salbutamol use, night-time awakenings and health care utilization than patients with allergic sensitization. At first glance this finding is surprising, since most long-term or birth cohort studies on asthma persistence in childhood showed that early sensitization—especially to HDM—is a strong indicator for asthma persistence later in life, whereas the non-sensitized patients have a greater chance to outgrow of this condition (32, 44). However, in real life, the non-sensitized preschoolers are often less well controlled by ICS and suffer from severe virus induced exacerbations, despite treatment. Several reasons could explain our findings. The non-sensitized patients were younger and smaller. Accordingly, they have increased bronchial vulnerability due to small airways and their immune system is less mature than in sensitized patients. Recently, we demonstrated that severe bronchial hyperresponsiveness (BHR) was present in both non-sensitized and sensitized preschool asthma patients (45). At follow-up, in patients without sensitization, BHR normalizes, whereas in sensitized patients HDM allergy indicates persistence of BHR and asthma beyond school-age (45).

The presence of sensitization, measured by specific IgE to common allergens, especially HDM, is one of the strongest associations for persistent childhood asthma (46, 47). In our cohort, 33% of tested patients had at least one sIgE ≥ 0.75 KU/L to the allergen tested. The sensitization pattern showed that pollen allergen (birch, grass) and HDM are the main allergens for preschool children. Surprisingly, peanut sensitization was the main allergen for food allergens and the prevalence was much higher than in the Unites states. The high prevalence of peanut sensitization may be in part due to cross-reactivity to pollen (48) and it is well known that the incidence of food allergy in asthma is higher than in the normal population (49). Among pollen allergens, birch sensitization was found in 63% and grass sensitization in 32% of sensitized patients. HDM sensitization was present in 14 patients (52%) and cat sensitization in 11 (38%) patients of sensitized children. It is well-known that early sensitization to pollen, HDM, and cat, is an indicator of a TH-2 high phenotype (high blood eosinophilia and atopy); and that these patients were more likely to develop persistent asthma at school-age (44, 46–51). Moreover, asthma patients showing a TH-2 phenotype with high total, specific IgE and elevated eosinophils are more likely to respond to ICS than the non-TH-2 phenotype (11, 28).

Our study has several limitations: First, a complete data set with eosinophils and IgE results was only available in 87% of patients. This was mostly due to problems in shipping the material and sometimes to difficulties in successfully taking blood samples from young children.

Second, it is very difficult to estimate how often such severe preschool asthma occurs in Germany. According to the recent German “Weißbuch Lunge 2023” using insurance data, at least 193,186 patients were classified as having asthma in the age group 1–4 years (52). We estimate that at least 5% of these patients suffer from partial and uncontrolled preschool asthma. Our experience with recruiting patients for the TIPP study has taught us that only 10–20 percent of parents caring for children with severe asthma have given their consent to participate in this study. These parents were mostly employed in the medical field and were less hesitant to take part in study.

Conclusion: Our data showed that a significant number of preschool children with severe asthma are not fully controlled by ICS. This is consistent with earlier reports reporting that ICS and ICS + LABA combinations are less effective in this age group. The symptom burden of this cohort was high, especially in the non-sensitized younger patients. Thus, new treatment options are urgently needed. Inhaled anticholinergic agents are a new option which, in addition to inhaled beta2-agonists, reduce the number of hospital admissions in this age group (53). At least two studies showed that Tiotropium was well-tolerated and efficacious as add-on therapy to ICS plus one or more controller medications (30, 31). However, further prospective studies such as the ongoing TIPP study (study protocol in the Supplementary Material) are required before recommending Tiotropium as additional controller therapy in severe preschool asthma.

## Data Availability

The raw data supporting the conclusions of this article will be made available by the authors, without undue reservation.

## Supplementary material

Supplementary File 1 Protocol of the TIPP study.

Supplementary Figure S1 GINA level in relation to months of enrollment.
