# Supplementary material for: Characteristics of children with severe preschool asthma prior to starting the TIPP study
Source: Front Pediatr. 2025 Mar 5;13:1558256. doi: 10.3389/fped.2025.1558256 (PMC11921963; doi:10.3389/fped.2025.1558256)
Supplement: Supplementary File 1 — Protocol of the TIPP study. [file Datasheet1.pdf]

CLINICAL TRIAL PROTOCOL

A prospective multicenter placebo-controlled trial to study the efficacy and safety of Tiotropium in preventing severe asthma exacerbations in partial and uncontrolled preschool asthma.

TIPP-Study

EudraCT	2021-000190-81
Sponsor trial code	TIPP
Draft/final	Final Version
Version	4.0
Date	05.09.2023

Sponsor

Goethe University Frankfurt
Represented by the president of the university:
[redacted]
Senckenberganlage 31
D-60325 Frankfurt am Main

Coordinating investigator (multicenter)

Department/ institution	Department for Children and Adolescents<br>Division of Allergology, Pulmonology and Cystic Fibrosis<br>Goethe University Frankfurt
Name	[redacted]
Address	Theodor-Stern-Kai 7,<br>60590 Frankfurt am Main
Phone	[redacted]
Fax	[redacted]
email	[redacted]

Operating institution of Sponsor

Department/ institution	Department for Children and Adolescents<br>Division of Allergology, Pulmonology and Cystic Fibrosis

represented by the director

Name	[redacted]
Address	Theodor-Stern-Kai 7, 60590 Frankfurt
Phone	[redacted]
Fax	[redacted]
email	[redacted]

Biometrician

Department/ institution	Interdisciplinary Center for Clinical Trials (IZKS), University Medical Center of the Johannes Gutenberg-University Mainz
Name	[redacted]
Address	Langenbeckstraße 1, 55131 Mainz
Phone	[redacted]
Fax	[redacted]
email	[redacted]

This protocol is confidential information and intended solely for the guidance of the clinical trial. It must not be disclosed to third parties not associated with the clinical trial or used for any other purpose without the prior written consent of the sponsor.

TABLE OF CONTENTS

LIST OF ABBREVIATIONS 5
SYNOPSIS 7
TRIAL SCHEDULE 9
1 INTRODUCTION 11
1.1 Scientific background 11
1.2 Trial rationale 12
1.3 Treatments and rationale for dose selection 14
1.3.1 Mode and scheme of intervention 14
1.4 Risk-benefit assessment 14
2 TRIAL OBJECTIVES 15
2.1 Primary objective 15
2.2 Secondary objectives 15
3 TRIAL DESIGN 15
3.1 Trial duration and schedule 16
3.2 Number of patients and trial centers 16
3.3 Selection of trial population 16
3.3.1 Main diagnosis for study entry 17
3.4 Primary endpoint 17
3.5 Measures taken to minimize/avoid bias 18
3.5.1 Randomization 18
3.5.2 Blinding/Unblinding 18
3.6 Selection and withdrawal of patients 18
3.6.1 Recruitment 18
3.6.2 Inclusion criteria 19
3.6.3 Exclusion criteria 19
3.6.4 Withdrawal criteria 20
3.6.5 Premature closure of the clinical trial 21
4 TRIAL TREATMENTS 21
4.1 Investigational treatments 21
4.1.1 General information about investigational medicinal product (IMP) 21
4.1.2 Therapeutic indications of Tiotropium 21
4.1.3 Therapeutic effects of Tiotropium 22
4.1.4 Known side effects 23
4.1.5 Interaction with other medicinal products and other forms of interaction 23
4.1.6 Pharmacodynamic properties 23
4.1.7 Pharmacokinetic properties 24
4.1.8 Dosage schedule 24
4.1.9 Overdose instructions 25
4.1.10 Treatment assignment 25
4.1.11 Treatment after the end of the trial 25
4.1.12 Packaging and labeling 25
4.1.13 Drug storage, supplies and accountability 25
4.1.14 Safety instructions 26
4.1.15 Procedures for monitoring patient compliance 26
4.2 Not permitted medication 26
4.3 Rescue medication or emergency treatment 28
5 TRIAL SCHEDULE 29
6 TRIAL METHODS 34
6.1 Assessment of efficacy 34
6.2 Physical Examination 35
6.3 Other assessments 38

6.3.1 Previous and intercurrent illnesses 38
6.3.2 Previous and intercurrent medical treatments 38
6.3.3 Laboratory parameters and vital signs 38
6.4 Epigenetic regulation 38
6.4.1 miRNAs in Asthma 39
7 SAFETY 40
7.1 Definitions 40
7.1.1 Adverse event (AE) 40
7.1.2 Serious adverse event (SAE) 41
7.1.3 Clarification of the difference in meaning between "serious" and "severe": 42
7.1.4 Suspected unexpected serious adverse reaction (SUSAR) 42
7.1.5 Onset and end date of AEs and SAEs 42
7.2 Assessment of AEs by investigator 42
7.2.1 Intensity/Severity 42
7.2.2 Causal relation to trial medication/procedures 42
7.3 Period of observation 43
7.4 Documentation of AEs and follow-up 43
7.5 Immediate reporting of SAEs by investigator 44
7.6 Safety evaluation and reporting by sponsor 44
7.7 Documentation of special situations 45
7.8 Emergency procedures 45
8 STATISTICS 46
8.1 Sample size 46
8.2 Analysis populations 46
8.3 Efficacy analyses 46
8.3.1 Definition and analysis of primary endpoint 48
8.3.2 Analysis of secondary endpoints 48
8.3.3 Analysis of subgroups 48
8.3.4 Interim analyses 49
8.4 Analysis of AEs 49
8.5 Analysis of clinical laboratory findings 49
9 QUALITY CONTROL, QUALITY ASSURANCE AND RISK MANAGEMENT 49
9.1 Requirements for Investigator, investigational sites and members of the investigating staff 49
9.2 Quality of the source data 50
9.3 Direct access to source data/documents 50
9.4 Monitoring 50
9.5 Risk Management 51
9.5.1 Potential COVID-19 related risks and mitigation strategies 51
9.5.2 Further mitigation strategies for potential COVID-19 related risks 51
9.6 Measures to secure compliance 52
9.7 Inspection by authorities 52
9.8 Audits 52
10 DATA MANAGEMENT 53
10.1 Responsibilities 53
10.2 Data collection 53
10.3 Data handling 54
10.4 Storage and archiving of data 54
11 ETHICAL AND LEGAL ASPECTS 54
11.1 Good clinical practice 54
11.2 Patient information and informed consent 55
11.3 Confidentiality 56
11.4 Responsibilities of the investigator 56
11.5 Approval of trial protocol and amendments 57

11.5.1 Submissions 57
11.5.2 Amendments of protocol 57
11.6 Other information to ethics committees and competent authorities 57
11.7 Documentation of Correspondence 57
11.8 Data monitoring committee (DMC) 57
11.9 Insurance 57
11.10 Agreements 58
11.10.1 Financing of the trial 58
11.10.2 Report 58
11.10.3 Publication policy 58
12 SIGNATURES 59
13 DECLARATION OF INVESTIGATOR 60
14 REFERENCES 61
15 APPENDICES 65
15.1 Additional information regarding inclusion/exclusion criteria 65
15.2 Dosing table systemic corticosteroids 65
15.3 Instructions for the use of the Respimat® inhaler 65
15.4 Instructions for the use of the Respimat® inhaler with spacer 70
15.5 Pediatric asthma caregiver diary (PACD) and instructions 74

LIST OF ABBREVIATIONS

AE	Adverse event
AMG	German Drug Law (Arzneimittelgesetz)
AP	Alkaline Phosphatase
APP	Application
ATS	American Thoracic Society
AUC	Area under the curve
BAC	Benzalkonium chloride
BfArM	Federal Institute for Drugs and Medical Devices (German: Bundesinstitut für Arzneimittel und Medizinprodukte)
BMBF	Federal Ministry of Education and Research (German: Bundesministerium für Bildung und Forschung)
CGI-C	Caregiver’s Global Impression of Change
COPD	Chronic Obstructive Pulmonary Disease
(e)CRF	(electronic) Case Report Form
CV	Curriculum vitae
COVID-19	Corona Virus Disease 2019
DMC	Data Monitoring Committee
DMP	Data Management Plan
DSMB	Data Safety Monitoring Board
DSUR	Development Safety Update Report
DVP	Data Validation Plan
EC/IEC	Ethics Committee/Independent Ethics Committee
EDTA	Ethylenediaminetetraacetic acid
EudraCT	European Clinical Trials Database
EMA	European Medicines Agency
EMLA	Eutectic Mixture of Local Anesthetics
ERS	European Respiratory Society
FEV1	Forced Expiratory Volume (in one second)
FEV1/FVC	Tiffenau-Index
FPI	First-Patient-In
FVC	Forced Vital Capacity
GCP	Good Clinical Practice
GINA	Global Initiative for Asthma
GOT	Glutamate-Oxalate-Transaminase
GPT	Glutamate-Pyruvate-Transaminase
Hz	Herz
IB	Investigator’s Brochure
ICH	International Conference on Harmonization
ICS	Inhaled Corticosteroid(s) (Solution)
IOS	Impulse oscillometry
IgE	Immunoglobulin E
IMP	Investigational Medicinal Product
INN	International Nonproprietary Name
IOS	Impulse Oscillometry
IRB	Institutional Review Board
ISRCTN	International Standard Randomized Controlled Trial Number

ISF	Investigator Site File
ITT	Intention-To-Treat
IVRS/IWRS	Interactive Voice/Web Response System
IZKS	Interdisciplinary Center for Clinical Trials
kPa*s	Kilo Pascal per second
LABA	Long-Acting Beta-Agonists
LAMA	Long-Acting Muscarin Receptor Antagonist
LKP	Leiter der klinischen Prüfung (coordinating investigator)
LPI	Last-Patient-In
LPO	Last-Patient-Out
LTRA	Leukotriene Receptor Antagonist
MedDRA	Medical Dictionary for Regulatory Activities Terminology
PACD	Pediatric Asthma Caregivers Diary
PEF	Peak Expiratory Flow
PFT	Pulmonary Function Test
pMDI	Pressurized Metered Dose Inhalers
PCR	Polymerase chain reaction
PRN	pro re nata, “as needed”
PWP	Prentice Williams and Peterson
RABA	Rapid acting ß2-agonist
RDE	Remote Data Entry
Rrs	Resistance
Rrs5	Resistance at 5 Hz
Rrs10	Resistance at 20 Hz
SAE	Serious Adverse Event
SAP	Statistical Analysis Plan
SARS-CoV-2	Severe acute respiratory syndrome coronavirus 2
SAS	Statistical Analysis Software
SDV	Source Data Verification
SOP	Standard Operating Procedure
SUSAR	Suspected Unexpected Serious Adverse Reaction
TMF	Trial Master File
TRACK	Test for respiratory and asthma control in kids
VC	Vital capacity
Xrs	Reactance
Xrs5	Reactance at 5 Hz

SYNOPSIS

**TITLE OF STUDY**	A prospective multicenter placebo-controlled trial to study the efficacy and safety of Tiotropium in preventing severe asthma exacerbations in partial and uncontrolled preschool asthma (TIPP-Study).
**SHORT TITLE**	**Ti**otropium in **p**reventing **p**reschool asthma **(TIPP)**
**EudraCT**	2021-000190-81
**SPONSOR TRIAL CODE**	TIPP
**INDICATION**	Preschool asthma
**PHASE**	III
**TREATMENTS**	Experimental intervention: Tiotropium 2.5 µg once daily (2 x 1.25 µg; 12 months treatment) via Respimat® inhaler added to ICS (Fluticasone 2 x 50 µg) Control intervention: Placebo Inhaler
**PRIMARY OBJECTIVE**	The primary objective is to evaluate whether addition of Tiotropium via Respimat® to ICS prevents severe exacerbations.
**SECONDARY OBJECTIVE**	The secondary objective is to evaluate whether addition of Tiotropium via Respimat® to ICS reduces health utilization (hospitalizations, physician visits, antibiotic use) in partial or uncontrolled preschool asthma.
**TRIAL DESIGN**	Prospective, randomized, double-blind, placebo-controlled
**TRIAL POPULATION**	Main inclusion criteria:<br>1. Male or female patients with preschool asthma aged between 1 and 5 years (< 6 at visit 1).<br>2. Physician diagnosed asthma of at least 6 months' history of asthma symptoms, including (but not limited to) wheezing, cough, and/or shortness of breath.<br>3. All patients must have been on maintenance treatment with an ICS at a stable dose, either as mono treatment or in combination with another controller medication, for at least 4 weeks before visit 1.<br>4. Patient was hospitalized due to acute severe asthma and/or was treated with at least 2 courses of systemic steroids (three days of oral steroids or one day of rectal prednisolone 100mg (Rectodelt®)) in the last 24 months before visit 1.<br>5. All patients must be symptomatic (partly controlled or uncontrolled) as defined by the GINA guideline for children aged 5 years and younger in the four weeks prior to screening (visit 1) and randomization (visit 2) despite treatment with ICS (visit 1 and visit 2).<br>6. Patients must be able to inhale from the Respimat® inhaler (with a spacer).<br>Main exclusion criteria:<br>1. Patients with a significant disease other than asthma such as, but not limited to, the following diagnoses: cystic fibrosis, bronchopulmonary dysplasia, primary immunodeficiency, congenital heart disease, parasitic disease, and foreign body aspiration.<br>2. Patients with clinically relevant abnormal screening hematology or blood chemistry will be excluded if the abnormality defines a significant disease as defined in exclusion criterion.<br>3. Patients with known hypersensitivity to anticholinergic drugs, or any other components of the Tiotropium inhalation solution.<br>4. Patients with any severe acute asthma exacerbation or severe respiratory tract infection defined by systemic steroid intake or hospitalization in the four weeks prior to visit 1.
**TRIAL DURATION AND DATES**	Recruitment period: 24 months<br>First patient in: 02/2022<br>Duration of the trial: 12 months<br>Last-patient-out: 02/2025 (planned)
**NUMBER OF PATIENTS**	It is planned to enroll 152 patients

**NUMBER OF SITES**	n=13
**PRIMARY ENDPOINT**	Primary efficacy endpoint:<br>Time to first severe exacerbations, defined by hospitalization and/or at least 3 days of systemic steroids or one day of rectal prednisolone (Rectodelt®).
**SECONDARY ENDPOINTS**	Key secondary endpoint(s): number of severe exacerbations, number of hospitalizations (severe exacerbation), (severe) exacerbation-free survival time, number of asthma-related events defined by use of antibiotics, percentage of night-time awakenings due to asthma symptoms as assessed by the electronic patients diary/PACD, percentage of days without asthma symptoms assessed by TIPP diary App (PACD), , percentage of days and episodes of > 3 days with use of PRN salbutamol rescue medication, health utilization (number of physician visits), number of missed days in daycare, result of TRACK, CGI-C at end of treatment and potential biomarkers for treatment response:<br>Eosinophils, IgE, specific IgE to common allergens (birch, grass, *Dermatophagoides pteronyssinus*, *Alternaria*, *Cladosporium,* cat, dog, hen's egg, cow's milk and peanut.<br>Assessment of safety:<br>Safety will be ensured by analysis of symptom scores and rescue medication use by the electronic diary, and adverse events (AEs).
**STATISTICAL ANALYSIS**	Efficacy:<br>The comparison between groups will be made by time to first severe exacerbation within 12 months of therapy.<br>Description of the primary efficacy analysis and population:<br>The time to first severe exacerbation will be analyzed by Cox regression with factor treatment and the covariates age group and center. Analyses will be performed in the intention-to-treat population and repeated in the per-protocol population as sensitivity analyses. Treatment differences will be displayed by hazard ratios and 95% confidence intervals.<br>Safety: For safety analyses, frequencies of patients experiencing at least one adverse event (AE) will be displayed by system organ class and preferred term according to MedDRA terminology.<br>Secondary endpoints:<br>For secondary analyses, Cox regression for time to event data will be used and negative binomial regression for count of events. Further secondary endpoints will be assessed by chi-square or Fisher's exact test for categorical variables or t-test or Mann-Whitney-U-test for continuous variables.<br>All tests will be conducted on a two-sided significance level of alpha = 0.05.

TRIAL SCHEDULE

Action / Visits	V1 Screening[10]	Visit 2[11, 12]	Visit 3	Visit 4	TC 4a[9] ☎	Visit 5	TC 5a[9] ☎	Visit 6	TC 6a[9] ☎	Visit 7 (End of study)
Trial day / week	-6 to -4 weeks	0	6 ± 1 week	16 ± 1 week	22 ± 1 week	28 ± 1 week	34 ± 1 week	40 ± 1 week	46 ± 1 week	52 ± 2 weeks
Patient information and informed consent	×									
Demographics (e.g. sex, age, race)	×									
Previous and concomitant diseases	×	×	×	×	×	×	×	×	×	×
Previous and concomitant treatments	×	×	×	×	×	×	×	×	×	×
Inclusion/exclusion criteria	×	×								
Physical examination	×	×	×	×		×		×		×
Vital signs (BP, pulse, temperature)	×	×	×	×		×		×		×
Height and weight	×	×	×	×		×		×		×
Laboratory testing[1]	×									×
Spirometry/Oscillometry in patients ≧ 4 years pre-dosing[2,3]	×	×	×	×		×				×
Spirometry/Oscillometry pre and 30 minutes post-dosing[2,4]		×								
Randomization		×								
Training in use of Respimat® (with spacer)		×								
Check study medication compliance			×	×	×	×	×	×	×	×
Dispense study medication[5]		×	×	×		×		×		
Dispense/Check rescue medication	×	×	×	×		×		×		
Collect study medication / Drug accountability		×	×	×		×		×		×
Instructions for using the TIPP diary App (PACD[6])	×									
Review/download TIPP diary App (PACD[6])		×	×	×	×	×	×	×	×	×
TRACK test	×			×						×
Caregiver's global impression of change (CGI-C)[7]										×
Assessment of asthma control (GINA)[8]	×	×		×		×				×
Adverse events (AE)	×	×	×	×	×	×	×	×	×	×
Concomitant therapy	×	×	×	×	×	×	×	×	×	×
Trial completion										×

1. Hematology and blood chemistry. Topical (percutaneous) analgesia to reduce pain associated with venipuncture, e.g. amethocaine gel or EMLA cream (Eutectic Mixture of Local Anesthetics), must be applied (may only be omitted in patients who explicitly refuse it). A maximum of 3 puncture attempts to obtain blood (approx. 5 mL) will be performed. Visit 1 laboratory parameters comprise: differential blood count, glutamate-pyruvate-transaminase (GPT), glutamate-oxalate-transaminase (GOT), alkaline phosphatase (AP), urea, creatinine levels and total serum IgE and specific IgE to birch, grass, *Dermatophagoides pteronyssinus, Alternaria, Cladosporium,* cat, dog, hen´s egg, cow´s milk and peanut. All paramaters will be documented in the electronic Case Report Form (eCRF). PaxGene tubes for miRNA will only be sampled at V1.
2. Spirometry/ Oscillometry will only be performed in children ≥ 4 years who are capable of providing spirometry / oscillometry of acceptable quality. To confirm acceptable quality, spirometry / oscillometry measurements will be performed in patients ≥ 4 years if they are capable of providing measurements of acceptable quality (with a mouthpiece) and if the required equipment is available at site.
3. It is not necessary to perform lung function if the medical files provide clear evidence that the patient is not able to perform technically acceptable lung function. Lung function measurements are performed -10 minutes prior to trial drug administration and 30 minutes post-dose at visit 2 only.
4. Spirometry / oscillometry will be performed -10 minutes prior to trial drug administration and 30 minutes, post-dose at visit 2.
5. Each Respimat® inhaler contains drug supply for 30 days.
6. In order to reduce the burden of parents, the electronic diary does not have to be filled out daily all year round. It is well known from many studies that asthma exarcerbations occur particularly in autumn, winter and spring. For this reason, the diary does not have to be kept for 4 months in the months of May, June, July and August. If the patients are recruited in the summer months, they only need to keep an electronic diary between visit 1 and visit 2.
7. The CGI-C should be completed prior to other visit assessments and should precede any discussions with a health professional (physician or nurse).
8. Levels of asthma control will be measured as described in the GINA guideline.
9. The following data are collected during telephone contacts: compliance of study medication use, health status, AEs, concomitant diseases and treatments, concomitant therapies, and medication, and exacerbations.
10. The screening period (between visit 1 and visit 2) may be extended by 7 days (see flow chart), e.g. for administrative reasons.
11. All patients must be symptomatic (partly controlled or uncontrolled) as defined by the GINA guideline for children aged 5 years and younger (7) at visit 1 (screening) and before randomization at visit 2. If this criterion is not met at visit 2, the visit can be repeated once within 2 weeks.
12. In case of severe asthma exacerbation or severe respiratory tract infection (or any other acute illnesses) defined by systemic steroid intake or hospitalization in the 4–6 weeks prior to visit 2, the randomization visit (visit 2) must be postponed until 4 weeks following recovery from the infection or exacerbation.

1 INTRODUCTION

1.1 Scientific background

Asthma is the most common chronic disease in children, imposing a high lifetime burden on individuals, their caregivers, and healthcare systems (1, 2). The prevalence of childhood asthma has increased over the last 20 years, most likely due to a greater awareness of this condition and changes in diagnostic practice. In Germany, parent-reported asthma prevalence in preschool children (< 6 years) ranged from 2.6 to 2.8% (3).

As in adults, a considerable proportion of asthma in children is not well controlled by inhaled corticosteroids (ICS), which represents a significant healthcare concern (4). Failure to control asthma has a negative impact on patients' quality of life, and increases the risk of future exacerbations, with associated increased requirement for healthcare utilization and costs (5, 6).

The global prevalence of diagnosed current asthma in children 5 years and younger cannot be estimated well due to the lack of international consensus on the diagnostic criteria. Asthma is, however, the most common chronic disease of childhood with a significant impact on the pediatric health care resource utilization for asthma management by children below 5 years of age despite current treatment (7, 8). Asthma typically begins in early childhood and is generally diagnosed more often in boys than girls (8, 9). Atopy is present in the majority of children with asthma over the age of 3, and allergen-specific sensitization is one of the most important risk factors for the development of asthma (10).

Of note is the diagnostic uncertainty among young children in whom wheezing is more likely to be associated with lower respiratory tract infection, and often is transient (9, 11–13). Although the asthma prevalence of school children is higher than in toddlers, severe asthma exacerbations with emergency visits and hospital admissions are disproportional more often in preschool asthma (14–16). In addition, young children have increased vulnerability to adverse outcomes due to small airways and possibly increased bronchial airway reactivity, in comparison with older children (17). Respiratory distress in the setting of infection can rapidly become life-threatening. For this reason, the relatively high utilization of emergency visits and hospital admissions in preschoolers may be well explained. A retrospective analysis of the electronic medical records at the Department for Children and Adolescents, Goethe University showed a significant number of acute asthma admissions (approximately 60 new cases annually) in this age group, 1–5 years. In addition, re-admissions with acute asthma exacerbations were surprisingly high with approximately 20%, despite frequent hospital discharge with asthma control therapy like inhaled corticosteroids (ICS) and leukotriene receptor antagonist (LTRAs) (18). This is much higher than in a study in England showing 5% and more close to the range of a recent report in France with 15% re-admission rates (18–20). Early intervention with anti-inflammatory agents is indicated in preschool asthma to prevent the development of uncontrolled asthma and frequent exacerbations with high health care utilization (16, 21, 22). Although studies are limited, data suggest that an asthma-like inflammation (presence of eosinophils and allergic sensitization) may be present at a very early age in some children with recurrent wheeze (16, 23). Currently available anti-inflammatory treatments for first-line therapy in preschool asthma include ICS and LTRAs. Both ICS and LTRAs have been found to be effective in this age group (24–27). Sensitization and eosinophils are useful in the prediction of future exacerbation and may identify children most

likely to respond favorably to daily ICS treatment (16, 23, 27). ICS is effective to reduce severe exacerbations by only 36% in preschool asthma patients (16, 27). Thus, asthma exacerbations occur frequently despite the regular use of ICS. Optimizing asthma management among preschool children by quintupling the ICS dose at the early signs of loss of asthma control did not reduce the rate of severe asthma exacerbations and may be associated with diminished linear growth (28). In addition, in children with persistent asthma, the addition of long-acting beta-agonists (LABA) to ICS was not associated with a significant reduction in exacerbations requiring systemic steroids (29, 30).

Currently, short-acting Ipratropium is a widely used treatment of patients with preschool asthma as a reliever in acute wheezing. According to the results of Beck R, et al. (31), a beneficial effect of the short-acting anticholinergic Ipratropium as inhalation added to the standard care could be shown. As Tiotropium offers a superior time-response profile as a bronchodilator to Ipratropium, it is likely to be more effective and has sustained anti-obstructive effects for 24 hours in children 1–5 years with asthma. The 24-hour duration of action profile may be of special value in toddlers suffering from nocturnal events of *e.g.,* shortness of breath, which is the case in moderate and severe but still not optimally controlled asthma. The ease of use taking a drug only once daily could also enhance compliance of the younger patient population.

Tiotropium in the Respimat® inhaler is the first long-acting muscarinic antagonist licensed for treatment of asthma and effective as add-on therapy to ICS in patients aged 6 years and older with severe asthma who experienced one or more severe asthma exacerbations in the preceding year. Tiotropium was found to be well-tolerated and efficacious add-on to ICS plus one or more controller medications in many clinical trials in children (32–36). Its therapeutic success in clinical trials resulted in Food and Drug Administration and the European Medicines Agency (EMA) approval for asthma treatment in people aged ≥ 6 years in the US and EU. GINA strategy includes the addition of long-acting muscarin receptor antagonist (LAMA) at Step 4–5 as an add-on to ICS + LABA treatment for patients aged ≥ 6 years, before escalation to treatment with biologics or low-dose oral corticosteroids (37). Interestingly, Tiotropium might also be relevant as an asthma control therapy, since there is preliminary evidence that it not only functions as a bronchodilator through inhibition at the muscarinic M3 receptors but also has anti-inflammatory properties (38). In addition, Tiotropium has been shown to be efficacious regardless of IgE levels and blood eosinophils and might therefore be especially interesting in preschool asthma (16, 39, 40) since the percentage of preschool children with asthma with elevated IgE levels and/or significant eosinophilia is lower than in school asthma. Thus, adding Tiotropium to ICS might be a new promising treatment option for severe uncontrolled preschool asthma (41, 42).

1.2 Trial rationale

Optimizing asthma management among preschool children is an unmet need since this age group experiences disproportional morbidity and health care utilization compared to school-age children with asthma (14–16). At present, there are insufficient data to recommend additional controller therapies, such as combinations of ICS with LABAs or LAMAs in this age group (37).

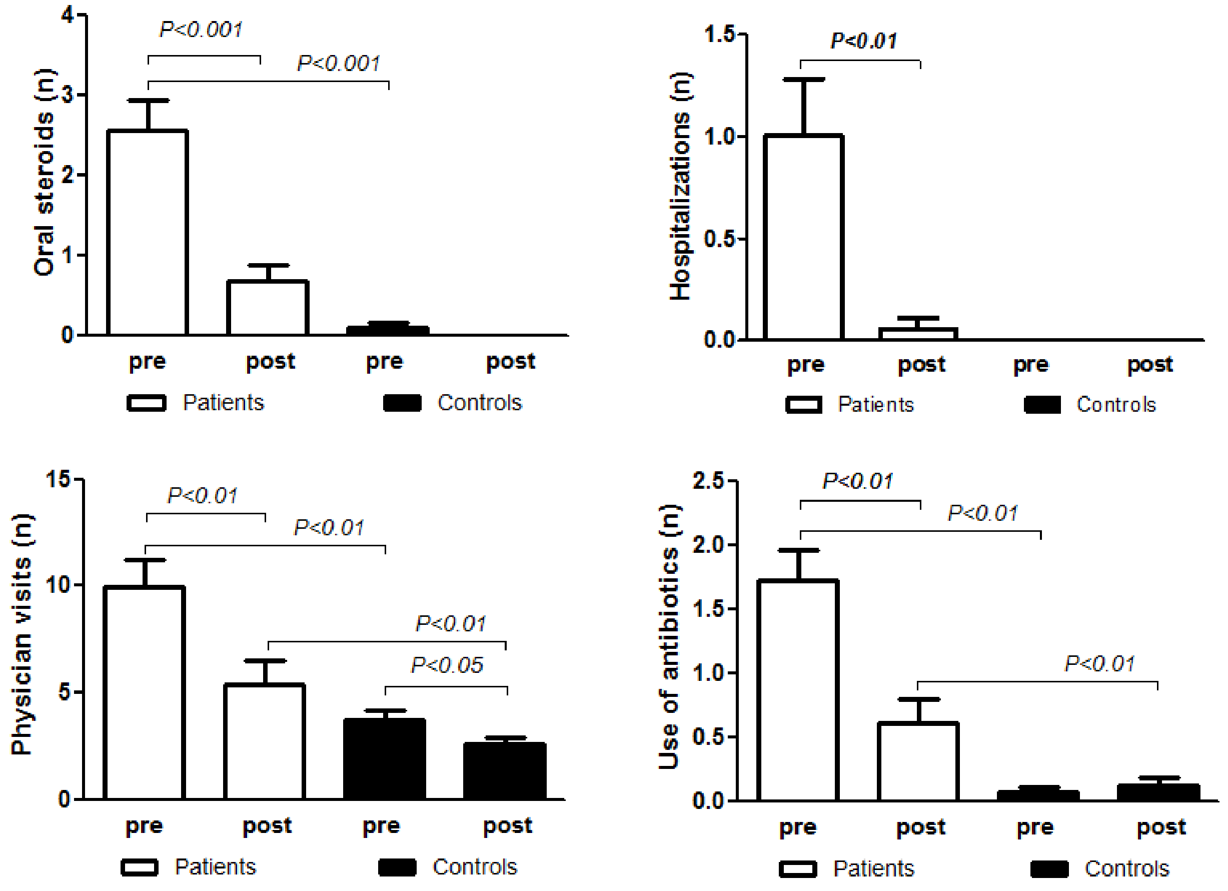

Figure 1: Tiotropium a new treatment option for severe uncontrolled preschool asthma.

Adding Tiotropium to ICS might be a new promising treatment option for severe uncontrolled preschool asthma (41, 42). A recent study in patients aged 1-5 years with preschool asthma showed that the tolerability of Tiotropium + ICS was similar to that of placebo + ICS and demonstrated the potential to reduce asthma-related events compared with placebo (42). This is in line with a recent study of our group. A retrospective analysis of electronic outpatient medical records (Frankfurt University Hospital) was conducted from 2017–2019 to screen for children with persistent asthma treated with ICS + LABA and switched to Tiotropium as add-on treatment, due to severe uncontrolled asthma. 21 children with uncontrolled preschool asthma were compared with 42 children without asthma matched for age, sex, and screening date. There was a significant reduction of hospital admissions, steroid courses, and physician visits when Tiotropium was added to the current medium-dose ICS + LABA treatment (Figure1; Zielen et al. (43).

However, the number of patients included in both studies was small, and further research is required to confirm the beneficial effect on exacerbations in this age group. Additional well-powered trials are needed and called for to further assess the safety and efficacy of Tiotropium in young children before it can be implemented in the current guidelines and disease management plan. Thus assessing its use for treatment in uncontrolled preschool asthma, in whom alternative controller treatments are limited, could be extremely valuable due to the high unmet medical need in this age group (16, 37).

1.3 Treatments and rationale for dose selection

A total of 1691 children aged 1–17 years, (560 patients received Tiotropium 5 µg; 559 patients received Tiotropium 2.5 µg; 572 patients received placebo) were treated in five pediatric studies so far (36). Drug-related AEs, those leading to discontinuation and SAEs were very low and balanced between treatment groups, irrespective of age, disease severity, or gender. Interestingly, the FDA in the US licensed 2.5 µg whereas the EMA licensed 5.0 µg Tiotropium for use in patients with asthma. A recent PubMed search including phase 2 and 3 randomized controlled trials (RCTs) evaluated the effects of 2.5 µg Tiotropium on lung function parameters in patients with asthma. The authors found the following: Based on the results of phase 2 and 3 studies 2.5 µg Tiotropium as an add-on to ICS therapy was safe and associated with consistent improvements in lung function in patients with asthma of varying severities across different age groups (44). Accordingly, in the planned study we will use the lower dose of 2.5 µg Tiotropium to be effective and to save cost for better patient care.

1.3.1 Mode and scheme of intervention

Administration of Tiotropium inhalation solution is achieved with the Respimat® inhaler in combination with a drug reservoir/cartridge. All children will use a spacer to inhale Tiotropium Respimat®. No special device will be provided. However, in children < 4 years connected with a face mask and > 4 years connected with a mouth piece. We recently established the age at which children below 5 years are able to handle the Respimat® inhaler and to what degree help may be appropriate (45). A further objective of this study was to acquire inhalation flow profiles depending on the age of the children in the respective "successful handling configuration" (46). The vast majority of children and parents/caregivers were satisfied with operation, instructions for use, handling, and ease of holding the Respimat® inhaler. Although the majority of children aged 4 years could handle the Respimat® inhaler without a spacer, the recommendation is that children below 5 years should use the Respimat® inhaler with a spacer.

1.4 Risk-benefit assessment

The favorable benefit-risk ratio based on the so far acquired knowledge about inhaled Tiotropium is the rationale to conduct the study with Tiotropium in preschool asthma. Clinical data in all finished proof-of-concept trials in patients with persistent asthma showed superiority of Tiotropium over placebo treatment with respect to several lung function parameters and also non-inferiority to salmeterol with respect to several lung function tests (47). The same positive add-on effect of Tiotropium on lung function and asthma control was demonstrated in children 6–18 years with moderate and severe asthma (32–35). Accordingly, Tiotropium was licensed as add to ICS in children $\geq$ 6 years addition with severe uncontrolled asthma in Germany 2018. In addition, Tiotropium was safe in all age groups especially in young children (36, 42, 43). In children aged 1-5, a randomised, double-blind, placebo-controlled, phase II/III clinical study in a total of 101 children demonstrated that the number of asthma adverse events was lower for Spiriva Respimat compared to placebo. Exploratory efficacy evaluations did not show differences for Spiriva Respimat from placebo.

Adding Tiotropium to ICS may significantly increase asthma control in this age group. Patients will have fewer symptoms, more symptom-free days, and fewer hospitalizations. Thus, there may be a significant increase of quality of life of patients, parents, and their families.

1.5 CONTROL(S) / COMPARATOR(S)

ICSs are recommended by asthma guidelines as first-line therapy for school-age children. A recent meta-analysis confirmed the beneficial role of ICS among preschool children (7, 16, 27). Consistent with current guidelines all children hospitalized due to an asthma exacerbation will be treated at least with a medium-dose of ICS (Fluticasone 2 x 50 µg). Adding a second or third controller like Montelukast or LABA is allowed.

A placebo group is included in the trial because the absence of such a treatment arm would reduce the reliability of the trial results. In all previous trials in children no untoward serious adverse events happened to patients treated with placebo (on top of maintenance therapy) and the overall incidence of AEs and the incidence of asthma exacerbations, were similar in active treatment arms and in placebo (32–36, 42). Based on these data, and the implementation of a set of rules to withdraw patients with severe asthma deterioration, a placebo treatment group in this trial (on top of maintenance treatment with at least ICS) is considered safe.

2 TRIAL OBJECTIVES

2.1 Primary objective

The primary objective is to evaluate whether addition of Tiotropium via Respimat® to ICS prevents severe asthma exacerbations (defined by hospitalization and/or three consecutive days of oral or parenteral systemic steroid intake or one day of rectal prednisolone application (Rectodelt®).

2.2 Secondary objectives

The secondary objective is to evaluate whether addition of Tiotropium via Respimat® to ICS reduces health utilization (hospitalizations, physician visits, antibiotic use) in partial or uncontrolled preschool asthma.

3 TRIAL DESIGN

This is a prospective randomized, double-blind, placebo-controlled, parallel-group trial (a non-commercial trial, clinical phase III) to evaluate the safety and efficacy of Tiotropium inhalation solution (2.5 µg) administered once daily via Respimat® Inhaler for 52 weeks to prevent severe asthma exacerbations in patients 1–5 years with uncontrolled or partially controlled preschool asthma and at least one severe asthma exacerbation requiring hospitalization and/or was treated with 2 courses of systemic steroids in the last 24 months before visit 1 (three days of oral systemic steroids or one day of rectal prednisolone).

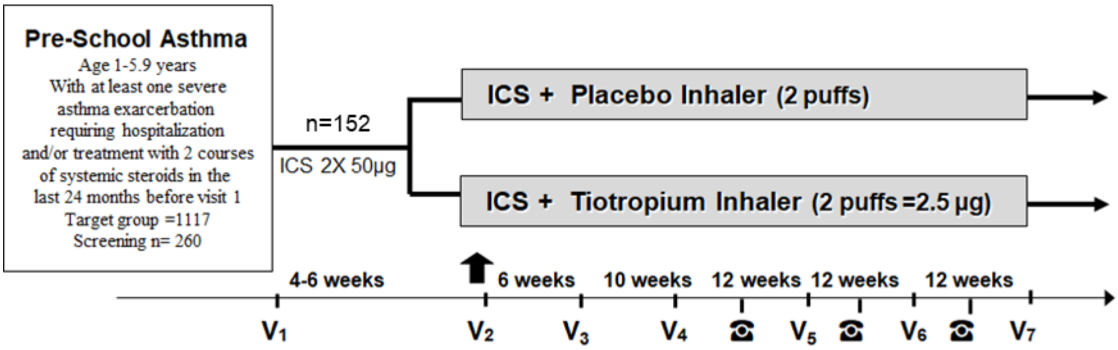

Figure 2: Trial design

After signing informed consent and completing an initial screening visit (Figure 2, V1), patients enter a 4–6 week screening period to ensure clinical stability (i.e. no severe exacerbations). Patients who meet all inclusion criteria and do not present with any of the exclusion criteria will be randomized into the 52-week treatment period (V2) in which they will be assigned at random to one of the two treatment groups. Additional clinic visits will be scheduled at 6, 16, 28, 40, and 52 weeks after start of therapy (V2). A telephone contact will be scheduled between V4 and V5, V5 and V6, and V6 and V7.

Pulmonary function tests (PFTs) will be performed in patients ≥ 4 years who are capable of providing PFTs of acceptable quality. Spirometry/Oscillometry measurements will be performed in patients ≥ 4 years if they are capable of providing measurements of acceptable quality (with a mouthpiece) and if the required equipment is available at site.

3.1 Trial duration and schedule

The duration of this trial is expected to be 36 months (24 months recruitment, 12 months treatment phase). Patient recruitment is planned to start in February 2022 and end in February 2024. The actual overall trial duration or patient recruitment period may differ from these periods.

3.2 Number of patients and trial centers

It is planned to include 152 patients (randomized) in the clinical trial, currently 13 trial sites agreed to participate.

3.3 Selection of trial population

A sufficient number of asthmatic patients aged 1–5 years of either sex will be enrolled in the study to ensure that 152 children are entered (randomized) in the trial. Recruitment is competitive. Additional sites may be initiated and 'non-productive' sites may be closed to ensure the trial timelines.

Participation in the oscillometry measurements is optional and not a prerequisite for patients to participate in the trial. The number of patients participating in the oscillometry measurements is not limited.

Every effort should be made to keep patients in the study until completion of all study procedures. Patients who discontinue after randomization may not be re-enrolled later. A record will be kept of all patients who fail to complete all study visits and their reasons for early discontinuation.

3.3.1 Main diagnosis for study entry

Outpatients aged 1–5 years with a history of at least one severe asthma exacerbation requiring hospitalization in the last 24 months and/or treated with 2 courses of systemic steroids before visit 1 and who are symptomatic (partly or uncontrolled) despite their current maintenance treatment with ICS are eligible for inclusion if they fulfill all the inclusion criteria (section 3.7.2) and none of the exclusion criteria (section 3.7.3).

3.4 Primary endpoint

The primary efficacy endpoint is the time to first severe exacerbations defined by hospitalization and/or at least 3 days of systemic steroids or one day of rectal prednisolone (Rectodelt®). Severe exacerbations were chosen as primary outcome because they are patient-important outcomes that have significant consequences for children, caregivers, and the health care system.

Secondary endpoints for all patients:

- Number of severe exacerbations
- Exacerbation-free survival time defined as the time from randomization until the first severe exacerbation.
- Percentage of patients requiring systemic corticosteroid rescue for asthma during the 52-week treatment period.
- Number of hospitalizations (severe exacerbation).
- Percentage of days and episodes of $\geq$3 days with use of PRN salbutamol rescue medication during 52-week treatment period (non-severe exacerbation).
- Percentage of days without asthma symptoms assessed by TIPP diary App (PACD).
  - A day without asthma symptoms is defined as a day during which the patient experienced no asthma symptoms (daytime and overnight; question 1, 2, 5, 6 , did not use rescue medication (salbutamol) (question 3 and 7), and had no asthma exacerbation/worsening requiring systemic corticosteroids, an unscheduled visit to a doctor's office, or hospital (question 11).
- Percentage of night-time awakenings due to asthma symptoms as assessed by the TIPP diary App.
- Health utilization defined as number of physician visits during treatment.
- Number of missed days in daycare.
- Number of asthma-related events defined by use of antibiotics.
- Evaluation of TRACK at V1, at V4 and at end of treatment.
- CGI-C at end of treatment.

Additional secondary endpoints for children aged 4 years and older:

Only applicable for patients able to perform technically acceptable (and reproducible) pulmonary function tests.

- The forced expiratory volume in one second (FEV1) and the forced vital capacity (FVC) measured 30 minutes post IMP will be compared to pre-dosing FEV1 and FVC values at visit 2.
- Trough FEV1 and through FVC response will be determined at visit 3, visit 4, visit 5 and visit 7. Trough FEV1 is defined as the FEV1 and FVC measured approximately 24 hours after the last IMP inhalation. Response is defined as a 10% improvement in FEV1 or FVC, respectively, compared to baseline (visit 2, pre-dosing).
- Potential biomarkers for treatment response (Eosinophils, IgE, specific IgE to common allergens: birch, grass, *Dermatophagoides pteronyssinus*, *Alternaria*, *Cladosporium*, cat, dog, hen's egg, cow's milk and peanut)

Assessment of safety: Safety will be ensured by analysis of symptom scores and rescue medication by electronic diary, and adverse events (AEs).

3.5 Measures taken to minimize/avoid bias

3.5.1 Randomization

A randomization list will be generated by an independent person from IZKS using a SAS® based computer program with permuted blocks without stratification factors. The randomization list assigns an identification code (medication number) to a treatment. The randomization list will be kept in safe and confidential custody at IZKS Mainz. Randomization number will be documented in the eCRF system with an identification code to a patient without knowing the treatment behind the medication number.

3.5.2 Blinding/Unblinding

In addition to the trial medication, the investigator will receive a set of sealed envelopes, comprising of one envelope for each medication number. An identical set of sealed envelopes will be held at the pharmacy of the University Medical Center Mainz. These envelopes contain information on the patients' trial medication and are to be opened only under circumstances in which it is medically imperative for diagnostic or therapeutic decisions to know which trial medication the patient is receiving. Date and reason for opening a sealed envelope must be documented by the investigator or an authorized person on the open envelope, on the eCRF, and in the medical record of the patient.

In any case, before unblinding, the investigator should consult the coordinating investigator. The randomization envelopes are not to be opened by the investigator at the end of the trial. All envelopes will be collected by the monitor of the IZKS Mainz at the end of the trial.

3.6 Selection and withdrawal of patients

3.6.1 Recruitment

Patients eligible for enrolment must meet all of the following criteria (see 3.7.2. and 3.7.3). No patient will be allowed to enroll in this trial more than once.

3.6.2 Inclusion criteria

Patients meeting all of the following criteria will be considered for admission to the trial:

1. All patients' parents (or legal guardians) must sign and date an informed consent, consistent with ICH-GCP guidelines and local legislation prior to participation in the trial. In addition, age-appropriate assent in accordance with local regulations or guidelines or both before enrollment in the study and prior to the beginning of any study-specific procedures will be obtained from the child.
2. Ability of parents/legal guardians to understand nature, importance, and individual consequences of the clinical trial.
3. Male or female patients with preschool asthma aged between 1–5 years (< 6 at visit 1).
4. Physician diagnosed asthma of at least 6 months' history of asthma symptoms, including (but not limited to) wheezing, cough, and/or shortness of breath.
5. All patients must have been on maintenance treatment with an ICS at a stable dose, either as mono treatment or in combination with another controller medication, for at least 4 weeks before visit 1.
6. Patient was hospitalized due to acute severe asthma and/or was treated with at least 2 courses of systemic steroids (three days of oral steroids or one day of rectal prednisolone 100mg (Rectodelt®) in the last 24 months before visit 1.
7. All patients must be symptomatic (partly or uncontrolled) as defined by the GINA guideline for children aged 5 years and younger in the four weeks prior to screening (visit 1) and randomization (visit 2) despite treatment with ICS.
8. Patients must be able to inhale from the Respimat® inhaler (with a spacer).

3.6.3 Exclusion criteria

Patients presenting with any of the following criteria will not be included in the trial:

1. Patients with a significant disease other than asthma such as, but not limited to, the following diagnoses: cystic fibrosis, bronchopulmonary dysplasia, primary immune-deficiency, congenital heart disease, parasitic disease, and foreign body aspiration.
2. Patients with clinically relevant abnormal screening hematology or blood chemistry will be excluded if the abnormality defines a significant disease as defined in the exclusion criterion.
3. Patients with a history of congenital or acquired heart disease, or patients who have been hospitalized for cardiac syncope or failure during the past year.
4. Medical or psychological conditions that would jeopardize an adequate and orderly completion of the trial.
5. Patients with known hypersensitivity to anticholinergic drugs, or any other components of the Tiotropium inhalation solution.
6. Patients with severe acute asthma exacerbation or severe respiratory tract infection defined as systemic steroid intake or hospitalization in the four weeks before visit 1 (screening).

7. Patients who have previously been randomized in this study or are currently participating in another study.
8. Patients with moderate to severe renal impairment as Tiotropium is a predominantly renally excreted drug
9. Patients with chronic systemic steroids use, and patients treated with monocloncal antibodies like Omalizumab and Dupilumab.

Patients can withdraw their consent without giving any reasons at all times during the trial and this should not entail any disadvantages for them. However, the investigator should try to arrange for the performance of a final visit in order to get conclusive findings of the investigation.

3.6.4 Withdrawal criteria

In general, patients may be withdrawn from the trial. The responsible investigator decides about withdrawal of patients from the clinical trial in case of occurrence of criteria mentioned below:

- At their own request or at request of the legal representative,
- At the instigation of the sponsor e.g. for safety reasons,
- If, in the investigator’s opinion, a continuation of the trial would be detrimental to the patient’s well-being,
- If the trial is stopped early due to unforeseen toxicity or inefficacy of the trial treatment becomes evident,
- If the patient does not comply with the essential requirements of the study protocol.

The investigator decides on withdrawal of patients from the clinical trial in case of occurrence of criteria mentioned above. In all cases, the reason for withdrawal must be recorded in the eCRF and in the patient’s medical records. In case of withdrawal of a patient at his own request, as far as possible the reason should be asked for and documented, the patient should be followed up, and all examinations scheduled for the final trial day should be performed and documented.

All ongoing serious AEs of withdrawn patients should be followed up until resolution or stabilization of the health condition of the patient. Generally, all (serious) AEs (related and not related to study medication) must be documented from the time the subject has signed the informed consent document until 30 days after end of study treatment; (serious) AEs with potential relationship to study medication occurring > 30 days after end of study treatment will also be documented.

Withdrawn patients will not be replaced.

Premature closure of trial sites

A trial site can be prematurely closed in case of obvious and major protocol violations, violations of legal and ethical regulations (GCP), non-compliance of investigator to adhere to protocol rules, insufficient recruitment, or upon wish of the individual site. The site may not be closed based on the decision of the coordinating investigator only; such a decision will be made by the study steering committee.

3.6.5 Premature closure of the clinical trial

For following reasons the whole trial may be discontinued at the discretion of the sponsor:

- New risks for patients become known.
- Inefficacy of the trial medication becomes evident.
- Occurrence of up-to-date unknown AEs in respect of their nature, severity, and duration or the unexpected increase in the incidence of known adverse events.
- Medical or ethical reasons negatively affecting the continued performance of the trial.
- Difficulties in the recruitment of patients.

The ethics committees (EC) and the competent authorities must be notified within 15 days. All trial material (randomization envelopes, investigational medicinal products, etc.) must be returned to the sponsor, or the pharmacy of the University Medical Center Mainz, or fetched by the monitor.

4 TRIAL TREATMENTS

4.1 Investigational treatments

4.1.1 General information about investigational medicinal product (IMP)

Investigational product

Drug code:	PZN: 3224421
International non-proprietary name (INN):	Tiotropiumbromid
Formulation:	Tiotropium Respimat® solution (1.25 microgram tiotropium, benzalkonium chloride, disodium edetate, purified water, hydrochloric acid 3.6%) for inhalation 1.25 µg/actuation
Manufacturer:	Boehringer Ingelheim Pharma GmbH & Co.KG
Dosage authorized:	2 x 1.25 µg by the FDA; 2 x 2.5 µg by the EMA

Comparative product (Placebo)

Formulation:	Placebo solution (benzalkonium chloride, disodium edetate, purified water, hydrochloric acid 3.6%) for inhalation
Manufacturer:	Boehringer Ingelheim Pharma GmbH & Co.KG

In the present study, the lower dose of 2.5 µg Tiotropium will be used to be effective and to save cost for better patient care.

4.1.2 Therapeutic indications of Tiotropium

COPD

Tiotropium is indicated as a maintenance bronchodilator treatment to relieve symptoms of patients with chronic obstructive pulmonary disease (COPD).

Asthma

Spiriva Respimat is indicated as add-on maintenance bronchodilator treatment in patients aged 6 years and older with severe asthma who experienced one or more severe asthma exacerbations in the preceding year (see sections 4.2 and 5.1).

4.1.3 Therapeutic effects of Tiotropium

Please refer to the most recent summary of product characteristics of Tiotropium Respimat® (48).

Tiotropium in the Respimat is a quaternary ammonium compound developed as a long-acting orally inhaled anticholinergic bronchodilator and approved for the maintenance treatment of asthma and chronic obstructive pulmonary disease (COPD).

Tiotropium in the Respimat® inhaler has been tested in a set of phase III clinical studies has been registered with 5 µg in several countries of the European Union (Spiriva® Respimat®) and in the 2,5 µg (2 x 1.25 µg per actuation) in the United States of America.

The beneficial effect of Tiotropium on bronchoconstriction is well established and clinically used for years in the treatment of COPD and asthma. Evidence emerged from COPD studies that Tiotropium—in addition to its bronchoprotective effects—reduced the number of exacerbations and positively influenced the course of the disease (49, 50). These beneficial effects may be due to direct or indirect effects of Tiotropium on airway mucus hypersecretion, inflammation and airway remodeling.

a) Receptor binding

In vitro studies with human and animal muscarinic receptor subtypes (M1, M2, and M3) and with human and animal isolated tracheal preparations established Tiotropium as a potent, selective and reversible muscarinic receptor antagonist. No other receptor interactions were detected at relevant concentrations. Association and dissociation from muscarinic receptors (M1, M2, and M3) were slow compared to Ipratropium. The dissociation half-life of Tiotropium-M3-complexes at 23°C was 34.7 hours compared to 0.26 hours for Ipratropium-M3-complexes. Tiotropium-M2-complexes and Ipratropium-M2-complexes dissociate more rapidly than M3- or M1-receptor-complexes. This pattern suggests a “kinetic receptor subtype selectivity” of occupation and blockade of M3>M1>M2-receptors (IB Action and clinical pharmacology p.14-18, reference 48).

b) Bronchoprotective effects

Tiotropium provided dose-related protection against methacholine-induced broncho-constriction in patients with mild to moderate asthma (51).

c) Excretion and drug-drug interaction

Tiotropium is mainly excreted renally. Increased plasma concentrations were described in patients with moderate to severe renal impairment (creatinine clearance ≤ 50 mL/min). A dose reduction based on renal dysfunction cannot be recommended. Tiotropium should only be used in patients with moderate to severe renal impairment if the expected benefit outweighs the potential risk. Drug interactions of Tiotropium with other drugs are unlikely due to the small dose and very low steady-state plasma levels of Tiotropium and the lack of inhibition of cytochrome P450 isoenzymes by Tiotropium (48).

d) Tiotropium inhalation in Patients with Asthma

A total of 14 randomized controlled trials (RCTs) with 4.998 patients in the Tiotropium group and 5.074 patients in the control group were included in a recent review focused on evaluating the efficacy of Tiotropium as add-on therapy to ICS or ICS + LABA in patients with uncontrolled moderate to severe persistent asthma (52). The study concluded that Tiotropium had a beneficial effect in moderate to severe persistent asthma as add on to ICS or ICS and LABA, mainly in increasing morning peak expiratory flow (PEF), evening PEF, peak FEV, and trough FEV. No safety concerns were found with the use of Tiotropium as add-on therapy. Consequently, regulatory authorities worldwide have recently licensed Tiotropium as the only LAMA approved for the add-on treatment for adults, adolescents, and children > 6 years with persistent, symptomatic asthma.

4.1.4 Known side effects

The incidence of AEs was low in all asthma trials so far. The type and rate of events were not different from those seen in the trials with COPD patients, aside from “asthma exacerbation”. The most common AEs were asthma exacerbation, upper respiratory infection, headache, and dry mouth.

<u>Special note on the excipient benzalkonium chloride (BAC):</u>

According to the SmPC, the SPIRIVA Respimat® inhaler—and the placebo inhaler—contains BAC, which may cause wheezing and breathing difficulties. The risk of these adverse events is increased in patients with asthma.
According to the coordinating investigator, this risk is negligible, especially in young children, who are much less likely to be allergic to drugs than adults.

4.1.5 Interaction with other medicinal products and other forms of interaction

Although no formal drug interaction studies have been performed, tiotropium bromide has been used concomitantly with other drugs commonly used in the treatment of COPD and asthma, including sympathomimetic bronchodilators, methylxanthines, oral and inhaled steroids, antihistamines, mucolytics, leukotriene modifiers, cromones, anti-IgE treatment without clinical evidence of drug interactions. Use of LABA or ICS was not found to alter the exposure to tiotropium. The co-administration of tiotropium bromide with other anticholinergic containing drugs has not been studied and therefore is not recommended.

4.1.6 Pharmacodynamic properties

Tiotropium bromide is a long-acting, specific antagonist at muscarinic receptors. It has similar affinity to the subtypes, M1 to M5. In the airways, tiotropium bromide competitively and reversibly binds to the M3 receptors in the bronchial smooth musculature, antagonising the cholinergic (bronchoconstrictive) effects of acetylcholine, resulting in bronchial smooth muscle relaxation. The effect was dose dependent and lasted longer than 24 h. As an N-quaternary anticholinergic, tiotropium bromide is topically (broncho-)selective when administered by inhalation, demonstrating an acceptable therapeutic range before systemic anticholinergic effects may occur.

<u>Pharmacodynamic effects</u>

The dissociation of tiotropium from especially M3-receptors is very slow, exhibiting a significantly longer dissociation half-life than ipratropium. Dissociation from M2-receptors is faster than from M3, which in functional in vitro studies, elicited (kinetically controlled) receptor subtype selectivity of M3 over M2. The high potency, very slow receptor dissociation and topical inhaled selectivity found its clinical correlate in significant and long-acting bronchodilation in patients with COPD and asthma.

4.1.7 Pharmacokinetic properties

Characteristics in patients

Paediatric Patients

Asthma: The peak and total (AUC and urinary excretion) exposure to tiotropium is comparable between patients with asthma who were 6-11 years old, 12-17 years old and ≥ 18 years old. Based on urinary excretion, the total exposure to tiotropium in patients 1-5 years of age was 52-60 % lower than in other older age groups. The total exposure data when adjusted for body surface area were found to be comparable in all age groups. Spiriva Respimat was administered with a valved holding chamber with face mask in patients 1-5 years of age.

4.1.8 Dosage schedule

Different dosage schedules have been tested in children and adolescents. Efficacy, safety, and tolerability of once daily Tiotropium Respimat® 5 µg, 2.5 µg, and 1.25 µg add-on to medium-dose ICS +/- a leukotriene modifier in children aged 6–11 years with symptomatic asthma (53). The primary endpoint (peak forced expiratory volume in 1 second measured within 3 hours post-dosing), the adjusted mean responses with Tiotropium Respimat® 5 µg (272 mL), 2.5 µg (290 mL), and 1.25 µg (261 mL) were significantly greater than with placebo (Figure 3).

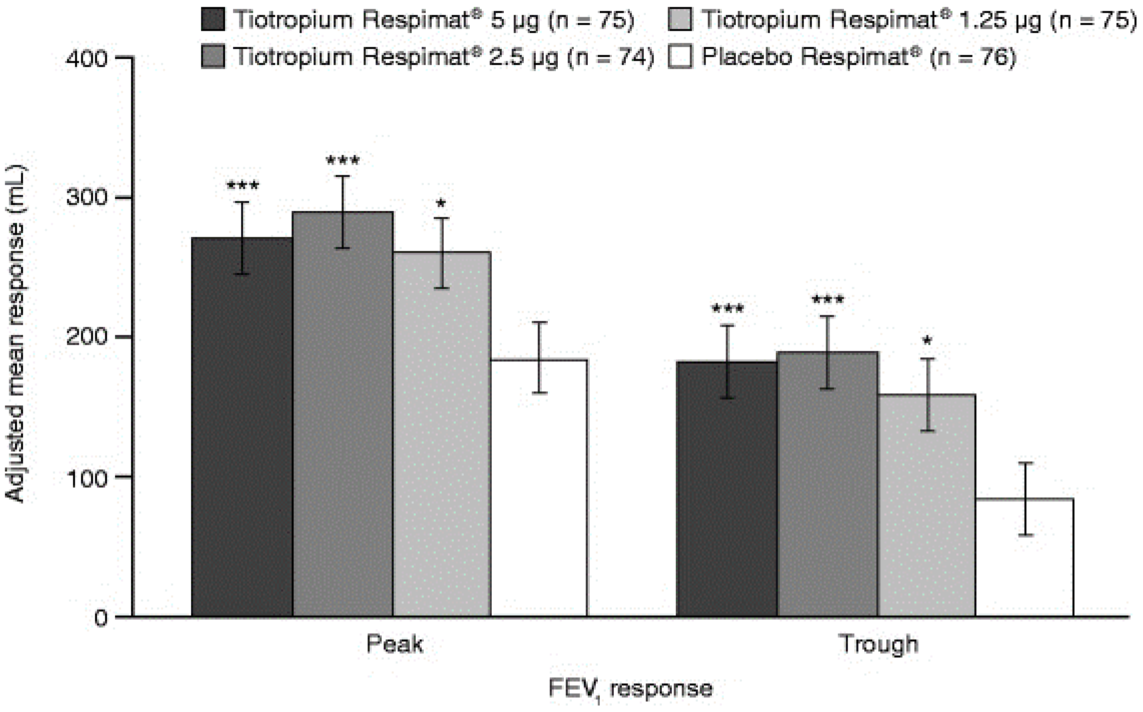

Figure 3: Peak forced expiratory volume in 1 second measured within 3 hours post-dosing.

Interestingly, the FDA licensed 2.5 µg, whereas the EMA licensed 5.0 µg Tiotropium for use in patients with asthma. Based on the results of phase II and III studies 2.5 µg Tiotropium as add-on to ICS therapy was safe and associated with consistent improvements in lung function in patients with asthma of varying severities across different age groups (44). Accordingly, in the planned study we will use the lower dose of 2.5 µg Tiotropium to be effective and to save cost for better patient care.

4.1.9 Overdose instructions

High doses of Tiotropium bromide may lead to signs and symptoms of exaggerated anticholinergic effects, such as constipation, voiding difficulties, or increased intraocular pressure causing pain, vision disturbances, or reddening of the eye. No AEs, beyond dry mouth/throat and dry nasal mucosa in a dose-dependent (10–40 µg daily) incidence, were observed following 14-day dosing of up to 40 µg Tiotropium inhalation solution in healthy patients except for pronounced reduction in salivary flow from day 7 onwards (48).

4.1.10 Treatment assignment

The trial medication will be administered only to patients included in this trial. Patients withdrawn from the trial retain their identification codes (e.g. randomization number). New patients will always receive a new identification code.

4.1.11 Treatment after the end of the trial

After the end of the trial, patients will be treated according to well-accepted national and international recommendations and guidelines.

4.1.12 Packaging and labeling

Labelling and packaging of the trial medication takes place at Almac Ltd., Craigavon. The trial medication will be labeled according to § 5 GCP regulation. The label contains the following details:

- For use in clinical trial only
- Name and address of sponsor
- Name and dose of IMP
- Batch number „Ch.-B. or code number", date of manufacturing
- Administration form
- Content specified by weight, volume, or quantity
- Application form
- Expiry date using note "usable until"

4.1.13 Drug storage, supplies and accountability

The investigator will take inventory and acknowledges the receipt of all shipments of the trial medication.

All trial medication must be kept in a locked area with access restricted to designated trial staff. The trial medication must be stored dry and according to manufacturer's instructions at room

temperature (do not freeze) in its closed primary packaging separated from trading goods. The investigator will also keep accurate records of the quantities of trial medication dispensed, used, and returned by each patient on the drug accountability form.

The site monitor will periodically check the supplies of trial medication held by the investigator to verify the correct accountability of all trial medication used. At the end of the trial, all unused trial medication and all medication containers will either be completely returned to the pharmacy of the University Medical Center Mainz, or destroyed at participating site—in case no other procedure is agreed. Should the medication expire during the study, the unused medication still in stock at the pharmacy of the University Medical Center Mainz can be destroyed by the pharmacy itself. A destruction protocol must be prepared by the pharmacy and provided to the Sponsor and Boehringer Ingelheim. If patients are still undergoing treatment at this time, these patients will receive a corresponding number of new inhalers—according to their treatment allocation—with a new expiration dateDestruction of study medication can be arranged first after permission by the sponsor (refer to IZKS-SOP PPM01).

The investigator will ensure that a final drug accountability report is prepared and archived in the Investigator Site File.

4.1.14 Safety instructions

SPIRIVA® Respimat® is an approved medication in children aged 6 years or older in Germany. The safety instructions are based on the current summary of product characteristics (Fachinformation) for SPIRIVA® Respimat®.

4.1.15 Procedures for monitoring patient compliance

Trial medication will be dispensed to the patient by the investigator. Patients will be instructed to bring all trial medication to the trial site at every visit (including all empty packages and unused trial medication). Compliance will be assessed by counting cartridges. Details will be recorded on the drug accountability form in the Investigator Site File.

4.2 Not permitted medication

Patients' controller medication [ICS and e.g. LTRA, if applicable, including method of delivery (e.g. with a spacer)] should be kept stable throughout the trial, with the exceptions specified in this protocol e.g. in case of asthma exacerbations. Table 1 shows the use or restrictions of the following concomitant treatments during the trial.

Table 1: Required, permitted, and restricted medication.

Drug Class	Sub-class	Screening Period	Treatment Period
Corticosteroids	Inhaled corticosteroids	REQUIRED	REQUIRED
	Systemic (e.g. oral or i.v.) corticosteroids	not permitted[1]	not permitted (except for treatment of asthma exacerbation)
	Topical (e.g. intranasal) corticosteroids (e.g. Nasonex®)	permitted	permitted

Beta-adrenergics	Inhaled short-acting β2-agonists	rescue	rescue
	Inhaled long-acting β2-agonists	permitted[3]	permitted
	Systemic β2-agonists (cave: this includes Spasmo Mucosolvan®)	not permitted[2]	not permitted
Anticholinergics	Inhaled short-acting anticholinergics (inhalation e.g. Atrovent®)	not permitted[2] (except for treatment of asthma exacerbation)	not permitted (except for treatment of asthma exacerbation)
	Inhaled long-acting anticholinergics (e.g. Spiriva®)	not permitted[1]	study medication
Combination medications	Combination ICS/ long-acting β2-agonist (e.g. Viani mite®)	permitted[3]	permitted
	Combination short-acting anticholinergic/ short-acting β2-agonist (e.g. Berodual®)	not permitted[2]	not permitted

**Drug Class**	**Sub-class**	**Study Period**		
		**Screening Period**	**Treatment Period**	**Follow-up Period**
Methylxanthines	Short-acting theophilines (e.g. Theolair®)	not permitted[2] (except for treatment of asthma exacerbation)	not permitted (except for treatment of asthma exacerbation)	not permitted (except for treatment of asthma exacerbation)
	Long-acting theophilines (e.g. Theolair® retard)	not permitted[2,3]	not permitted	not permitted
Miscellaneous	Other investigational drugs	not permitted	not permitted	not permitted
	Leukotriene modifiers (e.g. montelukast Singulair®)	permitted[3]	permitted	permitted
	Treatment with monoclonal antibodies (e.g. omalizumab, Xolair®)	not permitted	not permitted	not permitted
	Antihistamines	permitted	permitted	permitted

	Mucolytics (not containing bronchodilators)	permitted	permitted	permitted

[1] Not permitted within 4 weeks prior to visit 1

[2] Not permitted within 2 weeks prior to visit 1

[3] To be stabilized for at least 4 weeks prior to the trial and stable throughout the trial

Additional restrictions prior to pulmonary function throughout the trial (including visit 1):

- At least an 8-hour wash-out of inhaled short-acting β2-agonists.
- At least a 12-hour wash-out of inhaled LABAs.
- LABAs or ICS + LABA combinations (if applicable) may be administered after completing all lung function measurements
- Restrictions on diet and lifestyle

The following restrictions should be observed prior to/during pulmonary function/Rint testing throughout the trial (including visit 1):

1. Medication wash-out restrictions should be adhered to as described.
2. At visit days (including screening, i.e. visit 1), patients must try to refrain from strenuous activity at least 12 hours prior to pulmonary function and throughout the testing period. Patients are requested to rest at least 10 minutes prior to each measurement. Patients should also avoid cold temperatures, environmental smoke, dust, or areas with strong odors (e.g. perfumes) during the test day.
3. Chocolate, cola, and other caffeine-containing beverages and foods, and ice-cold beverages are not permitted at least 2 hours prior to pulmonary function and until completion of all lung function measurements. Decaffeinated beverages are acceptable.

4.3 Rescue medication or emergency treatment

Salbutamol pMDI via a spacer as needed will be permitted as rescue medication after visit 1 during observation and after randomization throughout the study.

Please refer to section 7.

5 TRIAL SCHEDULE

Please refer to the flow chart (trial schedule and section 3, figure 2) for the examinations and procedures to be performed at visit 1.

Information/instructions visit 1:

Prior to enrolment of a patient, the investigator will complete a preliminary check of major in- and exclusion criteria, in order to keep the number of screen failures as low as possible.

Informed consent from the parent(s)/legal guardian(s) and (if applicable) assent from the child will be obtained prior to the patient's participation in the trial. The date of the last signature obtained from the parent(s)/guardian(s) qualifies as "date of consent" and needs to be entered in the eCRF.

Additional information/instructions visit 1:

Visit 1 may be conducted any time during regular business hours but preferably between 8–13 o'clock in patients ≥ 4 years who will perform spirometry.

- The screening visit should be registered in the eCRF. Further instructions regarding the use of the eCRF are provided in the user manual filed in the ISF.
- The investigator must record all medication used by the patient in the three months prior to visit 1 and throughout the trial on the concomitant therapy eCRF.
- Current conditions and conditions for which therapy is given in the last three months prior to visit 1 as well as any chronic disease (excluding asthma) will be recorded (baseline conditions).
- Demographic data that will be collected includes sex, race, date of birth, height, weight, household/second-hand smoking, household pet, duration of asthma.
- Medical history data that will be collected includes questions about: (premature) gestation, breastfeeding, presence of allergy, history of attention deficit disorder, history of cardiac diseases, history of narrow-angle glaucoma, history of renal/urinary tract diseases, and history of cancer.
- Patients qualified to enter the 4–6 week screening period and their parents/guardians (and other caregivers, if applicable)
  - will be issued rescue medication (or should be instructed to use their own nebulized salbutamol/albuterol when needed)
  - will be issued a TIPP Diary App (PACD), used by the parents/legal guardians .
  - Details of any patient who is screened for the trial but is found to be ineligible must be entered in the screening log and documented in the eCRF.

Investigations at (Baseline) visit 1

- Patient information and informed consent
- Inclusion/Exclusion criteria
- Demographics
- Physical examination

- Vital signs
- Height and weight
- Previous and concomitant diseases
- Previous and concomitant treatments
- TRACK test
- Spirometry/Oscillometry in patients ≥ 4 years
- Laboratory including PAX gene tubes for miRNA
- Dispense/check of rescue medication
- Instructions for using the TIPP diary App (explanation and download) and output of personal login details
- AEs
- Assessment of asthma control according to GINA guidelines
- Concomitant therapy

Investigation at (Randomization) visit 2

All patients must be symptomatic (partly controlled or uncontrolled) as defined by the GINA guideline for children aged 5 years and younger (7) at visit 1 (screening) and before randomization at visit 2.

If this criterion is not met at visit 2, the visit can be repeated once within 2 weeks.

Investigations at visit 2

- V2 will be scheduled 4-6 weeks after V1
- Inclusion/Exclusion criteria
- Physical examination
- Vital signs
- Height and weight
- Previous and concomitant diseases
- Previous and concomitant treatments
- Spirometry/Oscillometry pre and 30 minutes post-dosing in patients ≥ 4 years
- Randomization
- Training of Respimat® inhaler with a Respimat® training device with spacer (Appendix 15.4)
- Dispense of study medication
- Dispense/Check of rescue medication
- Download/review TIPP diary App
- Assessment of asthma control according to GINA guidelines
- AEs
- Concomitant therapy

- Drug accountability

Rescheduling prior to randomization (during screening/run-in period)

- The screening period (between visit 1 and visit 2) may be extended by 7 days (see flow chart), e.g. for administrative reasons.
- In case of severe asthma exacerbation or severe respiratory tract infection (or any other acute illnesses) defined by systemic steroid intake or hospitalization in the 4–6 weeks prior to visit 2, the randomization visit (visit 2) must be postponed until 4 weeks following recovery from the infection or exacerbation.

Investigations at visit 3

- V3 will be scheduled 6 ± 1 weeks after V2
- Physical examination
- Vital signs
- Height and weight
- Previous and concomitant diseases
- Previous and concomitant treatments
- Spirometry/Oscillometry in patients ≥ 4 years
- Download/Review TIPP diary App
- Check study medication compliance
- Collect study medication/drug accountability
- Dispense of study medication
- Dispense/check of rescue medication
- AEs
- Concomitant therapy

Investigations at visit 4

- V4 will be scheduled 10 ± 1 weeks after V3
- Physical examination
- Vital signs
- Height and weight
- Previous and concomitant diseases
- Previous and concomitant treatments
- Spirometry/Oscillometry ≥ 4 years pre-dosing
- Download/review TIPP Diary App
- Check study medication compliance
- Collect study medication/drug accountability
- Dispense of study medication
- Dispense/check of rescue medication

- TRACK test
- Assessment of asthma control according to GINA guidelines
- AEs
- Concomitant therapy

Investigations at visit 5

- V5 will be scheduled 12 ± 1 weeks after V4
- Physical examination
- Vital signs
- Height and weight
- Previous and concomitant diseases
- Previous and concomitant treatments
- Spirometry/Oscillometry ≥ 4 years pre-dosing
- Download/review TIPP Diary App
- Check study medication compliance
- Collect study medication/drug accountability
- Dispense of study medication
- Dispense/check of rescue medication
- Assessment of asthma control according to GINA guidelines
- AEs
- Concomitant therapy

Investigations at visit 6

- V6 will be scheduled 12 ± 1 weeks after V5
- Physical examination
- Vital signs
- Height and weight
- Previous and concomitant diseases
- Previous and concomitant treatments
- Download/review TIPP Diary App
- Check study medication compliance
- Collect study medication/drug accountability
- Dispense of study medication
- Dispense/check of rescue medication
- AEs
- Concomitant therapy

Telephone contacts between visits 4–7

Telephone visits will be performed 6 ± 1 weeks after V4, V5, V6. To monitor the patient's clinical status more closely between site visits 4–7, the patient's parents/guardians will be contacted by phone midway between visits (see also flow chart for scheduled phone contacts). Before the telephone contact, the TIPP Diary APP should be reviewed. During this contact, the trial staff will inquire about the compliance of study medication use, health status, AEs, concomitant diseases and treatments, concomitant therapies, and medication, and exacerbations.

In the case of a suspected worsening of patient's clinical status, the investigator should carefully assess whether further medical assessment or treatment of an asthma exacerbation is required. If any condition qualifies as an AE, all according steps have to be taken.

Investigations at visit 7 (End of trial)

After a 52-week treatment phase, the last visit 7 will be performed. The end of trial visit may be conducted any time during regular business hours and marks the trial completion that must be recorded in the eCRF.

- V7 will be scheduled 12 ± 2 weeks after V6
- Physical examination
- Vital signs
- Height and weight
- Previous and concomitant diseases
- Previous and concomitant treatments
- Spirometry/Oscillometry ≥ 4years pre-dosing
- Laboratory
- Download/review TIPP diary App
- Check study medication compliance
- Collect study medication/drug accountability
- Assessment of asthma control according to GINA guidelines
- TRACK test
- Complete CGI-C at end of treatment
- AEs
- Concomitant therapy

Please refer to the flow chart (trial schedule and section 3, Figure 2) for the examinations and procedures to be performed in case of early termination of patients who have received at least one dose of study medication.

Additional information/instructions visit 7 and in case of early discontinuation:

- Any persistently abnormal test must be fully explained by the investigator and follow-up evaluation needs to be performed, if necessary. Any ongoing (serious) AEs must be followed until the event is resolved or there is a mutual agreement between the investigator and local clinical monitor that follow-up is sufficient.

- The local clinical monitor must be consulted on all persistently abnormal tests and SAEs until it is agreed that follow-up is no longer necessary.
- The end of treatment period will be recorded on the termination of trial medication page of the eCRF.

The last-patient-out date of the overall trial will be determined by the time point when the last patient finished the complete trial including the 52-week treatment period. There is no extension trial intended. After the trial, the patients will return to standard medical care according to national and international treatment guidelines, as provided by their treating (pediatric) pulmonologist or general practitioner.

6 TRIAL METHODS

6.1 Assessment of efficacy

The primary efficacy endpoint is the time to first severe exacerbations defined by hospitalization and/or at least 3 days of systemic steroids or one day of rectal prednisolone (Rectodelt®). Severe exacerbations were chosen as primary outcome because they are patient-important outcomes that have significant consequences for children, caregivers, and the health care system.

Key secondary endpoint(s) for all patients: Secondary endpoints for all patients:

- Number of severe exacerbations
- Exacerbation-free survival time defined as the time from randomization until the first severe exacerbation.
- Percentage of patients requiring systemic corticosteroid rescue for asthma during the 52-week treatment period.
- Number of hospitalizations (severe exacerbation).
- Percentage of days and episodes of $\geq$3 days with use of PRN salbutamol rescue medication during 52-week treatment period (non-severe exacerbation).
- Percentage of days without asthma symptoms assessed by TIPP diary App (PACD).
  - A day without asthma symptoms is defined as a day during which the patient experienced no asthma symptoms (daytime and overnight; question 1, 2, 5, 6 , did not use rescue medication (salbutamol) (question 3 and 7), and had no asthma exacerbation/worsening requiring systemic corticosteroids, an unscheduled visit to a doctor's office, or hospital (question 11).
- Percentage of night-time awakenings due to asthma symptoms as assessed by the TIPP diary App.
- Health utilization defined as number of physician visits during treatment.
- Number of missed days in daycare.
- Number of asthma-related events defined by use of antibiotics.
- Evaluation of TRACK at V1, at V4 and at end of treatment.
- CGI-C at end of treatment.

Additional secondary endpoints for children aged 4 years and older:

Only applicable for patients able to perform technically acceptable (and reproducible) pulmonary function tests.

- The forced expiratory volume in one second (FEV1) and the forced vital capacity (FVC) measured 30 minutes post IMP will be compared to pre-dosing FEV1 and FVC values at visit 2.
- Trough FEV1 and through FVC response will be determined at visit 3, visit 4, visit 5 and visit 7. Trough FEV1 is defined as the FEV1 and FVC measured approximately 24 hours after the last IMP inhalation. Response is defined as a 10% improvement in FEV1 or FVC, respectively, compared to baseline (visit 2, pre-dosing).
- Potential biomarkers for treatment response (Eosinophils, IgE, specific IgE to common allergens: birch, grass, *Dermatophagoides pteronyssinus*, *Alternaria*, *Cladosporium*, cat, dog, hen's egg, cow's milk and peanut)

6.2 Physical Examination

This evaluation will include an examination of general appearance, skin, eyes, ears, nose, throat, lungs, and heart. Information about the physical examination must be present in the source documentation at the study site. Significant findings that are present before the start of study drug must be included in the relevant medical history/current medical conditions of the eCRF page. The order of the completion of the questionnaires and of conducting the other assessments performed at each visit is important.

PACD

The PACD will be used to evaluate daily asthma symptoms in children aged 1–5 years (54). The electronic diary includes various questions that have to be answered in the evening for daytime symptoms and the previous night. The combined daytime score is the average of scores from questions 4–7 in the diary which are questions regarding severity of cough, wheezing, trouble breathing, and interference with activities, scores for each question range from 0 (best) to 5 (worst). Using a symptom diary during 12 months will allow the symptom load of the patients and the asthma severity in treated vs. control patients to be defined and documented. It is preferred the same parent/guardian answers the questions during the study. The PACD questions will be collected electronically by the TIPPDiary app.
In order to reduce the burden of parents, the electronic diary should not be filled out daily all year round. It is well known from many studies that asthma exarcerbations occur particularly in autumn, winter and spring. For this reason, no diary should be kept for 4 months in the months of May, June, July and August. If the patients are recruited in the summer months, they only need to keep an electronic diary between visit 1 and visit 2.

TRACK

The TRACK test (56) is used to evaluate the respiratory and asthma control at V1, at V4, and at the end of study at V7 (Figure 4).

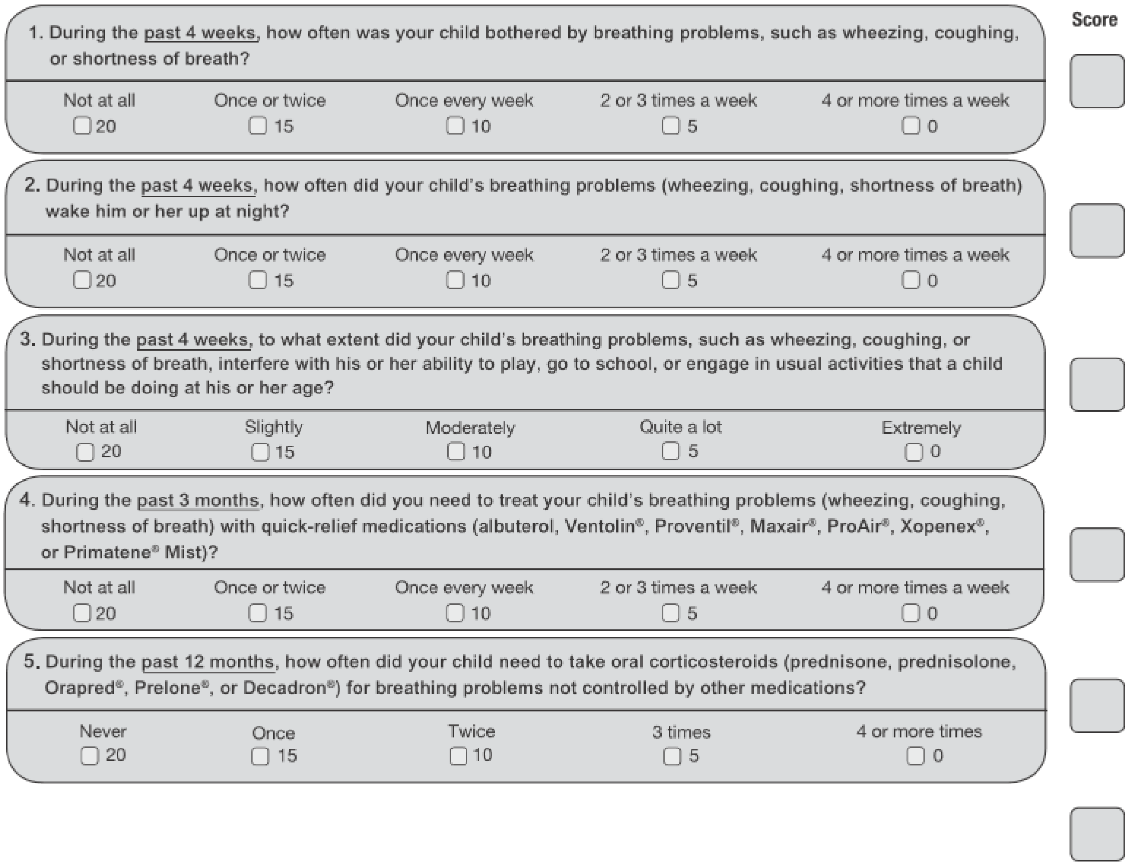

Score

1. During the past 4 weeks, how often was your child bothered by breathing problems, such as wheezing, coughing, or shortness of breath?

Not at all	Once or twice	Once every week	2 or 3 times a week	4 or more times a week
☐ 20	☐ 15	☐ 10	☐ 5	☐ 0

2. During the past 4 weeks, how often did your child's breathing problems (wheezing, coughing, shortness of breath) wake him or her up at night?

Not at all	Once or twice	Once every week	2 or 3 times a week	4 or more times a week
☐ 20	☐ 15	☐ 10	☐ 5	☐ 0

3. During the past 4 weeks, to what extent did your child's breathing problems, such as wheezing, coughing, or shortness of breath, interfere with his or her ability to play, go to school, or engage in usual activities that a child should be doing at his or her age?

Not at all	Slightly	Moderately	Quite a lot	Extremely
☐ 20	☐ 15	☐ 10	☐ 5	☐ 0

4. During the past 3 months, how often did you need to treat your child's breathing problems (wheezing, coughing, shortness of breath) with quick-relief medications (albuterol, Ventolin®, Proventil®, Maxair®, ProAir®, Xopenex®, or Primatene® Mist)?

Not at all	Once or twice	Once every week	2 or 3 times a week	4 or more times a week
☐ 20	☐ 15	☐ 10	☐ 5	☐ 0

5. During the past 12 months, how often did your child need to take oral corticosteroids (prednisone, prednisolone, Orapred®, Prelone®, or Decadron®) for breathing problems not controlled by other medications?

Never	Once	Twice	3 times	4 or more times
☐ 20	☐ 15	☐ 10	☐ 5	☐ 0

Figure 4: Test for respiratory and asthma control in kids (TRACK).

CGI-C

Caregivers evaluate the overall treatment effect at the end of the 52-week treatment period using a 7-point scale to answer the following question:
"Check the one number that best describes how your child's asthma is now, compared with how it was before your child began taking medication in this study." Possible answers: very much better, much better, a little better, no change, a little worse, much worse, very much worse.

Pulmonary Function Testing

a) Spirometry

PFTs will only be performed in ≥ 4-year-olds who are capable of providing PFTs of acceptable quality. Repeatability criteria for preschoolers should be used: The patient should produce at least two acceptable curves, where the second-highest FVC and FEV1 are within 0.1 L or 10% of the highest value, whichever is greater (56). In a recent own investigation we found the following: According to the new American Thoracic Society/European Respiratory Society (ATS/ERS) guidelines, 74.6% of the children aged 4–6 years could perform at least 2, repeatable measurements in the healthy status (57, 58).

PFTs will be recorded whilst the patient is in a sitting position at the time points specified in the study flow chart. Spirometry will be performed according to the ERS. All lung function tests will

be repeated until three technically acceptable measurements have been made and the best of these three measures will be recorded in the CRF. The following variables will be documented: VC [L, % pred], FEV1 [L, % pred], FVC [L, % pred], FEV1/FVC (Tiffenau-Index).

Calibration of spirometry equipment is mandatory on all visit days and calibration logs should be stored as source data. The spirometers and their use, including daily calibration, must meet ATS/ERS criteria (57). If necessary, the sponsor may provide a SOP to the centers.

b) Impulse oscillometry

Impulse oscillometry (IOS) is a breathing analysis at rest that requires little cooperation. Thus, it can be performed in relatively young children. IOS parameters can differentiate between central and peripheral airways. There is evidence that asthma exacerbations in infancy are related to an inflammation of the small airways. In an own study, we included 75 children aged 4–7 years with mild asthma in the infection-free interval in the summer (58). The results showed that the IOS predicted significantly mild asthma exacerbations in autumn and winter (AUC: Rrs5 78%, Xrs5 70%, and Rrs5-20 77%). This was not the case for spirometry (FEV1) and methacholine provocation (both AUC 61%).

c) IOS measurement

The child should sit in an upright position and calmly exhale and inhale. To avoid vibrations of the upper respiratory tract the cheeks are held with hands. Mouthpiece and a nose clip are mandatory. For scientific purposes, at least 3 acceptable measuring cycles of 8 to 16 seconds each are required; the resistance (Rrs) serves as the reference value. Possible incorrect measurements result from swallowing, inadequate mouth closure, or glottis closure. The IOS measurement is carried out before spirometry (59, 60). The most stable and best-tested parameters regarding intra and between measurement repeatability are the resistance (Rrs) and the reactance (Xrs) (60). The Rrs at 5 Hz (Rrs5) represents the peripheral and central airways and the Rrs 20 the central airway. The resistance of the peripheral airways can be calculated using the difference between Rrs5 and Rrs20, the Rrs5-20. The Xrs5 represents the retraction capacity of the lungs and the volume of the lung periphery. Another parameter for the lung periphery is the reactance area (AX), the integral of the reactance from Xrs5 to Fres (60). An obstruction of the peripheral airways is reflected by increased Rrs5-20 and, due to the loss of the elastic restoring forces, by a low Xrs5 as well as an increase of AX (61).

There are several studies for the collection of reference parameters for the Rrs and Xrs. For Rrs5, reference values < 140% are considered normal (Table 2), from 140% to 150% as gray areas. At values > 150% there is a pathologically increased airway resistance. Values > -0.15 kPa*s*L-1 are normal for the Xrs5 (59, 60). No reference parameters have yet been published for Rrs5-20 and AX; the value of these measurements applies to the difference before and after bronchodilation or therapeutic interventions.

Table 2: IOS reference values and cut points.

	**Parameter**			
	**Rrs5**	**Xrs5**	**Rrs5-20**	**AX**
Reference values	< 140%	> -0.15 kPa* s*L^{-1}	-	-
Cut points broncho-dilatation	≥ 40%	-	-0.23 kPa*s*L^{-1} , z-score -0.69	-2.14 kPa*L^{-1}, z-score -1.40

6.3 Other assessments

6.3.1 Previous and intercurrent illnesses

Illnesses already known at the time of informed consent are to be documented in the eCRF as medical history. Illnesses detected during the clinical trial are to be documented as AEs (see section 7).

6.3.2 Previous and intercurrent medical treatments

All medical treatments except for the investigational medical products received by the participant at the beginning, or during the clinical trial, or both, are to be documented in the eCRF as concomitant medication.

<u>Special situation:</u>

In the event of a necessary intensive medical treatment (i.e. child in intensive care unit), all medications administered specifically for the treatment of asthma must be documented. For all other medications such as antibiotics, sedatives/anesthetics, nutritional therapeutics, topical medications (e.g. eye, ear, inhalation) and homeopathy, no information on date, dosage and route need to be provided.

6.3.3 Laboratory parameters and vital signs

All Laboratory parameters and vital signs are to be documented in the eCRF. The following parameters will be analyzed in this trial:

- Differential blood count
- Glutamate-Pyruvate-Transaminase (GPT)
- Glutamate-Oxalate-Transaminase (GOT)
- Alkaline Phosphatase (AP)
- Urea
- Creatinine
- Total IgE
- Rast (10 Allergens)

Topical (percutaneous) analgesia to reduce pain associated with venipuncture, e.g. amethocaine gel or EMLA cream (Eutectic Mixture of Local Anesthetics), must be applied (may only be omitted in patients who explicitly refuse it). A maximum of 3 puncture attempts to obtain blood (approx. 5 mL) will be performed.
Additionally, we perform next generation sequencing (NGS), enabling analysis of microRNA (miRNA) expression at V1 only (2.5 mL blood).

6.4 Epigenetic regulation

In the last decade, the epigenetic regulation of genes has increasingly become the focus of science. Besides histone modification and DNA methylation, non-coding micro ribonucleic acids (miRNAs) are part of the epigenome and regulate a large number of genes post-transcriptionally through interaction with messenger ribonucleic acids (mRNAs). In this way, miRNAs play a

central role in the control of several cellular processes such as cell proliferation, cell differentiation and apoptosis. Dysregulated miRNA expression has been identified in several diseases and isalso associated with the pathogenesis of asthma and a number of other lung diseases.

Numerous studies have investigated post-transcriptional regulation in allergy and asthma, but almost no data are available for the particularly vulnerable age of 1-5 years for asthmatics.

6.4.1 miRNAs in Asthma

miRNA-146a plays a central role in the pathogenesis of asthmatic inflammation. Its expression is induced by TNF-α and suppressed by glucocorticoids. miRNA expression profiles of adult asthmatics showed that an increase in miR-146a in plasma was associated with the presence of other reverse markers such as increased blood eosinophil counts, higher asthma control test (ACT) scores and the need for higher doses of ICS. However, inoculation of miR-146a into A549 cells treated with TNF-α +/- glucocorticoids produced an anti-inflammatory effect and increased the efficacy of glucocorticoids by suppressing the TNF- α and IL-6 pathway (65). In addition, their potential role as inflammatory markers in atopic dermatitis has been highlighted (41).

In addition to miRNA-146a, miRNA-21 is increased in expression in childhood asthma and plays a role in eosinophilic asthmatic inflammation (67). Its expression is stimulated by IL-13 (67). MiR-21 induces polarization of naïve T cells towards the Th2 lineage and the synthesis of pro-inflammatory cytokines. Wu et al. reported that miR-21 expression was significantly upregulated in bronchial epidermal cells from asthma patients regardless of treatment (68). There is also a linear relationship between plasma miR-21 and miR-146a expression and FEV1 in pediatric cohorts (69).

Thus, miRNA-146a and -21 seem to be ideal surrogate markers for atopic march. Taken together, NGS is expected to demonstrate dysregulation of miRNA-146a and -21 in preschool asthma compared to healthy controls.

In 2017, British authors published miRNA studies from the CAMP cohort, in which they described eight significantly dysregulated miRNA in childhood asthma that correlated with the threshold of irritation in the MCT. These were: hsa-miR-138-5p, hsa-miR-16-5p, hsa-miR-30d-5p, hsa-miR-203a-3p, hsa-miR-128-3p, hsa-miR-638 (70). The strongest significance was found for miR-296, whose target is IKBKE, among others (70). Targeting IKBKE attenuates airway responsiveness and inflammation in a murine asthma model. Furthermore, IKBKE is a well-described target of the NFκB- gene (71). The impact of the NFκB pathway on asthma and inflammation includes modulation of BHR in mice with allergen challenge (72). Furthermore, IKBKE itself is a known therapeutic target for asthma, whereby targeting IKBKE has been shown to significantly attenuate airway responsiveness and inflammation in a mouse model of asthma (73). Therefore, miR-296 could attenuate the immune response and modulate AHR via the NFκB pathway. Moreover, miR-16-5p was significantly associated with BHR, whose aberrant expression in asthmatic airway cells and its modulatory effect on numerous genes have been described (74).

Based on current research, miRNA profiles represent a highly interesting target to investigate treatment response in preschool asthma. MiRNA profiles provide the potential to serve as easily obtainable biomarkers.

The following objectives are being investigated:

1. To investigate the miRNA profile in children with preschool asthma compared to healthy children
2. To identify a specific miRNA profile characterizing subgroups of preschool asthma

7 SAFETY

Safety of the study will be ensured by monitoring of symptom score and rescue medication and lung function. Salbutamol pressurized metered dose inhalers (pMDI) via a spacer as needed will be permitted as rescue medication after visit 1 during observation and after randomization throughout the study.

An asthma exacerbation is defined as an increase of rescue medication (RABA) of more than 6 puffs per day compared to baseline and the use of systemic glucocorticosteroids.

Parents are advised to give in case of a severe asthma exacerbation systemic glucocorticosteroids (Celestamine for 3 days according to manufacture; see table 4 Appendix 15.2) by oral route and contact the investigator within 24 hours.

7.1 Definitions

7.1.1 Adverse event (AE)

According to GCP, an AE is defined as any untoward medical occurrence in a patient or clinical investigation patient administered a pharmaceutical product and which does not necessarily have a causal relationship with this treatment. An AE can therefore be any unfavorable and unintended sign (including an abnormal laboratory finding), symptom, or disease temporally associated with the use of a medicinal (investigational) product, whether or not related to the medicinal (investigational) product.

An AE may be:

- a new symptom or a new disease/diagnosis
- an accident
- a change in laboratory parameters
- worsening of a previous medical condition/disease
- recurrence of a disease
- an increase in frequency or intensity of episodic diseases.

<u>Surgical procedures</u> themselves are not AEs; they are therapeutic measures for conditions that require surgery. The condition for which the surgery is required may be an AE. Planned (elective) surgical measures permitted by the clinical trial protocol and the condition(s) leading to these measures are not AEs if the condition leading to the measure was present before inclusion in the trial. In the latter case, the condition should be reported as medical history.

<u>Change in laboratory parameters:</u> The criteria for determining whether an abnormal test finding should be reported as an adverse event are as follows.

The test result:

- is associated with accompanying symptoms, and/or
- requires additional diagnostic testing, and/or
- leads to an unscheduled change in trial dosing, or discontinuation from the trial, and/or a medical/surgical intervention
- is considered to be medically relevant by the investigator or sponsor.

7.1.2 Serious adverse event (SAE)

A SAE is any untoward medical occurrence that at any dose

- results in death,
- is life-threatening,
- requires patient hospitalization or prolongation of existing hospitalization,
- results in persistent or significant disability/incapacity or
- is a congenital anomaly/birth defect or
- is an important medical event.

Death is an outcome of an event. The event that resulted in death should be recorded and reported as SAE.

Life-threatening means that the patient was at immediate risk of death at the time of the SAE; it does not refer to a SAE that hypothetically might have caused death if it were more severe.

If the admission is pre-planned (i.e., elective or scheduled surgery arranged before the start of the trial) or not associated with an AE (*e.g.,* social hospitalization for purpose) or results in a hospital stay less than 12 hours, the serious criterion “hospitalization” is not fulfilled. However, it should be noted that invasive treatment during a hospitalization (less than 12 hours) may fulfill the criteria of “medically important” and may be reportable as a SAE dependent on clinical judgment.

An inpatient rehabilitation program per se does not fulfill the seriousness criteria “hospitalization”. In this case, the investigator should evaluate carefully whether the reason for the inpatient rehabilitation program has to be documented as an AE/SAE.

Persistent or significant disability or incapacity means that there is a substantial disruption of a person’s ability to carry out normal life functions. The irreversible injury of an organ function (*e.g.,* paresis, diabetes, cardiac arrhythmia) fulfills this criterion.

Important medical events that may not be immediately life-threatening or result in death or hospitalization but may jeopardize the patient or may require intervention to prevent one of the other outcomes listed in the definition above, should also usually be considered serious. Examples of such events include allergic bronchospasm requiring intensive treatment in an emergency room or at home, blood dyscrasias, convulsions that do not result in patient hospitalization, or the development of drug dependency or drug abuse. A diagnosis of cancer during treatment should also be considered as medically important.

7.1.3 Clarification of the difference in meaning between "serious" and "severe":

The terms “serious” and “severe” are not synonymous but are often used interchangeably. The term ‘severe’ is used to describe the intensity (severity) of a specific event; the event itself, however, may be of relatively minor significance (such as severe headache). This is not the same as “serious”, which is based on the existence of one of the above-mentioned seriousness criteria.

7.1.4 Suspected unexpected serious adverse reaction (SUSAR)

A SUSAR is every SAE with an at least possible relationship to the IMP which is unexpected.

An unexpected serious adverse reaction is any adverse reaction, the nature or severity of which is not consistent with the applicable reference safety information.

7.1.5 Onset and end date of AEs and SAEs

The onset date of the AE is defined as the date when new signs or symptoms or worsening of a pre-existing condition first occur. The onset date of the SAE is defined as the date when at least one of the above-listed criteria for seriousness occurs.

The end date of the AE is defined as the date when the symptoms resolve, or the event is considered stable by the investigator. The end date of the SAE is defined as the time the seriousness criteria are no longer applicable. The end date of the SAE must not be later than the end date of the corresponding AE. AEs and SAEs that are ongoing at the time of death are considered not resolved or resolving.

7.2 Assessment of AEs by investigator

Patients must be carefully monitored for AEs by the investigator. The intensity of the AEs and the causal relation to trial medication and/or procedures are to be assessed.

7.2.1 Intensity/Severity

The maximum intensity of an AE will be assessed by the investigator as follows:

Mild: Temporary event which is tolerated well by the patient and does not interfere with normal daily activities.

Moderate: Event which results in discomfort for the patient and impairs their normal activity.

Severe: Event which results in substantial impairment of normal activities of patient.

If the event is serious, the severity reported in the AE must be consistent with the severity included in the SAE report.

7.2.2 Causal relation to trial medication/procedures

The assessment of the relationship of an AE to the administration of study drug is a clinical decision by the investigator based on all available information at the time of the documentation. Factors to be considered in assessing the relationship of the AE to study drug include:

- The temporal sequence from drug administration: The investigator has to consider if the event occurred before the first intake of study medication or long time after the last intake. Additionally, the length of time from drug exposure to event should be evaluated in the

clinical context of the event.

- Recovery on discontinuation (de-challenge), recurrence on reintroduction (re-challenge): Patient's response after drug discontinuation or after drug re-introduction should be considered in the view of the usual clinical course of the event in question.
- Underlying, concomitant, or intercurrent diseases: Each report should be evaluated in the context of the natural history and course of the disease being treated and any other disease the patient may have.
- Concomitant medication or treatment should be examined to determine whether any of them may be suspected to cause the event in question.

Positive causal relationship:

An assessment of 'related' implies a reasonable possibility of a causal relationship between the event and the IMP.

- This means that there are facts (evidence) or arguments to suggest a causal relationship, such as:
- A close temporal relationship
- A common drug reaction to the IMP
- No plausible alternative cause

Negative causal relationship:

An assessment of 'not related' means that there is no reasonable possibility of a causal relationship between the event and the IMP.

This includes for example:

- Another cause of the AE is more plausible
- A temporal sequence cannot be established with the onset of the AE and administration of the study treatment
- A causal relationship is considered biologically implausible.

7.3 Period of observation

In this trial, the period of observation for collection of AEs extends from the time the subject has signed the informed consent document up to the end of trial visit (regular visit 7).

If the investigator detects a serious adverse event in a trial patient after the end of the period of observation, and considers the event related to the trial treatment or study medication, he should document and report the SAE as described in 7.5.

7.4 Documentation of AEs and follow-up

All AEs (whether serious or not) reported by the patient or detected by the investigator will be documented on the appropriate pages of the electronic case report form (eCRF). AEs must also be documented in the patient's medical records.

If the AE is serious (see section 7.1), the investigator must complete, in addition to the "Adverse Event Page", a "Serious Adverse Event Form" at the time the SAE is detected.

Every attempt should be made to describe the AE in terms of a diagnosis. If a clear diagnosis has been made, individual signs and symptoms will not be recorded unless they represent atypical or extreme manifestations of the diagnosis, in which case they should be reported as separate events. If a clear diagnosis cannot be established, each sign and symptom must be recorded individually.

All patients who have AEs, whether considered associated with the use of the investigational products or not, must be monitored until the outcome of the event can be determined, even after the end of the period of observation, but no longer than the individual end of the trial for this patient. The clinical course of the AE will be followed up according to accepted standards of medical practice.

Should the AE result in death, a full pathologist's report should be supplied, if possible.

7.5 Immediate reporting of SAEs by investigator

SAEs must be immediately (at latest within 24 hours of the investigator's awareness) reported to:

IZKS Mainz

Safety Management

University Medical Center Mainz

FAX 06131 / 17–9916

E-mail: fax-ams@izks-mainz.de

The initial SAE report should be as complete as possible including the essential details of patient's identification (patient number), information on the reporter, the serious adverse event (medical term, diagnosis), the trial medication, and the assessment of the causal relationship between the event and the trial medication. The SAE report must be reviewed and signed by the investigator.

The investigator should provide additional information on the clinical course and the outcome of each SAE as soon as possible (follow up report).

The "Serious Adverse Event Form" is provided in the Investigator Site File.

The investigator can contact the monitor, the safety coordinator, or the trial coordinator in case of questions according to documentation or reporting of SAEs.

7.6 Safety evaluation and reporting by sponsor

The sponsor will ensure that all legal reporting requirements are met. According to GCP, the sponsor is responsible for the continuous safety evaluation of the investigational product(s) and the clinical trial.

SUSARs and safety issues as defined by GCP regulation are determined for expedited reporting: The competent authorities and the ethics committees should be notified as soon as possible but not later than 15 calendar days, and 7 calendar days if it was fatal or life-threatening. All

investigators and the members of the data monitoring committee (DMC) will be informed within the same timeframe. The marketing authorization holder of the IMP should be informed too.

Any safety issues requiring a re-evaluation of the benefit-risk relationship of the IMP will also be reported to the competent authorities and the ethics committees as soon as possible but not later than 15 calendar days (GCP regulation § 13, section 4).

Workflow and procedures concerning safety management will be described in a separate document.

During the clinical trial, IZKS Mainz will submit the Development safety update report (DSUR) including a list of all serious adverse reactions to the ethics committee, the competent authority, and the members of the DMC once a year.

7.7 Documentation of special situations

Special situations such as study medication abuse, misuse, overdose, and medication errors including significant dilution and infusion rate errors have to be documented in the subject’s CRF and source documents.

Any special situation with the investigational product occurring during the clinical trial must be reported immediately (at latest within 24 hours of the investigator’s awareness) using the “Special Situation Report Form” to:

IZKS Mainz

Safety Management

University Medical Center Mainz

FAX 06131 / 17–9916

E-mail: fax-ams@izks-mainz.de

If the special situation leads to an adverse event, then only this adverse event has to be documented and reported as an AE/SAE.

7.8 Emergency procedures

During and following a patient’s participation in the trial, the investigator should ensure that adequate medical care is provided to a patient for any AEs including clinically significant laboratory values.

8 STATISTICS

Details of the statistical analysis of the data collected in this trial will be documented in a Statistical analysis plan (SAP) that will be generated by IZKS Mainz and finalized before closing the database and prior to breaking the blind. The SAP is based on the protocol including all amendments. The document may modify the plans outlined in this protocol; however, any major modifications of the primary endpoint definition and/or its analysis will also be reflected in a protocol amendment. Any deviation from the original statistical plan must be described and justified in the final report. The statistical analysis will be conducted using SAS®.

8.1 Sample size

Sample size calculations were based on results published by Vrijlandt E et al. (33). In this study, children aged 1–5 were treated with Tiotropium 5 µg vs placebo as add-on to ICS. After 105 days, 29% of the Tiotropium 5µg group had an asthma exacerbation with pneumonia or asthma worsening, whereas 55.9% of the placebo group had an event. For the present study, it can be assumed that most patients treated with ICS + placebo will suffer from one severe exacerbation within one year, therefore a rate of 90% of placebo patients with a severe exacerbation after one year is anticipated. For the Tiotropium group, a rate of 75% is assumed. The estimation of the difference in rates between the placebo and the Tiotropium group is performed conservatively, assuming a smaller difference than observed after 105 days in the study of Vrijlandt E et al (33). The assumed dropout rate of 15% is converted into a group-loss hazard (0.16) and considered in the sample size calculation. With an event rate of 90% in the control group and 75% in the Tiotropium group and a drop-out-rate of 15%, the sample size will amount to 152 patients when applying a two-sided log-rank test with a hazard ratio of 0.60, a significance level of alpha = 0.05 and a power of 0.8. The power calculation was performed with PROC POWER in SAS® 9.4.

8.2 Analysis populations

All patients whose parents/guardians signed informed consent are considered as enrolled.

All randomized patients will be included in the intention-to-treat (ITT) population. This population is the primary analysis population. Within ITT population analyses patients will be assigned to the treatment to which they were randomized.

To be eligible for the per-protocol population, patients must fulfill the following criteria:

- No concomitant treatment with oral antibiotics longer than 14 days or any monoclonal antibody
- Treatment compliance of at least 80%.

The safety population comprises all patients who received at least one dose of trial treatment. In analyses of the safety population patients will be assigned to the treatment which they actually received. The analysis populations will be defined before unblinding of the study.

8.3 Efficacy analyses

The primary population for the analyses of efficacy is the ITT population.

Determination of primary and secondary measures

The primary efficacy endpoint is the time to first severe exacerbations defined by hospitalization and/or at least 3 days of systemic steroids or one day of rectal prednisolone (Rectodelt®). Severe exacerbations were chosen as primary outcome because they are patient-important outcomes that have significant consequences for children, caregivers, and the health care system.

Key secondary endpoint(s) for all patients: Secondary endpoints for all patients:

- Number of severe exacerbations
- Exacerbation-free survival time defined as the time from randomization until the first severe exacerbation.
- Percentage of patients requiring systemic corticosteroid rescue for asthma during the 52-week treatment period.
- Number of hospitalizations (severe exacerbation).
- Percentage of days and episodes of > 3 days with use of PRN salbutamol rescue medication during 52-week treatment period (non-severe exacerbation).
- Percentage of days without asthma symptoms assessed by TIPP diary App (PACD).
  - A day without asthma symptoms is defined as a day during which the patient experienced no asthma symptoms (daytime and overnight; question 1, 2, 5, 6 , did not use rescue medication (salbutamol) (question 3 and 7), and had no asthma exacerbation/worsening requiring systemic corticosteroids, an unscheduled visit to a doctor's office, or hospital (question 11).
- Percentage of night-time awakenings due to asthma symptoms as assessed by the TIPP diary App.
- Health utilization defined as number of physician visits during treatment.
- Number of missed days in daycare.
- Number of asthma-related events defined by use of antibiotics.
- Evaluation of TRACK at V1, at V4 and at end of treatment.
- CGI-C at end of treatment.

Additional secondary endpoints for children aged 4 years and older:

Only applicable for patients able to perform technically acceptable (and reproducible) pulmonary function tests.

- The forced expiratory volume in one second (FEV1) and the forced vital capacity (FVC) measured 30 minutes post IMP will be compared to pre-dosing FEV1 and FVC values at visit 2.
- Trough FEV1 and through FVC response will be determined at visit 3, visit 4, visit 5 and visit 7. Trough FEV1 is defined as the FEV1 and FVC measured approximately 24 hours after the last IMP inhalation. Response is defined as a 10% improvement in FEV1 or FVC, respectively, compared to baseline (visit 2, pre-dosing).
- Potential biomarkers for treatment response (Eosinophils, IgE, specific IgE to common allergens: birch, grass, *Dermatophagoides pteronyssinus*, *Alternaria*, *Cladosporium*, cat,

dog, hen's egg, cow's milk and peanut)

Assessment of safety: Safety will be ensured by analysis of symptom scores and rescue medication by electronic diary, and AEs.

8.3.1 Definition and analysis of primary endpoint

The primary analysis is the comparison of time to first severe exacerbation within 12 months of therapy between groups. This means especially that observations after 12 months will be censored. Dropouts because of lack of efficacy will be considered as an exacerbation. All other dropouts will be censored. The comparison will be done on a two-sided significance level of α = 5%.

The primary hypotheses are:

H_0: $h_E/h_C = 1$ versus **H_1: $h_E/h_C \neq 1$**

Where h_E, h_C are the hazards in the experimental treatment group and the control treatment group respectively.

The time to first severe exacerbation will be analyzed by Cox regression with factor treatment group and the covariates age group and center assuming a constant hazard ratio over time. The constant hazard ratio assumption will be checked by Schoenfeld residuals. The primary analyses will be performed in the ITT population. Treatment differences will be displayed by estimates of the hazard ratios and 95% confidence intervals.

Several sensitivity analyses will be performed. The primary analysis Tiotropium + at least ICS versus placebo + ICS will be repeated additionally adjusted for LABA and LTRA use. This analysis is not primary since LABA use is not treatment independent. Additional covariates might be considered in the Cox model as well as the assessment of the per-protocol effect (both specified in the SAP). A time to recurrent event analysis is also performed. Since a substantial correlation between the events is expected a Prentice Williams and Peterson (PWP) model with the age group and center will be employed. Additionally, a Kaplan-Meier analysis including a Kaplan-Meier plot will be done.

8.3.2 Analysis of secondary endpoints

The number of severe exacerbations, the number of hospitalizations as well as the number of asthma-related events defined by use of antibiotics will be assessed by negative binomial regression with time within study as offset variable. Exacerbation-free survival time will be analyzed by Cox regression. Further secondary endpoints will be assessed by chi-square or Fisher's exact test for categorical variables or t-test or Mann-Whitney-U-test for continuous variables.

All tests are two-sided, and a p-value of less than 0.05 is considered to indicate statistical significance. Treatment differences will be displayed by hazard ratios, odds ratios, and 95% confidence intervals. All the analyses will be performed with the use of SAS® software, version 9.4 (SAS Institute).

8.3.3 Analysis of subgroups

Subgroups might be defined in the SAP.

8.3.4 Interim analyses

No interim analyses are planned.

8.4 Analysis of AEs

All summaries and listings of safety data will be performed for the safety population.

AEs will be coded according to MedDRA terminology. Detailed information collected for each AE will include a description of the event, duration, whether the AE was serious, intensity, relationship to trial drug, action taken and clinical outcome. Summary tables will present the number of patients observed with AEs by MedDRA System Organ Class and Preferred Term and corresponding percentages. Additional subcategories will be based on event intensity and relationship to trial drug.

A patient listing of all AEs will be prepared.

8.5 Analysis of clinical laboratory findings

Structured listings will be prepared for each laboratory measure to permit review of the data per patient as they progress on treatment.

Summary tables will be prepared to examine the changes of laboratory measures over time.

9 QUALITY CONTROL, QUALITY ASSURANCE AND RISK MANAGEMENT

The sponsor will continuously manage quality and risks to ensure patient protection and reliability of trial results. The measures used will be proportionate to the inherent risks and the importance of the data collected.

9.1 Requirements for Investigator, investigational sites and members of the investigating staff

The investigator should be able to demonstrate (e.g. based on retrospective data) a potential for recruiting the required number of suitable patients within the agreed recruitment period.

The investigator should have sufficient time to properly conduct and complete the trial within the agreed trial period. The investigator should have available enough qualified staff and adequate facilities for the foreseen duration of the trial to conduct the trial properly and safely.

The investigator has to select his investigating team and ensure that all members are adequately qualified, informed about the protocol, any amendments to the protocol, the trial treatments, and their trial-related duties and functions. Furthermore, he must nominate an adequately qualified deputy.

If the investigator/institution retains the services of any individual or party to perform trial-related duties and functions, the investigator/institution should ensure this individual or party is qualified to perform those trial-related duties and functions and should implement procedures to ensure the integrity of the trial-related duties and functions performed and any data generated

9.2 Quality of the source data

The investigator/institution should maintain adequate and accurate source documents and trial records that include all pertinent observations on each of the site's trial patients.

Source data should be attributable, legible, contemporaneous, original, accurate, and complete. Changes to source data should be traceable, should not obscure the original, and should be explained if necessary (e.g., via an audit trail).

When a copy is used to replace an original document, the copy has to fulfill the requirements for certified copies.

Data entered in the eCRF directly are listed in the monitoring plan in the section source data control.

9.3 Direct access to source data/documents

The investigator/institution must permit trial-related monitoring by the Interdisciplinary Center for Clinical Trials (IZKS) Mainz, as well as inspections by the appropriate competent authorities, and provide direct access to source data/documents (confidentiality see chapter 11.3).

The patients will be informed that representatives of the sponsor and/or competent authorities may inspect their medical records to verify the information collected and that all personal information made available for inspection will be handled in the strictest confidence and following local data protection laws.

9.4 Monitoring

Monitoring will be conducted according to the SOPs of the IZKS Mainz.

On-site monitoring will be done by personal visits from a clinical monitor. To initiate the trial, the monitor will visit all participating local trial sites. The monitor shall ensure that the investigators and members of the investigating staff understand all requirements of the protocol and their regulatory responsibilities.

The monitor will ensure that the investigator will maintain a list of members of the investigating staff to whom they have delegated significant trial-related duties ("delegation log").

Each site will be visited by the monitor at regular intervals to ensure compliance with the trial protocol, GCP, and legal aspects. The monitor will review the entries into the eCRF for completeness and correctness and verify the entries based on the source documents. The presence of correct informed consents will be checked for every patient. By frequent communications (letters, telephone, fax, meetings), the monitor will ensure that the trial is conducted according to the protocol and regulatory requirements.

The investigator must allow the monitor to look at all relevant documents and must provide support at all times to the monitor.

Details and the rationale for the chosen monitoring strategy will be specified in the monitoring plan for this trial.

9.5 Risk Management

During protocol development and the entire course of the trial, all potential and emerging risks (e.g. delayed eCRF data entry) to patient protection and reliability of the trial results will be closely monitored, and preventive and corrective measures will be specified. The sponsor will identify, evaluate, control, communicate, review, and report the relevant risks periodically to ensure continuous and timely risk management.

9.5.1 Potential COVID-19 related risks and mitigation strategies

Based on the currently literature, children below 12 years with current asthma do not appear to be predisposing factors for COVID (62, 63).

In case of another wave of the pandemic or other COVID-19-related restrictions (such as lockdown measures), follow-up visits can be performed remotely. In such cases, patients will be provided with enough IMP and relief medications to cover them through this period. If required, these relief medications can be delivered to the patients by the study site.

Patients will only be included in this study after the investigator has taken a detailed medical history and only if they fulfil all the eligibility criteria for the study. Further mitigation measures specific for COVID-19-related risks are outlined in section 9.5.2 below.

Some of the assessments during the study, such as spirometry measurements, may pose additional risks for SARS-CoV-2 infection (these are considered to be high-risk procedures as the maneuver require that patients expire forcefully which increases the risk of spreading the virus). COVID-19-related guidelines for conducting these types of procedures and strict infection control policies are available at the sites. Further mitigation measures for these high-risk procedures are outlined in the following section.

9.5.2 Further mitigation strategies for potential COVID-19 related risks

The following measures and provisions may minimize the risk of SARS-CoV-2 infection during the course of the study and must be adhered to:

1. Study sites should comply with standard infection control measures and applicable local guidelines and procedures (including site-specific measures) for COVID-19 to limit the risk of infection and transmission when performing study visits and assessments.
2. Study sites should ensure that adequate personal protective equipment (masks, gloves) is always available at the study site according to the available local guidelines (*e.g.,* the use of N95 masks for spirometry assessments in children aged ≥ 4 years).
3. For high-risk procedures, such as spirometry assessments, one should ensure that additional measures are implemented or reduce the risk of infection. Suitable measures are:
   - Performing these assessments in a well ventilated or isolated room, or both,
   - Using mouth-pieces with anti-microbial filters,
   - Using disposable transducers which are single-use only,
   - Using disposable nose clips.

Spirometry must not be done in patients with confirmed or suspected COVID-19.

4. Furthermore, testing for SARS-CoV-2 Rapid Antigen test may be performed prior to all visits where spirometry assessments will be done in applicable patients but only if this is required according to local guidelines and policies.
5. Caregivers should be instructed on the current recommendations for social distancing (according to local guidelines and policies) and other infection control measures to reduce the risk of infection and transmission.
6. Patients with caregivers will only be eligible for the study at screening and randomization if they do not have COVID-19-related symptoms and if they have had no contact with a confirmed COVID-19 case in the previous week.
7. At each study site visit (during all periods) and prior to IMP administration during the treatment period, the investigator will evaluate patients for symptoms of COVID-19 disease and whether the patient has had contact with a confirmed COVID-19 case in the previous week.
8. For unscheduled visits/assessments testing to diagnose SARS-CoV-2 infection, either the RT-PCR or the SARS-CoV-2 Rapid Antigen test should be performed. If this initial test is positive, the required period of isolation/quarantine should be observed as per local policies and guidelines.
9. Administration of the IMP may be permanently discontinued in patients who are hospitalized due to severe SARS-CoV-2 infection with ventilation.
10. If there is another wave of the pandemic prior to screening or during the study conduct requiring strict lockdown measures and which makes study conduct impossible (especially during the treatment period), a decision may be made to stop or postpone the study (*e.g.,* if dosing cannot be completed prior to the onset of the GPS in all or the majority of the patients).

The situation will be closely monitored by the IZKS. Furthermore, back-up sites in Germany may be included in the study if there are recruitment challenges arising due to local restrictions at sites or areas with active COVID-19 clusters.

9.6 Measures to secure compliance

Any significant non-compliance with the protocol, SOPs, GCP, and regulatory requirements by any party involved in the conduct of the trial will be analyzed (e.g. root cause analysis) by the sponsor, and appropriate corrective and preventive actions will be implemented.

9.7 Inspection by authorities

Competent authorities may request access to all source documents, eCRF, and other trial documentation in case of an inspection. Direct access to these documents must be guaranteed by the investigator who must provide support at all times for these activities. Source data documents can be copied during inspection, provided the identity of the patient has been made unrecognizable.

9.8 Audits

Persons (auditors) authorized by the sponsor may request access to all source documents, eCRF, and other trial documentation in case of an audit. Direct access to these documents must be

guaranteed by the investigator who must provide support at all times for these activities. Source data documents can be copied during audit, provided the identity of the patient has been made unrecognizable.

10 DATA MANAGEMENT

10.1 Responsibilities

The data management team is authorized in the case of discrepancies or correction of data errors to directly contact the responsible person at trial site. The queries will be sent by IZKS Mainz via email or will be directly entered into the eCRF.

A detailed methodology for the data management in this trial will be documented in a data management plan (DMP) that will be dated and maintained by IZKS Mainz. This plan has to be signed by the sponsor, the head of the data management team, and the responsible data manager. The document may modify the plans outlined in this protocol; however, any major modifications of the data handling will also be reflected in a protocol amendment.

10.2 Data collection

This trial will be performed using an electronic case report form (eCRF) or remote data entry (RDE). The investigator and the trial site staff will receive system documentation, training, and support for the use of the eCRF.

For support with data entry, IZKS Mainz can be contacted on **+49-6131–179920** or **+49–6131-179922.**

All protocol-required information collected during the trial must be entered by the investigator or a designated member of the investigating team in the eCRF. All data entries, modifications, or deletions will be recorded automatically in an electronic audit trail indicating the individual patient, the original and new values, the reason for and time and date of change, as well as the person executing the change.

The system will be secured to prevent unauthorized access to the data or the system. Only people provided with a user ID and a password will be able to enter or change data. The investigator will maintain a list of individuals who are authorized to enter or correct data and their system ID.

Computer hardware and software (for accessing the data) will be maintained at or made available for the site in compliance with applicable regulations. All technical preconditions for each trial site are recorded in the DMP.

The system is capable of producing exact copies of data in legible paper form for inspections and audits. The investigator or a designated member of the investigating team, following review of the data in the eCRF, will confirm the validity of each patient's data by electronic signature or by signing a paper printout of a listing of all patients enrolled in the trial.

The architecture of the computer system will be described in the DMP.

10.3 Data handling

During data entry, integrity checks help to minimize entry failures. These data entry checks are based on the data validation plan (DVP), signed by the sponsor (or a sponsor representative). The data entry system allows the trial monitors and data managers to control the entry process with the help of built-in review functions. Comments and requests can be promptly processed by the trial site.

Checks for plausibility, consistency, and completeness of the data will be performed during data entry. Based on these checks, queries will be produced. Any missing data or inconsistencies will be reported back to the respective site and clarified by the responsible investigator.

After completion of data entry and if no further corrections are to be made in the database, the access rights will be withdrawn and the database will be declared closed and ready to be used for statistical analysis.

All data management activities will be done according to the current Standard Operating Procedures (SOPs) of IZKS Mainz.

10.4 Storage and archiving of data

The investigator will be provided with an Investigator Site File (ISF) at the start of the trial. The investigator will archive all trial data and relevant correspondence in the ISF. The ISF, all source data, and all essential documents will be kept filed according to applicable legal regulations (esp. § 13, section 10 GCP regulation) and to the ICH-GCP guidelines after termination of the trial.

The sponsor and investigator/institution should maintain a record of the location(s) of their respective essential documents including source documents. The storage system used during the trial and for archiving (irrespective of the type of media used) should provide for document identification, version history, search, and retrieval.

The proper and GCP conform storage and archiving of the electronic data during the trial will be ensured by IZKS Mainz. After completion of the trial, all electronic data will be handed over to the sponsor.

11 ETHICAL AND LEGAL ASPECTS

11.1 Good clinical practice

The trial will be conducted according to the principles of ICH-GCP and legal regulations (German AMG, GCP regulation). The Declaration of Helsinki in its latest accepted version in Germany will be adhered to. The study protocol and substantial amendments will be approved by the responsible ethics committees and competent authorities before the start of the trial. Before being admitted to the clinical trial, the patient must give written consent to participate after the nature, scope, and possible consequences of the clinical trial have been explained in a form understandable to him or her. The procedures set out in this trial protocol will be performed according to the worldwide recognized principles of good clinical practice (GCP; as defined in the ICH-E6 R2 Guideline) and in compliance with the ethical principles described in the current version of the Declaration of Helsinki.

11.2 Patient information and informed consent

In general, patients and parents/guardians should be informed to the fullest extent possible about the study in a way they are able to understand. The investigator informs at least the parents/guardians about the nature, importance, risks, and individual consequences of the clinical trial and their right, to terminate the participation at any time.

Since the patients in this trial are minors, the consent of the legally authorized representatives[1] must be sought. Whenever appropriate, the child should participate in the (informed) consent process together with the parents/legal guardians. Therefore, when the child is able to give assent to participation in the study, that assent will additionally be obtained by the investigator. The ability to give assent will be evaluated according to age and level of maturity.

The consent must be confirmed by the personally dated signature of the representative(s) and the personally dated signature of the physician conducting the informed consent discussion.

Written informed consent must be obtained from either one or both parents/guardians who have the legal authority to make healthcare decisions for the child, according to local or country legal requirements. If either mother or father (or one legal guardian) has sole legal custody of the study child, this needs to be confirmed by a respective official document.

If one parent/legal guardian agrees but the other does not, the child must not be enrolled in the study. Where the parent/legal guardian agrees to consent but the child dissents, the wishes of the child shall prevail.

The written patient information must be in a language understandable to the parents/guardians and must specify who informed the parents/guardians. The parents/guardians should also have the opportunity to consult the investigator or a physician member of the investigating team about the details of the clinical trial. The informed consent to participate in the clinical trial may be withdrawn by the parents/guardians patient verbally in the presence of, or in written form directed to, the investigator or a physician member of the investigating team at any time during the trial. The patient must not entail any disadvantage therefore or be coerced or unduly influenced to continue to participate. Furthermore, the parents/guardians are not obligated to disclose reasons for the withdrawal of the consent.

The investigator must not undertake any measures specifically required for the clinical trial until valid consent has been obtained.

If the patient has a primary physician, the investigator should inform him or her about the patient's participation in the trial, provided the parents/guardians agree hereto.

The patient (where appropriate) and the patient's parents (or legal guardians) must be informed that the patient's trial-related data will be used in accordance with the local data protection law.

The level of disclosure must also be explained to his/her parents (or legal guardians). The patient's parents (or legal guardian) must be informed that the patient's medical records may be examined by monitors by appropriate IRB/IEC members, and by inspectors from regulatory authorities.

[1] "Legally authorized representative" means an individual or judicial or other body authorized under applicable law to consent on behalf of a prospective patient to the patient's participation in the trial procedure(s). In case of minors, all persons having the care and custody of the minor have to be informed and must consent.

Consenting are dynamic and continuous processes that should be maintained during the trial. Site staff should document this process in the medical records at each visit. The documentation should confirm that the study was discussed during the visit and that the date for the next clinical trial visit was agreed with the parent(s)/legal guardian.

Best practice should be followed that consent is not just a signature at the beginning of the study but site staff must verbally reiterate key messages throughout the study and ensure parents/guardians are still happy for child participation at each study visit.

11.3 Confidentiality

The names of the patients and other confidential information are subject to medical professional discretion and the regulations of the German laws on data protection (Federal Data Protection Act; German: Bundesdatenschutzgesetz, BDSG). The name of the patients and other confidential information will not be disclosed to the sponsor.

During the clinical trial, patients will be identified solely using an individual identification code (e.g. patient number, randomization number). The investigator will maintain a personal patient identification list (patient numbers with the corresponding patient names) to enable records to be identified.

Trial data (electronic and in paper form) will be handled in the strictest confidence. Security procedures will be implemented to prevent disclosure of data to unauthorized persons. The appropriate regulations of data legislation will be fulfilled in its entirety.

The patient will declare in the written consent to release the investigator from the medical professional discretion to enable the attribution of the trial data in case of inspections by health authorities, audits by the sponsor and data monitoring by authorized sponsor representatives (monitors).

11.4 Responsibilities of the investigator

In accordance with § 67, section 1 AMG and § 12, section 1 GCP regulation this clinical trial will be notified to the local competent authority responsible for the investigator as well as to the higher federal authority (BfArM) by the IZKS Mainz.

Furthermore, the local authorities responsible for the respective trial sites will be notified within 90 days after regular completion of the trial by the IZKS Mainz. If the trial has been terminated prematurely, the notification has to be carried out within 15 days and has to state the reason for trial discontinuation (§ 12, section 2 GCP regulation).

The responsibilities of the investigator with regard to documentation and reporting of AEs according to § 12, section 4-7 GCP regulation are described in section 7.

The investigator nominates adequately qualified members of the investigating team (§ 40 section 1a AMG) and must instruct and supervise them to ensure that they are adequately informed about relevant information regarding the trial, especially the trial protocol and investigators brochure. Furthermore, he has to nominate a deputy with qualifications comparable to his own. Replacements of the investigator or his deputy, as well as substantial changes of the trial site qualification, have to be approved by the local ethics committee.

The investigator will maintain a list of the members of the investigating team and other persons to whom they have delegated significant trial-related duties (“delegation log”).

11.5 Approval of trial protocol and amendments

11.5.1 Submissions

Before the start of the trial, the sponsor submits a written application for trial approval to the competent authority as well as for the favorable opinion of the ethics committee, responsible for the coordinating investigator. Local ethics committees, responsible for the other trial sites receive a copy of the application. Additionally, the local ethics committees receive detailed information on the respective trial sites and the investigators to confirm their qualification.

Trial sites joining the ongoing trial have to undergo the same approval procedure at their ethics committees.

Investigational products can only be supplied to the trial sites after the fulfillment of all ethical and legal requirements for initiation (e.g. needed approvals are granted) of the clinical trial has been confirmed by IZKS Mainz on behalf of the sponsor (“regulatory greenlight”).

11.5.2 Amendments of protocol

Neither the investigator nor the sponsor may alter this trial protocol without obtaining the written agreement of the other party. Subsequent changes to the protocol during an ongoing trial have to be implemented via protocol amendments. The sponsor is responsible for obtaining the approval for the amendments by the ethics committee and/or by the competent authority depending on the provisions of § 10, section 1 GCP regulation.

11.6 Other information to ethics committees and competent authorities

The sponsor is responsible for the ongoing evaluation of the safety of the IMP and the participants of the trial; the corresponding notifications in accordance with § 11 and § 13 GCP regulation are described in chapter 7 (“Safety”).

11.7 Documentation of Correspondence

Relevant correspondence with competent authorities and ethics committees will be properly archived by the sponsor or IZKS Mainz on behalf of the sponsor.

11.8 Data monitoring committee (DMC)

The independent data safety and monitoring board (DSMB) will supervise the conduct of this trial and will issue recommendations for early termination, modifications, or continuation of the trial according to the DSMB operating procedure.

11.9 Insurance

According to § 40 AMG, the sponsor (or a designated representative of sponsor) has effected an insurance policy covering his legal liability for injuries caused to participating persons and arising out of the trial procedures (limited to € 500.000,- per participating person).

The insurance was taken out at **AXA XL, insurance number: DE00048297-LI-21A-712-** (maximum limit: 500.000,00 € per participating person).

Any impairment of health that might occur in consequence of trial participation must be immediately reported to the insurance company by the patient concerned; if applicable, after consulting the investigator. The insured person will consent to and comply with all appropriate measures serving for clarification of the cause and the extent of damage as well as the reduction of damage.

During the conduct of the trial, the patient must not undergo other clinical treatment except in cases of emergency. The patient is obliged to inform the investigator immediately about any emergency treatments, AEs, and additional medication taken. The terms and conditions of the insurance should be given to the patient.

11.10 Agreements

11.10.1 Financing of the trial

The trial is funded by the Federal Ministry for Education and Research (German: BMBF). The general conditions of financing for this trial are addressed in separate agreements.

11.10.2 Report

After conclusion of the trial, the sponsor ensures that a report shall be written according to the conditions stipulated in the protocol by. By signing this protocol the investigators agree to disclose their names in the trial report according to § 42b AMG.

11.10.3 Publication policy

Any publication of the results, either in part or in total (articles in journals or newspapers, oral presentation, etc.) by the investigators, their representatives, or by the sponsor, shall require the approval of the coordinating investigator.

It is planned to publish the results of the trial as an original article in an appropriate medical journal as well as present at congresses. The coordinating investigator is last author of the article and will present the data at major congresses. The choice of the journal for the publication will be made by the coordinating investigator in agreement with the co-authors. Besides the investigator, further authors of this article have to meet the following criteria:

- Substantial contribution to the recruitment of patients, i.e. one of the five best-recruiting sites within the trial.
- Substantial contribution to the interpretation of the data.
- Substantial contribution to drafting the article or revising it critically for important intellectual content.

12 SIGNATURES

The present trial protocol was subject to critical review and has been approved in the present version by the persons undersigned. The information contained is consistent with:

- The current risk-benefit assessment of the IMP.
- The moral, ethical, and scientific principles governing clinical research as set out in the Declaration of Helsinki and the principles of GCP.

Operating institution of Sponsor

Name:

Date Signature

Coordinating Investigator

Name: [redacted]

11.09.2023 [redacted]

Date Signature

Biometrician

Name: [redacted]

06.09.2023 [redacted]

Date Signature

13 DECLARATION OF INVESTIGATOR

I have read the above trial protocol and I confirm that it contains all information to accordingly conduct the clinical trial. I pledge to conduct the clinical trial according to the protocol.

I will enroll the first patient only after all ethical and regulatory requirements are fulfilled. I pledge to obtain written consent for trial participation from all patients.

I know the requirements for accurate notification of serious adverse events and I pledge to document and notify such events as described in the protocol.

I pledge to retain all trial-related documents and source data as described. I will provide a Curriculum Vitae (CV) before trial start. I agree that the CV may be submitted to the responsible competent authorities.

I will conduct the trial in compliance with the protocol, GCP, and the applicable regulatory requirements.

Investigator

Name
Address
Phone
Fax
email

__________________ ____

Date Signature

14 REFERENCES

1. Ferrante G, La Grutta S. The burden of pediatric asthma. Front Pediatr. 2018; 6: 186.
2. European Respiratory Society. European Lung white book – Chapter 11 Childhood asthma. https://www.erswhitebook.org/chapters/childhood-asthma/. Accessed November 15, 2019.
3. Weber A, Herr C, Hendrowarsito L, et al. No further increase in the parent reported prevalence of allergies in Bavarian preschool children: Results from three cross-sectional studies. Int J Hyg Environ Health. 2016; 219(4): 343-348.
4. Liu AH, Gilsenan AW, Stanford RH, Lincourt W, Ziemiecki R, Ortega H. Status of asthma control in pediatric primary care: results from the pediatric asthma control characteristics and prevalence survey study (ACCESS) J Pediatr. 2010;157:276–81.
5. Guilbert TW, Garris C, Jhingran P, et al. Asthma that is not well-controlled is associated with increased healthcare utilization and decreased quality of life. J Asthma. 2011; 48:126–32.
6. Haselkorn T, Fish JE, Zeiger RS, et al. Consistently very poorly controlled asthma, as defined by the impairment domain of the Expert Panel Report 3 guidelines, increases risk for future severe asthma exacerbations in The Epidemiology and Natural History of Asthma: Outcomes and Treatment Regimens (TENOR) study. J Allergy Clin Immunol 2009;124:895–902.e4.
7. Pedersen SE, Hurd SS, Lemanske RF, Becker A, Zar HJ, Sly PD, Soto-Quiroz M, Wong G, Bateman ED. Global strategy for the diagnosis and management of asthma in children 5 years and younger. Pediatr Pulmonol 2011, 46 (1), 1 -17.
8. Bisgaard H, Szefler S. Prevalence of asthma-like symptoms in young children. Pediatr Pulmonol. 2007; 42, 723–728.
9. Martinez FD, Wright AL, Taussig LM, Holberg CJ, Halonen M, Morgan WJ. Asthma and wheezing in the first six years of life. N Engl J Med 1995; 332; 113-82.
10. Sly PD, Boner AL, Bjorksten B, Bush A, Custovic A, Eigenmann PA, Gern JE, Gerritsen J, Hamelmann E, Helms PJ, Lemanske RF, Martinez F, Pedersen S, Renz H, Sampson H, Mutius E von, Wahn U, Holt PG. Early identification of atopy in the prediction of persistent asthma in children. Lancet 2008; 372, 1100 – 1106.
11. Carlsen KH. What distinguishes the asthmatic amongst the infant wheezers? Pediatr Allergy Immunol 1997; 8; S 40-45.
12. Kurukulaaratchy RJ, Matthews S, Holgate ST, Arshad SH. Predicting persistent disease among children who wheeze during early life. Eur Respir J. 2003;22(5):767-71.
13. Yuzaburo Inoue, Naoki Shimojo. Epidemiology of virus-induced wheezing/asthma in children. Front Microbiol. 2013; 4: 391.
14. Akinbami L, Moorman J, Garbe P, Sondik E. Status of Childhood Asthma in the United States, 1980–2007. Pediatrics. 2009;123:S131–S45.
15. Akinbami L. The state of childhood asthma, United States, 1980–2005. Adv Data. 2006:1–24.
16. Beigelman A, Bacharier LB. Management of preschool recurrent wheezing and asthma: a phenotype-based approach. Curr Opin Allergy Clin Immunol. 2017;17(2):131-138.
17. James C. Hogg, Peter D. Paré, and Tillie-Louise Hackett.The Contribution of Small Airway Obstruction to the Pathogenesis of Chronic Obstructive Pulmonary Disease. Physiol Rev. 2017; 97(2): 529–552.
18. Donath H, Kluge S, Sideri G, et al. Hospitalization, asthma phenotypes, and readmission rates in pre-school asthma. Front Pediatr 2020 Nov 20;8:562843.
19. Kerkhof M, Tran TN, van den Berge M, et al. Association between blood eosinophil count and risk of readmission for patients with asthma: Historical cohort study. PLoS One. 2018 25;13(7):e0201143.
20. Delmas MC, Marguet C, Raherison C, Nicolau J, Fuhrman C. Readmissions for asthma in France in 2002–2005. Rev Mal Respir. 2011;28:e115–22. 10.1016.
21. Laitinen LA, Altraja A, Karjalainen EM et al. Early interventions in asthma with inhaled corticosteroids. J Allergy Clin Immunol 2000; 105(2 Pt 2):S582-5.

22. Pauwels RA, Pedersen S, Busse WW, Tan WC, Chen YZ, Ohlsson SV, et al. Early intervention with budesonide in mild persistent asthma: a randomised, double-blind trial. Lancet. 2003 29;361(9363):1071-6.
23. Fitzpatrick AM, Bacharier LB, Guilbert TW, et al. Phenotypes of recurrent wheezing in preschool children: Identification by latent class analysis and utility in prediction of future exacerbation. J Allergy Clin Immunol Pract. 2019;7(3):915-924.
24. Bisgaard H, Zielen S, Garcia-Garcia ML, et al.. Montelukast reduces asthma exacerbations in 2- to 5-year-old children with intermittent asthma. Am J Respir Crit Care Med. 2005 15; 171(4):315-22.
25. Zielen S, Christmann M, Kloska M, et al. Predicting short term response to anti-inflammatory therapy in young children with asthma. Curr Med Res Opin. 2010; 26(2):483-92.
26. Wassermann RL, Baker JW, Kim KT. Efficacy and safety of inhaled fluticasone propionate chlorfluorocarbon in 2- to 4-year-old patients with asthma: results of a double-blind, placebo-controlled study. Ann Allergy Asthma Immunol. 2006; 96(6):808-18.8.
27. Kaiser SV, Huynh T, Bacharier LB, et al. Preventing Exacerbations in Preschoolers With Recurrent Wheeze: A Meta-analysis. Pediatrics. 2016:137.
28. Jackson DJ, Bacharier LB, Mauger DT, et al. Quintupling Inhaled Glucocorticoids to Prevent Childhood Asthma Exacerbations. N Engl J Med. 2018 8; 378(10):891-901.
29. Chauhan BF, Chartrand C, Ni Chroinin M, Milan SJ, Ducharme FM. Addition of long-acting beta2-agonists to inhaled corticosteroids for chronic asthma in children. Cochrane Database Syst Rev. 2015 24;(11):
30. Yoshihara S, Tsubaki T, Ikeda M, et al. The efficacy and safety of fluticasone/salmeterol compared to fluticasone in children younger than four years of age. Pediatr Allergy Immunol. 2019; 30(2):195-203.
31. Beck R, Robertson C, Galdes-Sebaldt M, Levison H. Combined salbutamol and ipratropium bromide by inhalation in the treatment of severe acute asthma. J Pediatr 1985; 107, 605– 608.
32. Hamelmann E, Bateman ED, Vogelberg C, et al. Tiotropium add-on therapy in adolescents with moderate asthma: a 1-year randomized controlled trial. J Allergy Clin Immunol. 2016;138(2):441–450.
33. Hamelmann E, Bernstein JA, Vandewalker M, et al. A randomised controlled trial of tiotropium in adolescents with severe symptomatic asthma. Eur Respir J. 2017;49(1):1601100.
34. Vogelberg C, Engel M, Laki I, et al. Tiotropium add-on therapy improves lung function in children with symptomatic moderate asthma. J Allergy Clin Immunol Pract. 2018; 6(6):2160–2162.
35. Szefler SJ, Murphy K, Harper T, et al. A phase III randomized controlled trial of tiotropium add-on therapy in children with severe symptomatic asthma. J Allergy Clin Immunol. 2017;140(5):1277–1287.
36. Vogelberg C, Szefler SJ, Vrijlandt EJLE et al. Tiotropium add-on therapy is safe and reduces seasonal worsenings in paediatric asthma patients. Eur Respir J. 2019 13;53(6):1801824.
37. Global Initiative for Asthma. Global Strategy for asthma management and prevention (2019 update). Available from:https://ginasthma.org/wp-content/uploads/2019/06/GINA-2019-main-report-June-2019-wms.pdf. Last accessed June 2019.
38. Ohta S, Oda N, Yokoe T, et al. Effect of Tiotropium bromide on airway inflammation and remodelling in a mouse model of asthma. Clin Exp Allergy. 2010; 40:1266–75.
39. Tashkin DP, Dahl R, Virchow JC, et al. Once-daily Tiotropium respimat® add-on to at least ICS Maintenance therapy demonstrates improved lung function in patients with symptomatic asthma, in dependent of serum IgE or blood Eosinophil levels. J Allergy Clin Immunol. 2016; 12, 23.
40. Casale TB, Bateman ED, Vandewalker M, Virchow JC, Schmidt H, Engel M, et al. Tiotropium Respimat add-on is efficacious in symptomatic asthma, independent of T2 phenotype. JAllergy Clin Immunol Pract. 2018; 6(3):923-935.e9.

41. Buhl R, Hamelmann E. Future perspectives of anticholinergics for the treatment of asthma in adults and children. Ther Clin Risk Manag. 2019 14;15:473-485.
42. Vrijlandt E, El Azzi G, Vandewalker M, et al. Safety and efficacy of Tiotropium in children aged 1–5 years with persistent asthmatic symptoms: a randomised, double-blind, placebo-controlled trial. Lancet Respir Med. 2018; 6(2):127–137.
43. Zielen S, G. Reichert, H. Donath et al. Tiotropium as an add-on treatment option for severe uncontrolled asthma in preschool patients. J Asthma Allergy 2021 Jan 14;14:23-30
44. Mansfield L, Duong-Quy S, Craig T.Burden of Asthma and Role of 2.5 µg Tiotropium Respimat® as an add-on therapy: A systematic review of phase 2/3 trials. Adv Ther. 2019 ;36 (10):2587-2599.
45. Kamin W, Frank M, Kattenbeck S, Moroni-Zentgraf P, Wachtel H, Zielen S. A Handling Study to Assess Use of the Respimat(®) Soft mist inhaler in children under 5 years Old. J Aerosol Med Pulm Drug Deliv. 2015; 28(5):372-81.
46. Bickmann D, Kamin W, Sharma A, Wachtel H, Moroni-Zentgraf P, Zielen S. In Vitro Determination of Respimat® Dose Delivery in Children: An Evaluation Based on Inhalation Flow Profiles and Mouth-Throat Models. J Aerosol Med Pulm Drug Deliv. 2016; 29(1):76-85.
47. Bateman ED, Kornmann O, Schmidt P, Pivovarova A, Engel M, Fabbri LM. Tiotropium is noninferior to sameterol in maintaining improved lung function in B16-Arg/Arg patients with asthma J Allergy Clin Immunol, 2011; 128: 315-322.
48. Spiriva® Respimat® Product Monograph Page 1-41 Boehringer Ingelheim (Canada) Ltd. 5180 South Service Road Burlington, Ontario. L7L 5H4 version Mar 2020.
49. Dusser D, Bravo ML, Iacono P, MISTRAL study group. The effect of Tiotropium on exacerbations and airflow in patients with COPD. Eur Respir J 2006; 27 (3), 547–555.
50. Tashkin DP, Celli B, Senn S, Burkhart D, Kesten S, Menjoge S, Decramer M, UPLIFT Study Investigators. A 4-year trial of Tiotropium in chronic obstructive pulmonary disease. N Engl J Med 2008; 359 (15), 1543–1554.
51 O'Connor BJ, Towse LJ, Barnes PJ. Prolonged effect of Tiotropium bromide on methacholine-induced bronchoconstriction in asthma. Am J Respir Crit Care Med. 1996; 154(4 Pt 1):876-80.
52. Meng JF, Li H, Luo MJ, Li HB. Efficacy of Tiotropium in treating patients with moderate-to-severe asthma. A meta-analysis and systematic review based on 14 randomized controlled trials. Medicine (Baltimore). 2019; 98(33):e16637.
53. Vogelberg C, Moroni-Zentgraf P, Leonaviciute-Klimantaviciene M, Sigmund R, Hamelmann E, Engel M, Szefler S. A randomised dose-ranging study of Tiotropium Respimat® in children with symptomatic asthma despite inhaled corticosteroids. Respir Res. 2015; 16(1): 20.
54. Santanello NC, DeMuro-Mercon C, Davies G, Ostrom N, Noonan M, Rooklin A, Knorr B. Validation of a pediatric asthma caregiver diary. J Allergy Clin Immunol 2000; 106 (5), 861–866.
55. Chipps B, Zeiger RS, Murphy K,et al. Longitudinal validation of the Test for Respiratory and Asthma Control in Kids in pediatric practices. Pediatrics . 2011;127(3):e737-47
56. Beydon N, Beydon N, Davis SD, Lombardi E, Allen JL, Arets HG, Aurora P, et al. An official American Thoracic Society/European Respiratory Society statement: pulmonary function testing in preschool children. Am J Respir Crit Care Med 2007;175, 1304–1345.
57. Christmann M, Erffa Sv, Rosewich M, Rose MA, Schulze J, Zielen S. [The repeatability of forced expiratory ma- noeuvres in 4- to 6-year-old children with intermittent bronchial asthma in healthy and in exacerbated status].Pneumologie. 2010; 64(12):745-51.
58. Schulze J, Biedebach S, Christmann M, Herrmann E, Voss S, Zielen S. Impulse oscillometry as a predictor of asthma exacerbations in young children. Respiration 2016; 91 (2): 107-14.
59. Smith H-J in Rühle K. Special lung function diagnostics: implementation and interpretation of oscillometry: Dustri-Verlag Feistle; 2013.
60. Galant SP, Komarov HD, Shin H, Siddiqui S, Lipworth BJ. The case for impulse oscillometry in the management of asthma in children and adults. Ann Allergy Asthma Immunol 2017;

118 (6): 664-71.
61. Knihtilä H, Kotaniemi-Syrjänen A, Pelkonen AS, Kalliola S, Mäkelä MJ, Malmberg LP. Small airway oscillometry indices: Repeatability and bronchodilator responsiveness in young children. Pediatr Pulmonol 2017; 52 (10): 1260-1265.
62. Dong Y, Mo X, Hu Y, Qi X, Jiang F, Jiang Z, Tong S. Epidemiology of COVID-19 among children in China. Pediatrics. 2020;145(6):e20200702.
63. Donath H, Zielen S, Wittekindt B, et al. Effects of the SARS-CoV2-Lockdown on Pediatric Care in the Rhine-Main Area. Klin Padiatr. 2021;233(1):31-36.
64. SMPC Spiriva Respimat version Mar 2018
65. Lambert KA, Roff AN, Panganiban RP, Douglas S, Ishmael FT. MicroRNA-146a is induced by inflammatory stimuli in airway epithelial cells and augments the anti-inflammatory effects of glucocorticoids. PLoS One. 2018;13(10):e0205434. Published 2018 Oct 9. doi:10.1371/journal.pone.0205434
66. Yan F, Meng W, Ye S, et al. MicroRNA146a as a potential regulator involved in the pathogenesis of atopic dermatitis. Mol Med Rep. 2019;20(5):4645-4653. doi:10.3892/mmr.2019.10695
67 Hammad Mahmoud Hammad R, Hamed DHED, Eldosoky MAER, et al. Plasma microRNA-21, microRNA-146a and IL-13 expression in asthmatic children. Innate Immun. 2018;24(3):171-179. doi:10.1177/1753425918763521
68. Wu XB, Wang MY, Zhu HY, et al. Overexpression of microRNA-21 and microRNA-126 in patients of bronchial asthma. Int J Clin Exp Med 2014; 7: 1307-1312.
69. Lu TX, Munitz A, Rothenberg ME. MicroRNA-21 is up-regulated in allergic airway inflammation and regulates IL-12p35 expression. J Immunol 2009; 182: 4994-5002.
70. Davis JS, Sun M, Kho AT, Moore KG, Sylvia JM, Weiss ST, Lu Q, Tantisira KG. Circulating microRNAs and association with methacholine PC20 in the Childhood Asthma Management Program (CAMP) cohort. PLoS One. 2017 Jul 27;12(7):e0180329. doi: 10.1371/journal.pone.0180329. PMID: 28749975; PMCID: PMC5531511.
71. Patel MN, Bernard WG, Milev NB, Cawthorn WP, Figg N, Hart D, et al.Hematopoietic IKBKE limits the chronicity of inflammasome priming and metaflammation.Proceedings of the National Academy of Sciences of the United States of America. 2015;112(2):506-11. doi:10.1073/pnas.1414536112;
72. Sheller JR, Polosukhin VV, Mitchell D, Cheng DS, Peebles RS, Blackwell TS.Nuclear factor kappa B induction in airway epithelium increases lung inflammation in allergen-challenged mice. Experimental lung research. 2009;35(10):883-95. doi:10.3109/01902140903019710
73. Ziegelbauer K, Gantner F, Lukacs NW, Berlin A, Fuchikami K, Niki T, et al.A selective novel low-molecular-weight inhibitor of IkappaB kinase-beta (IKK-beta) prevents pulmonary inflammation and shows broad anti-inflammatory activity.British journal of pharmacology. 2005;145(2):178-92. doi:10.1038/sj.bjp.0706176

15 APPENDICES

15.1 Additional information regarding inclusion/exclusion criteria

Levels of asthma control in children 5 years and younger

Table 3: *Levels of asthma control as defined by GINA (7)*

Characteristic	Controlled (all of the following)	Partly controlled (any measure present in any week)	Uncontrolled (three or more of features of partly controlled asthma in any week)
Daytime symptoms: wheezing, cough, difficult breathing	None (less than twice/week, typically for short periods on the order of minutes and rapidly relieved by use of a rapid-acting bronchodilator)	More than twice/week (typically for short periods on the order of minutes and rapidly relieved by use of a rapid-acting bronchodilator)	More than twice/week (typically last minutes or hours or recur, but partially or fully relieved with rapid-acting bronchodilator)
Limitations of activities	None (child is fully active, plays and runs without limitation or symptoms)	Any (may cough, wheeze, or have difficulty breathing during exercise, vigorous play, or laughing)	Any (may cough, wheeze, or have difficulty breathing during exercise, vigorous play, or laughing)
Nocturnal symptoms/ awakening	None (including no nocturnal coughing during sleep)	Any (typically coughs during sleep or wakes with cough, wheezing, and/ or difficult breathing)	Any (typically coughs during sleep or wakes with cough, wheezing, and/or difficult breathing
Need for reliever/ rescue treatment	≤2 days/week	>2 days/week	>2 days/week

This scheme (based on current expert opinion) presents characteristics of controlled, partly controlled, and uncontrolled asthma for children 5 years and younger based on (1) symptoms recognized by family members/caregivers and (2) the child's need for reliever/rescue treatment.

15.2 Dosing table systemic corticosteroids

Table 4: *Dosing recommendations for systemic corticosteroids (Celestamine®)*

Age of patients	Betametasone (mg)	Betametasone (ml)
1-2 years	Twice daily 1.0-1.0 mg	Twice daily 2.0-2.0 ml
2-3 years	Twice daily 1.25-1.25 mg	Twice daily 2.5-2.5 ml
3-4 years	Twice daily 1.5-1.5 mg	Twice daily 3.0-3.0 ml
3-5 years	Twice daily 2.0-2.0 mg	Twice daily 4.0-4.0 ml

This dose of Betametasone should be given for 3 days during a severe exarcerbation

15.3 Instructions for the use of the Respimat® inhaler

Instructions for Use

Respimat® inhaler

How to use your Respimat® inhaler

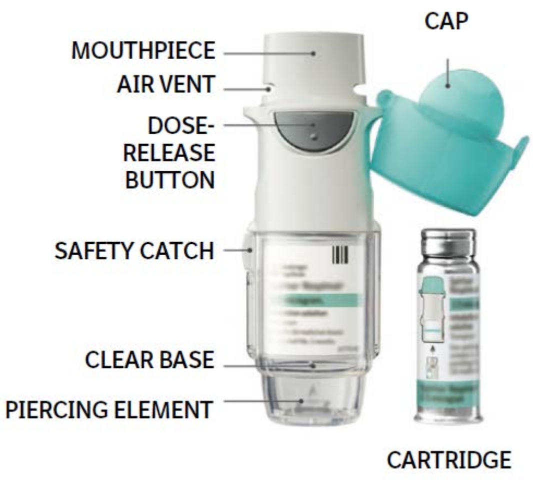

- If Spiriva Respimat has not been used for more than 7 days release one puff towards the ground.
- If Spiriva Respimat has not been used for more than 21 days repeat steps 4 to 6 under ‘Prepare for first use’ until a cloud is visible. Then repeat steps 4 to 6 three more times.
- Do not touch the piercing element inside the clear base.

How to care for your Spiriva Respimat

Clean the mouthpiece including the metal part inside the mouthpiece with a damp cloth or tissue only, at least once a week. Any minor discoloration in the mouthpiece does not affect your Spiriva Respimat inhaler performance. If necessary, wipe the outside of your Spiriva Respimat inhaler with a damp cloth.

When to get a new Spiriva Respimat

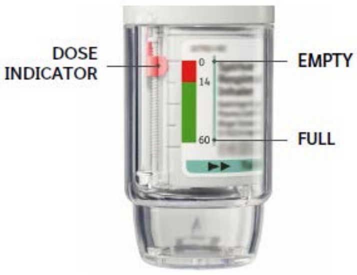

- Your Spiriva Respimat inhaler contains 60 puffs (30 doses) if used as indicated (two puffs/once daily).
- The dose indicator shows approximately how much medication is left.
- When the dose indicator enters the red area of the scale you need to get a new prescription; there is approximately medication for 7 days left (14 puffs).
- Once the dose indicator reaches the end of the red scale, your Spiriva Respimat locks automatically – no more doses can be released. At this point, the clear base cannot be turned any further.

- Spiriva Respimat should be discarded three months after you have prepared it for first use, even if it has not been fully used or used at all.

Prepare for first use

<table>
<tr><td>1. Remove clear base
• Keep the cap closed.
• Press the safety catch while firmly pulling off the clear base with your other hand.</td><td>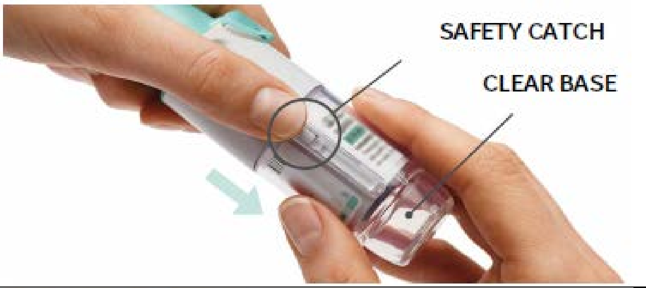
</td></tr>
<tr><td>2. Insert cartridge
• Insert the narrow end of the cartridge into the inhaler.
• Place the inhaler on a firm surface and push down firmly until it clicks into place.
• Do not remove the cartridge once it has been inserted into the inhaler.</td><td>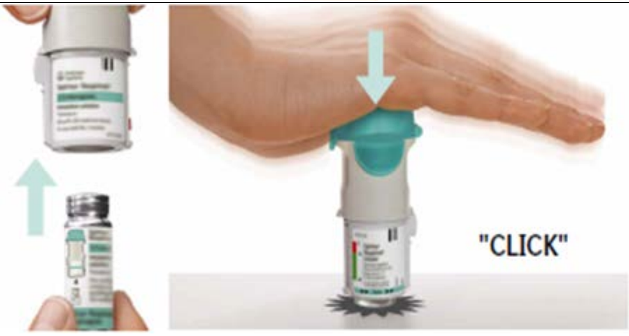
</td></tr>
<tr><td>3. Replace clear base
• Put the clear base back into place until it clicks.
• Do not remove the clear base again.</td><td>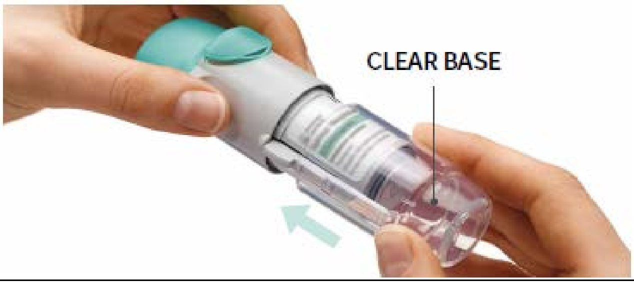
</td></tr>
</table>

<table>
<tr><td>

4. Turn

- Keep the cap closed.
- Turn the clear base in the direction of the arrows on the label until it clicks (half a turn).

</td><td>

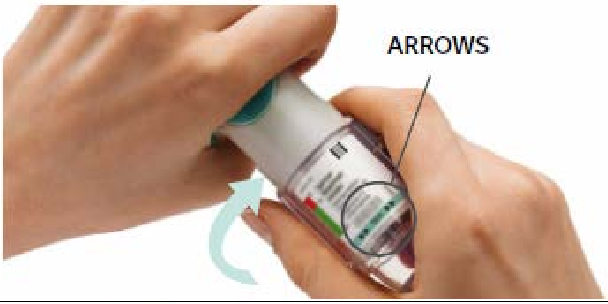

</td></tr>
<tr><td>

5. Open

- Open the cap until it snaps fully open.

</td><td>

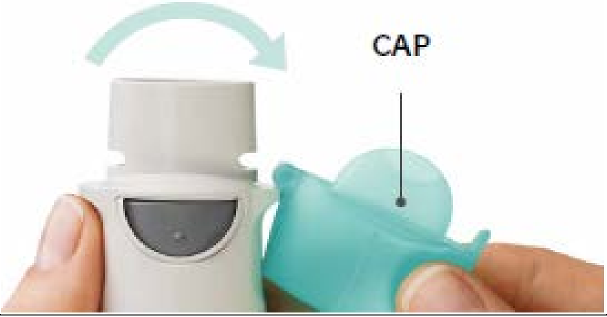

</td></tr>
<tr><td>

6. Press

- Point the inhaler toward the ground.
- Press the dose-release button.
- Close the cap.
- Repeat steps 4-6 until a cloud is visible.
- **After a cloud is visible**, repeat steps 4-6 three more times.

Your inhaler is now ready to use. These steps will not affect the number of doses available. After preparation your inhaler will be able to deliver 60 puffs (30 doses).

</td><td>

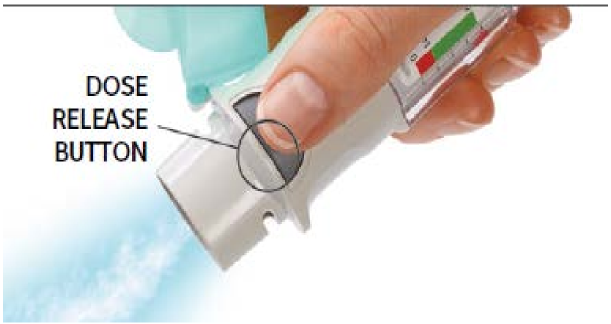

</td></tr>
</table>

Daily use

**TURN**<br>• Keep the cap closed.<br>• **TURN** the clear base in the direction of the arrows on the label until it clicks (half a turn).	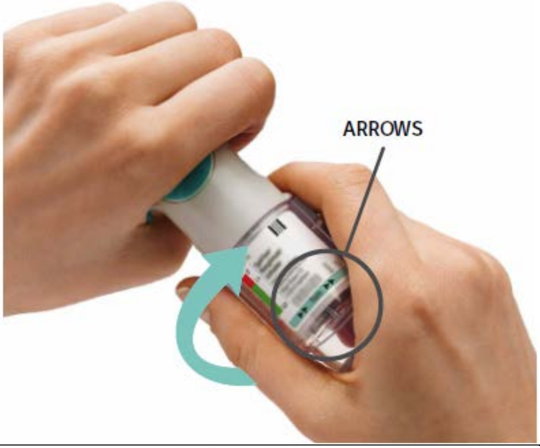
**OPEN**<br>• **OPEN** the cap until it snaps fully open.	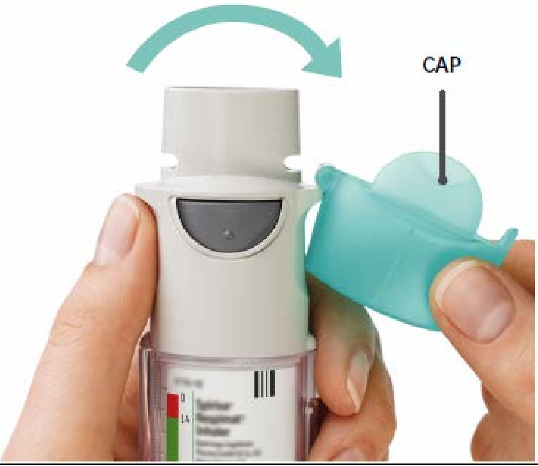
**PRESS**<br>• Breathe out slowly and fully.<br>• Close your lips around the mouthpiece without covering the air vents. Point your Inhaler to the back of your throat.<br>• While taking a slow, deep breath through your mouth, **PRESS** the dose-release button and continue to breathe in slowly for as long as comfortable.<br>• Hold your breath for 10 seconds or for as long as comfortable.<br>• Repeat Turn, Open, Press for a total of 2 puffs.<br>• Close the cap until you use your inhaler again.	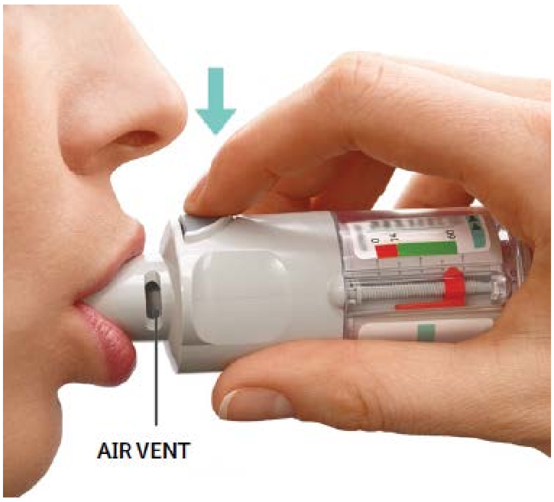

15.4 Instructions for the use of the Respimat® inhaler with spacer

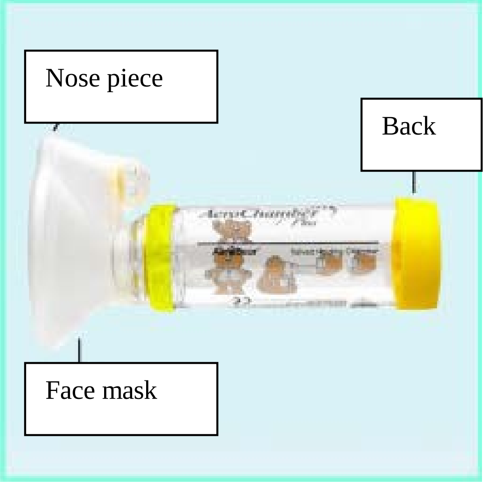

You will need to use this inhaler only ONCE A DAY.
Each time it is used take 1 PUFFS to achieve proper dosing.

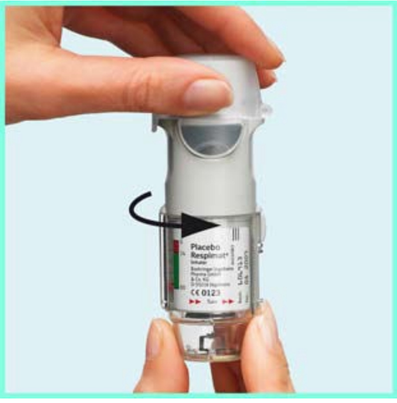

I Hold the Respimat® inhaler upright with the cap closed to avoid accidental release of the dose. Turn the clear base in the direction of the red arrows on the label until it clicks (half a turn).

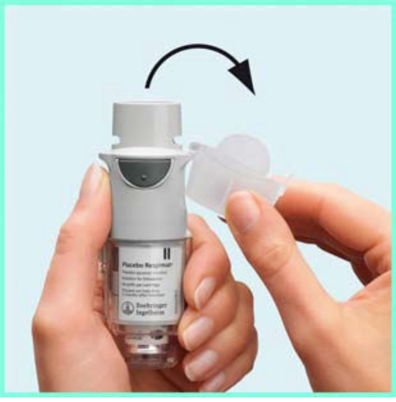

II Open the cap (A) until it snaps fully open

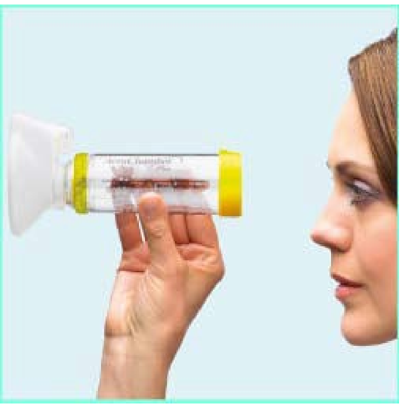

III Now take the spacer.

Look inside for foreign objects.

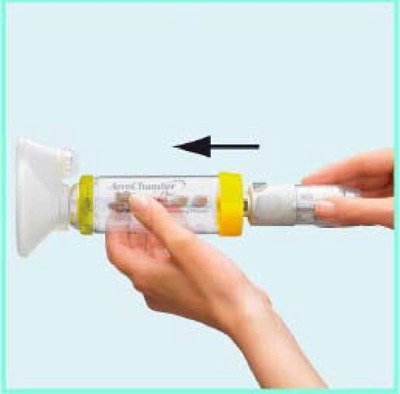

IV Insert the mouthpiece of the Respimat® inhaler into the back of the spacer.

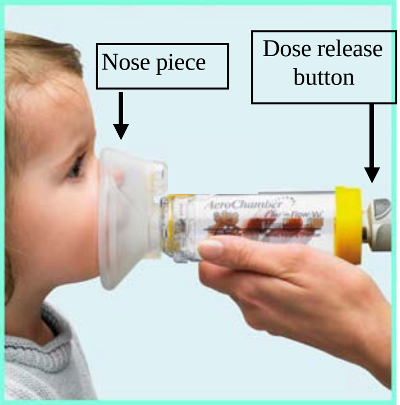

V Make sure the nose piece of the spacer and the dose release button are aligned and upright.

Apply the face mask to the face of the child, making sure to obtain a good seal between the face and the spacer

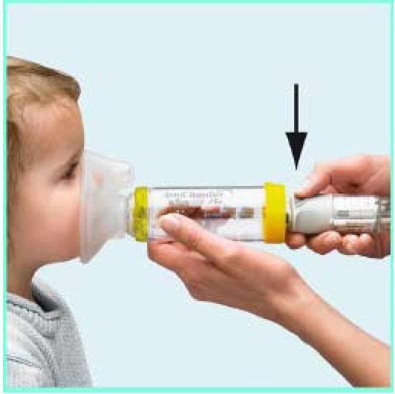

VI When the child starts breathing slowly and steadily, press the dose release button (D).

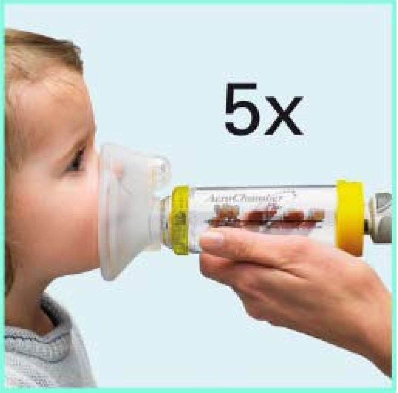

VII Maintain good seal over your child's mouth and nose for 5 breaths after pressing the dose release button. Take care not to get the spray into the child's eyes.

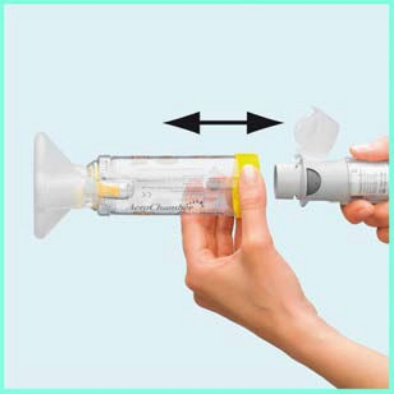

VIII Pull the spacer and the Respimat® inhaler apart and close the cap of Respimat® inhaler. Now repeat all steps (I–VIII) one more time for a second inhalation to ensure your child receives the full daily dose.

At the completion of the second puff, close the cap until the inhaler is used again.

<u>When to get a new Respimat® inhaler</u>

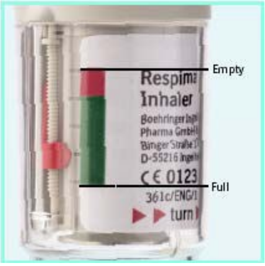

The Respimat® inhaler contains 30 doses (60 puffs) after priming. The dose indicator shows approximately how many doses are left. When the pointer enters the red area of the scale, there is, approximately, medication for 7 days (14 puffs) left. This is when you need to get a new Respimat® inhaler.

Once the dose indicator has reached the end of the red scale (i.e. all 30 doses have been used), the Respima® inhaler locks automatically. At this point, the base cannot be turned any further.

At the latest, three months after first use the Respimat® inhaler should be discarded even if not all medication has been used.

What if...

What if...	Reason	What to do
I cannot turn the base easily.	a) The Respimat® inhaler is already prepared and ready to use.<br>b) The Respimat® inhaler is locked after 60 puffs (30 doses).	a) The Respimat® inhaler can be used as it is.<br>b) Prepare and use your new Respimat® inhaler.
I cannot press the dose release button.	The clear base has not been turned.	Turn the clear base until it clicks (half a turn).
The clear base springs back after I have turned it.	The clear base was not turned far enough.	Prepare the Respimat® inhaler for use by turning the clear base until it clicks (half a turn).
I can turn the clear base past the point where it clicks.	Either the dose release button has been pressed, or the clear base has been turned too far.	With the gray cap closed, turn the base until it clicks (half a turn).

How to care for your inhaler

Clean the mouthpiece including the metal part inside the mouthpiece with a damp cloth or tissue only, at least once a week.

Any minor discoloration in the mouthpiece does not affect the performance of your Respimat® inhaler.

If necessary, wipe the outside of your Respimat® inhaler with a damp cloth.

How to care for the spacer

For taking care of the spacer please follow the cleaning instructions on the packaging insert which is enclosed to the original packaging.

Further information

The Respimat® inhaler must not be disassembled after inserting the cartridge and replacing the clear base.

Do not touch the piercing element inside the base.
Keep out of the reach and sight of children.
Do not freeze.

15.5 Pediatric asthma caregiver diary (PACD) and instructions

The PACD will be provided as an APP for the caregivers. Daily symptoms will be recorded from visit 1-visit 3 independent from the months of recruitment for 20 weeks. The PACD will be stopped in the summer for 4 months from May-end of August and restarted at the first of September.

Caregiver Asthma Diary—Caregiver Instructions

Try to answer all questions as best you can. It is very important that you do not skip any questions.

This diary consists of two parts - Overnight Symptoms and Daytime Symptoms.

There are 2 questions about your child's use of β-Agonist medication. β-Agonist has several different names. For your child, β-Agonist is called:

β-Agonist =

(TO BE FILLED IN BY YOUR NURSE/COORDINATOR)

Question number 8 asks about treatment with a systemic steroid (such as prednisone or prednisolone). For your child, the systemic steroid is called:

Systemic steroid =

(TO BE FILLED IN BY YOUR NURSE/COORDINATOR)

The diary begins with the Overnight Symptoms (Questions #1 to #3)

Overnight Symptoms: Enter your answers when your child wakes up:

These questions cover the time from when you put your child to bed for the night to the time when he/she wakes up in the morning.

For question #1 (How much did your child cough last night ....?), please try not to check the "I do not know" response unless you really were not able to hear your child coughing.

We are asking you about coughing episodes not about individual coughs. You do not need to count the number of coughs. We would like you to give us your impression of the amount of coughing that your child had during the night.

Please fill in the number of times that your child received β-Agonist from the time that he/she was put to bed until he/she got up in the morning. Please enter the **total** amount that your child was given during this time for each type of β-Agonist used (number of puffs, nebulized treatments, teaspoons or tablets). If tablets were given, record the dose in milligrams in the comments section. If no treatments were given, fill in "0" for each type of β-Agonist.

The diary continues with the Daytime Symptoms (Questions #4 to #10)

Daytime Symptoms: Enter your answers after you put your child to bed for the night:

These questions cover the time from when your child wakes up in the morning until you put him/her to bed for the night.

If your child is not with you at any time during the day, you should ask the person with whom your child spent the day about the problems your child had with asthma while you were not there.

Please fill in the number of times that your child received β-Agonist from the time that he/she got up in the morning until he/she was put to bed for the night. Please enter the **total** amount that your child was given during this time for each type of β-Agonist used (number of puffs, nebulized treatments, teaspoons or tablets). If tablets were given, record the dose in milligrams in the comments section. If no treatments were given, fill in "0" for each type of β-Agonist.

Copyright © 1997 Merck & Co., Inc.
Whitehouse Station, N.J., U.S.A. - All Rights Reserved.

Caregiver Asthma Diary (Overnight)

Date completed	month/day/year

Complete in the Morning

ANSWER IN THE MORNING: These questions cover the period of time from when your child went to bed for the night to when he/she awoke this morning.

1. How **much** did your child **cough** last night **after** your child was put to **bed for the night** until he/she awoke this morning? *(Check **one** response)*

Did not cough at all	Coughed very little	Coughed several times	Coughed frequently	Coughed almost all night	I do not know
⓪	①	②	③	④	⑤

2. How many times did you give your child β-Agonist since he/she went to bed last night? *(If your child did not wake up last night due to asthma, then you should fill in "0".)*

   Number of times:

3. How **many puffs, nebulizer treatments, teaspoons** or **tablets** of β-Agonist did your child use since he (she) was put to bed for the **night until he (she) awoke this morning**? **For each kind** of **β-Agonist** used, fill in the total number of puffs, nebulizer treatments teaspoons and tablets used. *(If your child did not wake up last night due to asthma, then you should fill in "0".)*

   β-Agonist inhaler: ______________ number of puffs

   β-Agonist by nebulizer: ______________ number of treatments

   Oral β-Agonist syrup/tablets: ______________ number of teaspoons or tablets

Comments:

I confirm that the information on this page is accurate:	Caregiver initial:	Date:

Copyright © 1997 Merck & Co., Inc.
Whitehouse Station, N.J., U.S.A. - All Rights Reserved.

Caregiver Asthma Diary (Daytime)

Date completed	month/day/year

Complete at Bedtime

ANSWER RIGHT AFTER YOUR CHILD GOES TO BED FOR THE NIGHT: These questions cover the period of time since your child awoke this morning for the day.

4. How **severe** was your child's **cough** today? *(Check **one** response)*

No cough	Very mild cough	Mild cough	Moderate cough	Severe cough	Very severe cough
⓪	①	②	③	④	⑤

5. How **severe** was your child's **wheezing** today? *(Check **one** response)*

No wheezing	Very mild wheezing	Mild wheezing	Moderate wheezing	Severe wheezing	Very severe wheezing
⓪	①	②	③	④	⑤

6. How **severe** was your child's **trouble breathing** today? *(Check **one** response)*

No trouble breathing	Very mild trouble breathing	Mild trouble breathing	Moderate trouble breathing	Severe trouble breathing	Very severe trouble breathing
⓪	①	②	③	④	⑤

7. How **much** did your child's asthma symptoms interfere with your **child's activities** today? *(Your **child's activities** could include any sort of physical activity such as running, playing, jumping, sports, bike-riding, climbing etc. or school activities) (Check **one** response)*

Did not interfere	Very mildly interfered	Mildly interfered	Moderately interfered	Severely interfered	Very severely interfered
⓪	①	②	③	④	⑤

8. Did your child visit a doctor, **emergency room**, or **hospital** for **asthma symptoms** (other than a scheduled visit to a doctor) or was your child **treated with a systemic steroid such as prednisone or prednisolone (by oral, intravenous, intramuscular, or rectal administration)** during the **previous 24 hours?** *(Check **one** response)*

No ⓪ Yes ① → If **yes**, check **all** that apply:

Visited a doctor	Visited an Emergency Room	Admitted to the Hospital overnight	Treated with a systemic steroid
②	③	④	⑤

9. How **many times** did you give your child **β-Agonist** since he (she) **awoke this morning**?
(If your child did not use any β-Agonist since waking up this morning, fill in "0".)

Number of times:

10. How **many puffs, nebulizer treatments, teaspoons** or **tablets** of β-Agonist did your child use since **he/she woke up** this **morning**? For each kind of β-Agonist used, fill in the **total number** of puffs, nebulizer treatments, teaspoons and tablets used. *(If your child did not use any β-Agonist since waking up this morning, fill in "0".)*

β-Agonist inhaler: ______________ number of puffs

β-Agonist by nebulizer: ______________ number of treatments

Oral β-Agonist syrup/tablets: ______________ number of teaspoons or tablets

I confirm that the information on this page is accurate:	Caregiver initial:	Date:

Copyright © 1997 Merck & Co., Inc.
Whitehouse Station, N.J., U.S.A. - All Rights Reserved.
