# Supplementary figures and images for: Characteristics of children with severe preschool asthma prior to starting the TIPP study
Source: Front Pediatr. 2025 Mar 5;13:1558256. doi: 10.3389/fped.2025.1558256 (PMC11921963; doi:10.3389/fped.2025.1558256)

Enrollment of patients into the TIPP study by month and Asthma Level

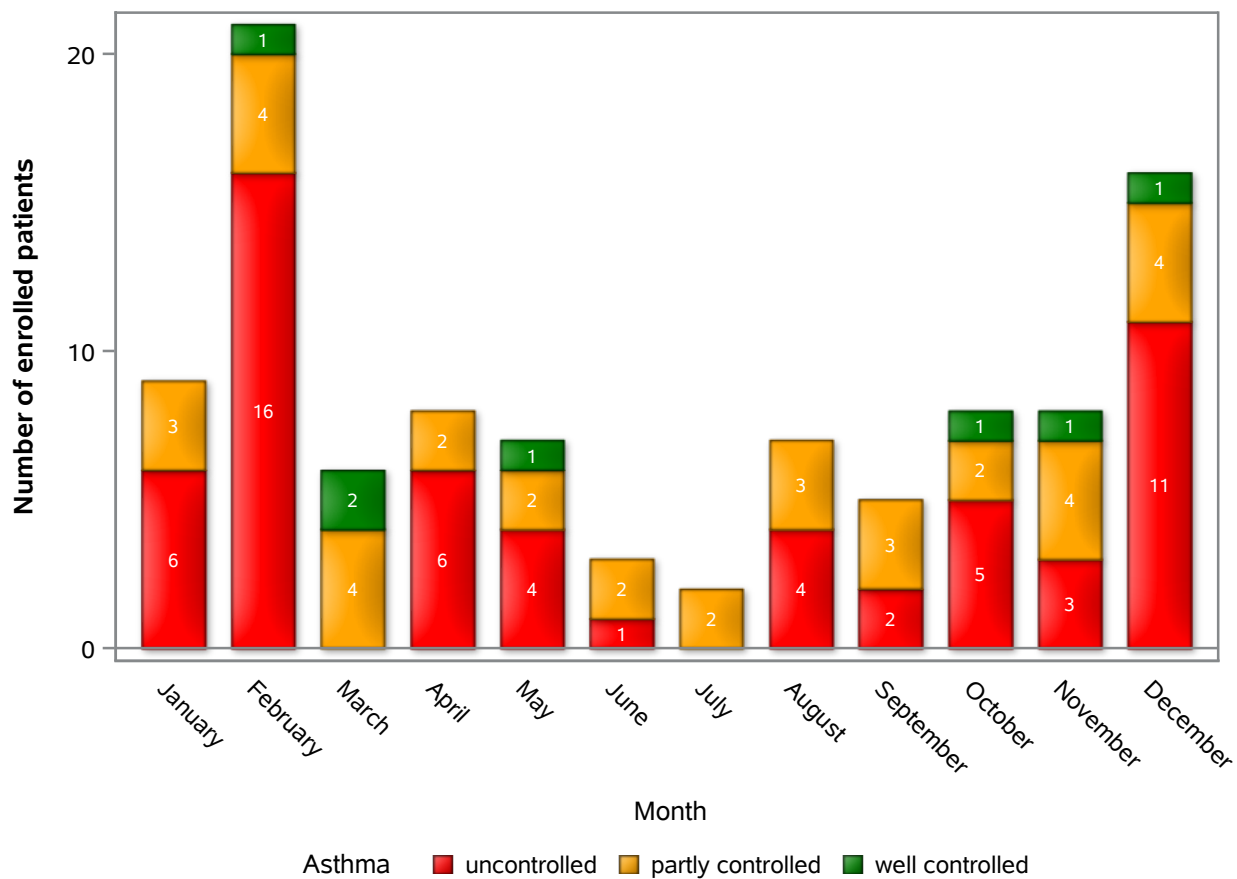

Supplement: Supplementary Figure S1 — GINA level in relation to months of enrollment. [file Datasheet2.pdf]
